# Prediction of high-flow nasal cannula failure in COVID-19 acute hypoxemic respiratory failure using combined clinical and metabolomic data: a machine learning study

**DOI:** 10.3389/fmed.2026.1946899

**Published:** 2026-10-30

**Authors:** Francisco José Parrilla-Gómez, Pau Nebot, Sergio Oller-Moreno, Judith Marin-Corral, Andrea Castellví-Font, Clara Vilà-Vilardell, Purificación Pérez-Terán, Aleix Granado-García, Rosana Muñoz-Bermúdez, Llesmil Ahuir-Bauset, Cristina Soriano Rodriguez, Carles Camañes Mayordomo, Noemí Haro, Alex Gomez-Gomez, Eva Martín-López, Oscar J. Pozo, Santiago Marco, Joan Ramon Masclans Enviz

**Affiliations:** 1 Department of Critical Care, Hospital del Mar, Barcelona, Spain; 2 Critical Illness Research Group (GREPAC), Hospital del Mar Research Institute (HMRI), Barcelona, Spain; 3 Department of Medicine and Life Sciences (MELIS), Universitat Pompeu Fabra (UPF), Barcelona, Spain; 4 Applied Metabolomics Research Group, Hospital del Mar Research Institute (HMRI), Barcelona, Spain; 5 Signal and Information Processing for Sensing Systems, Institute for Bioengineering of Catalonia–The Barcelona Institute of Science and Technology (IBEC-BIST), Barcelona, Spain; 6 Department of Electronics and Biomedical Engineering, Universitat de Barcelona, Barcelona, Spain

**Keywords:** COVID-19, failure, HFNC (high-flow nasal cannula), metabolomics, non invasive respiratory support

## Abstract

**Background:**

High-flow nasal cannula (HFNC) is widely used as first-line non-invasive respiratory support in acute hypoxemic respiratory failure (AHRF), particularly during the COVID-19 pandemic. However, HFNC failure remains frequent, and delayed intubation is associated with worse outcomes. Accurate early prediction of HFNC failure is therefore essential to guide clinical decision-making.

**Methods:**

We conducted a prospective observational cohort study including 111 patients with COVID-19-related AHRF treated with HFNC in a single tertiary ICU. Patients were divided into a Training Cohort (March 2020–April 2021; *n* = 89) and a temporally independent Validation Cohort (May 2021–October 2021; *n* = 22). A total of 164 variables (25 clinical and 139 metabolomic features) were collected at baseline. Predictive models were developed using Random Forest machine learning with recursive feature elimination and bootstrap resampling. Model performance was assessed using the area under the receiver operating characteristic curve (AUC), with internal validation and temporally independent validation.

**Results:**

The six-variable model integrating clinical and metabolomic data achieved an AUC of 0.87 (95% CI: 0.79–0.94) in the Training Cohort and 0.91 (95% CI: 0.78–1.00) in the Validation Cohort. A simplified three-variable model (SpO₂/FiO₂ ratio, anthranilic acid, and octanoic acid) achieved an AUC of 0.71 (95% CI: 0.61–0.82) in the Training Cohort and 0.91 (95% CI: 0.77–1.00) in the Validation Cohort. Both models showed improved performance compared with the ROX index in the Training Cohort. Performance in the Validation Cohort should be interpreted cautiously due to limited sample size.

**Conclusions:**

A predictive model combining clinical and metabolomic data showed promising performance for identifying HFNC failure in COVID-19-related AHRF. A simplified model using only three variables retained comparable performance in the validation cohort, supporting further evaluation of its potential clinical utility. Further validation in larger and more diverse populations is required.

## Introduction

High-flow nasal cannula (HFNC) has become a widely used first-line non-invasive respiratory support strategy for patients with hypoxemic acute respiratory failure (AHRF), particularly during the COVID-19 pandemic (1–5). Compared with conventional oxygen therapy, HFNC has been associated with improved oxygenation and reduced need for invasive mechanical ventilation in selected patients (6–10).

However, HFNC failure remains frequent, and delayed intubation in this setting has been consistently associated with worse outcomes, including increased mortality (11).

Several tools have been proposed to predict HFNC outcomes, including the ROX index and other clinical scores. While these approaches are simple and readily available at the bedside, their predictive performance is variable and may depend on timing and patient population. Moreover, they rely exclusively on conventional clinical variables and may not capture the underlying biological heterogeneity of AHRF (12–14).

Metabolomics provides a comprehensive characterization of metabolic pathways and has shown potential in identifying disease phenotypes (15–17), assessing severity (18–23), and predicting outcomes in critical illness, including AHRF and acute respiratory distress syndrome (24–28). However, its application to predict response to specific respiratory support strategies, such as HFNC, remains largely unexplored.

We hypothesize that integrating clinical parameters with metabolic biomarkers could improve prediction of HFNC failure in patients with COVID-19-related AHRF. The aim of this study was to develop and validate a predictive model combining clinical and metabolomic data to identify patients at risk of HFNC failure. This approach may provide a framework for physiology-informed and biology-informed decision-making in AHRF.

## Methods

### Study design and population

We conducted a prospective observational cohort study in the medical intensive care unit (ICU) of Hospital del Mar (Barcelona, Spain) between March 2020 and October 2021. Adult patients with AHRF due to SARS-CoV-2 infection, confirmed by PCR, and treated with HFNC within 48 h of hospital admission were eligible. Data were accessed for research purposes between 01/03/2021 and 30/06/2022.

Patients were selected based on severity criteria, including tachypnea (respiratory rate ≥30 breaths/min) and/or persistent hypoxemia (SpO₂/FiO₂ ≤ 200), requiring ICU team assessment. Patients with pre-existing ‘do not resuscitate’ orders were excluded. All patients received standard-of-care treatment according to institutional protocols.

The study population was divided into two temporally distinct cohorts to enable temporal validation: a Training Cohort including patients admitted between March 2020 and April 2021 used for model development, and a Validation Cohort including patients admitted from May 2021 to October 2021 used for temporally independent validation. This approach allowed model evaluation across different pandemic waves and clinical contexts.

The study protocol was approved by the local Research Ethics Committee (2020/9657/I), and due to exceptional circumstances of the COVID-19 pandemic, trial registration was completed retrospectively (ClinicalTrials.gov identifier: NCT06695442). Written informed consent was obtained from all participants or their legally authorized representatives prior to study inclusion.

### Data collection and variables

Clinical and biological data were extracted from electronic medical records [IMASIS system (29),] at study inclusion (baseline, T0), including demographic characteristics, comorbidities, severity scores (APACHE II), respiratory parameters, vital signs, laboratory results, and treatment variables.

Plasma samples were collected at T0, processed, and stored at −80 °C until analysis. A total of 164 variables were recorded, including 25 clinical variables and 139 metabolomic features (111 metabolites and 28 metabolite ratios). Detailed definitions and distributions of all variables are provided in the Supplementary Material.

### Outcome definition

The primary outcome was HFNC failure, defined as the need for endotracheal intubation during ICU stay.

### Metabolomic analysis

Metabolomic profiling was performed using liquid chromatography-tandem mass spectrometry (LC-MS/MS), applying six established analytical protocols targeting carboxylic acids, neurotransmitters, lipids, kynurenine pathway metabolites, and steroids (30–34).

To enhance interpretability and clinical feasibility of the secondary simplified model, correlation structure (to avoid redundancy), analytical robustness, and biological plausibility were considered after the primary statistical feature-selection procedure. Detailed analytical procedures, quality control, and preprocessing steps are described in the Supplementary Material.

### Data processing

Continuous variables were inspected for distribution and outliers prior to analysis. Given the use of tree-based machine learning methods (Random Forest), no explicit feature scaling or normalization was required, as these algorithms are invariant to monotonic transformations of predictors.

We found no specific missing data patterns that indicated selection bias across subgroups. Out of the 123 patients initially eligible for the study, 12 (9.8%) had at least one missing value among the candidate variables and were therefore excluded: 9 from the Development Cohort and 3 from the Validation Cohort. No individual variable had more than 5% missing data, and the missing data was limited to clinical variables. The metabolomic panel was complete for all patients. Because only 12 patients were excluded, any comparison between excluded and analysed patients would have limited statistical power; the assumption that data were missing completely at random therefore cannot be formally verified, although the small absolute number of exclusions constrains the potential impact on the analytical sample.

A complete-case analysis was conducted prior to model development. After excluding incomplete cases, the analytical dataset consisted of 111 patients and had no missing values among the candidate variables used for modeling. As a result, all 111 patients were available for cohort partitioning, feature selection, and modeling, and no imputation was needed.

### Model development and feature selection

Predictive modeling was performed using a Random Forest (RF) algorithm implemented in R (randomForest package version 4.7.1.1) (35).

Given the high dimensionality of the dataset and potential non-linear interactions, feature selection was conducted using a wrapper-based approach combining RF variable importance ranking with recursive feature elimination (RFE).

The process began with a full model including all candidate variables. Variables were ranked according to mean decrease in Gini impurity, and the least informative 20% were iteratively removed. This procedure was repeated until all variables had been evaluated.

To account for variability introduced by bootstrap sampling, the entire feature selection process was repeated 100 times, generating multiple candidate models with different variable combinations.

The 164 variables represented the initial candidate predictor space rather than the dimensionality of the final models. To limit optimism associated with high-dimensional model development, the complete data-dependent modeling procedure, including variable ranking, recursive feature elimination, model-complexity optimization, and model fitting, was performed within the internal-validation resampling procedure. Feature selection was therefore repeated independently within the corresponding training data at each resampling iteration.

The optimal number of features was determined by comparing AUC distributions across models of varying complexity. Although models with seven variables achieved the highest median AUC, six-variable models demonstrated comparable performance without significant loss of predictive accuracy and were therefore selected as the optimal trade-off between performance and parsimony (Figure 1).

**Figure 1:**
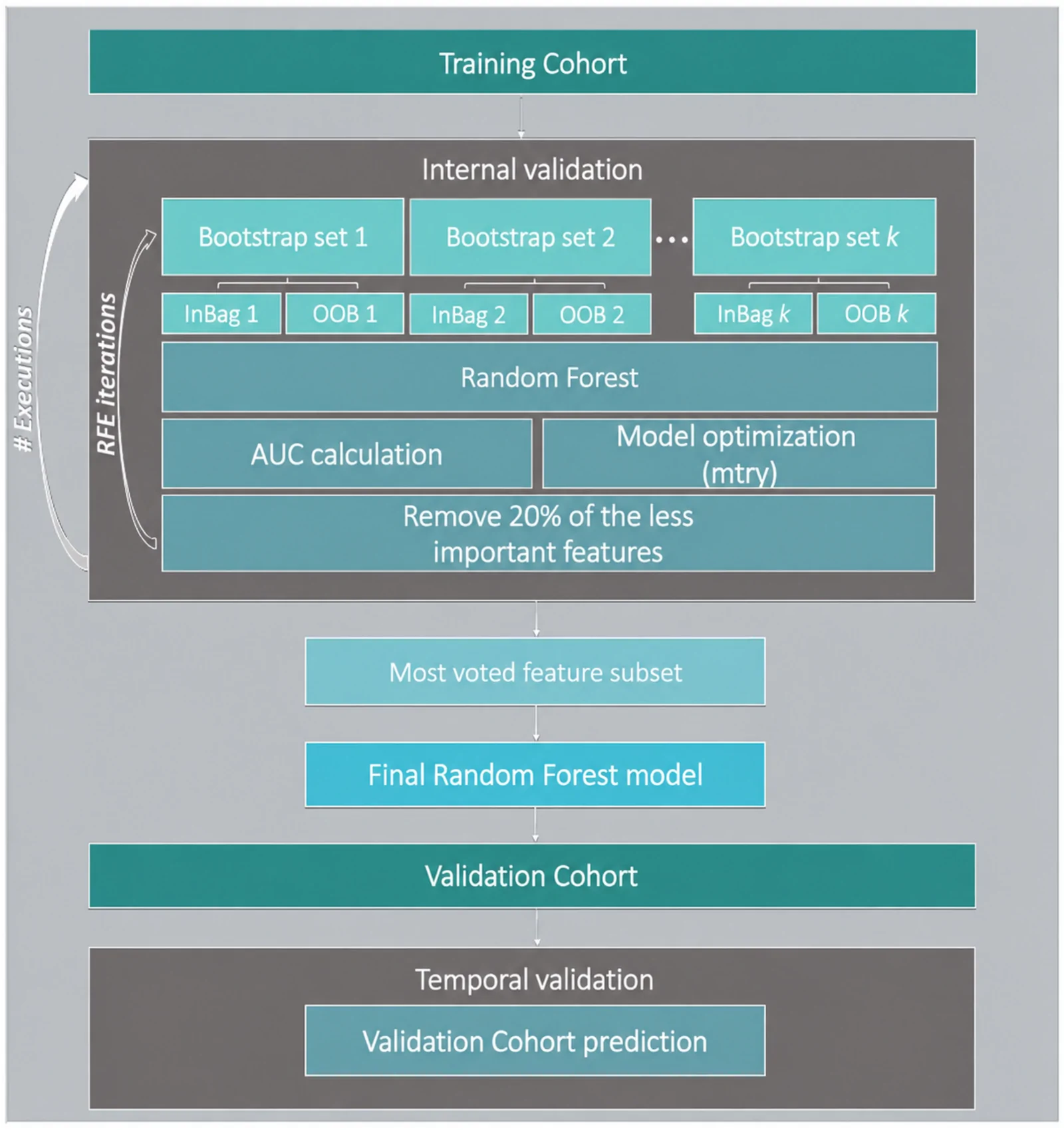
Model development and validation workflow. Workflow illustrating model development in the Training Cohort, including feature selection and hyperparameter optimization (*mtry*), followed by temporal validation in the temporally independent Validation Cohort. Model performance was assessed using the area under the receiver operating characteristic curve (AUC). RFE, recursive feature elimination.

Based on these results, two models were defined: 1) Best Prediction Model, six variables (clinical and metabolomic); and 2) Clinical Practice Model, three variables selected according to selection stability, quantified by frequency of inclusion across the resampling-based feature-selection iterations, together with clinical feasibility. Clinical feasibility was considered pragmatically according to measurement availability and timeliness, analytical robustness, potential implementation using a limited analytical platform, and the additional measurement burden required in the intended clinical setting.

### Model training, validation, and performance assessment

Random Forest uses bootstrap sampling internally for the construction of individual trees, which may help reduce model variance and limit overfitting within the training data.

The development and validation cohorts were kept strictly separated throughout the analysis. Internal validation was conducted within the Training Cohort, while temporal validation was performed using the temporally independent Validation Cohort, which was not used for feature selection, tuning, model comparison, threshold or model specification, or any other model-development decision, and remained unseen until the modeling procedure had been finalized. Model discrimination was assessed using the area under the receiver operating characteristic curve (AUC) with 95% confidence intervals. Additional performance metrics included sensitivity, specificity, F1-score, and optimal thresholds based on the Youden index (36).

To assess statistical significance of model performance in the validation cohort, permutation testing (1,000 iterations) was performed by randomly shuffling outcome labels to generate a null distribution of AUC values.

Comparisons between models and with the ROX index were conducted using paired bootstrap methods.

To account for potential covariate shifts between cohorts, overlap weighting based on propensity scores was applied as a sensitivity analysis (37).

### Model calibration and stability analysis

Calibration was assessed graphically by comparing predicted and observed probabilities in the Validation Cohort. Given the limited validation sample (*n* = 22), calibration plots were interpreted descriptively, since quantitative estimates of calibration intercept and slope would be expected to have substantial sampling uncertainty. Formal goodness-of-fit testing using the Hosmer-Lemeshow test was therefore not considered appropriate.

Model stability was further evaluated using repeated random data partitioning (100 iterations; 70% training, 30% testing), demonstrating consistent performance across different splits.

### Statistical analysis

Continuous variables are reported as mean ± standard deviation or median (interquartile range), as appropriate. Normality was assessed using the Shapiro–Wilk test. Group comparisons were performed using the Mann–Whitney U test or Wilcoxon test for continuous variables and the chi-squared test for categorical variables.

Multiple comparisons were adjusted using the Benjamini–Hochberg procedure (38), with statistical significance defined as *p*-adjusted <0.05.

All analyses were conducted using R software (version 4.3.0; R Foundation for Statistical Computing, Vienna, Austria).

The study is reported in conformance with the Strengthening Reporting of Observational studies in Epidemiology (STROBE) recommendations (39).

## Results

### Study population

A total of 111 patients were included, comprising 89 in the Training Cohort and 22 in the Validation Cohort (Figure 2).

**Figure 2:**
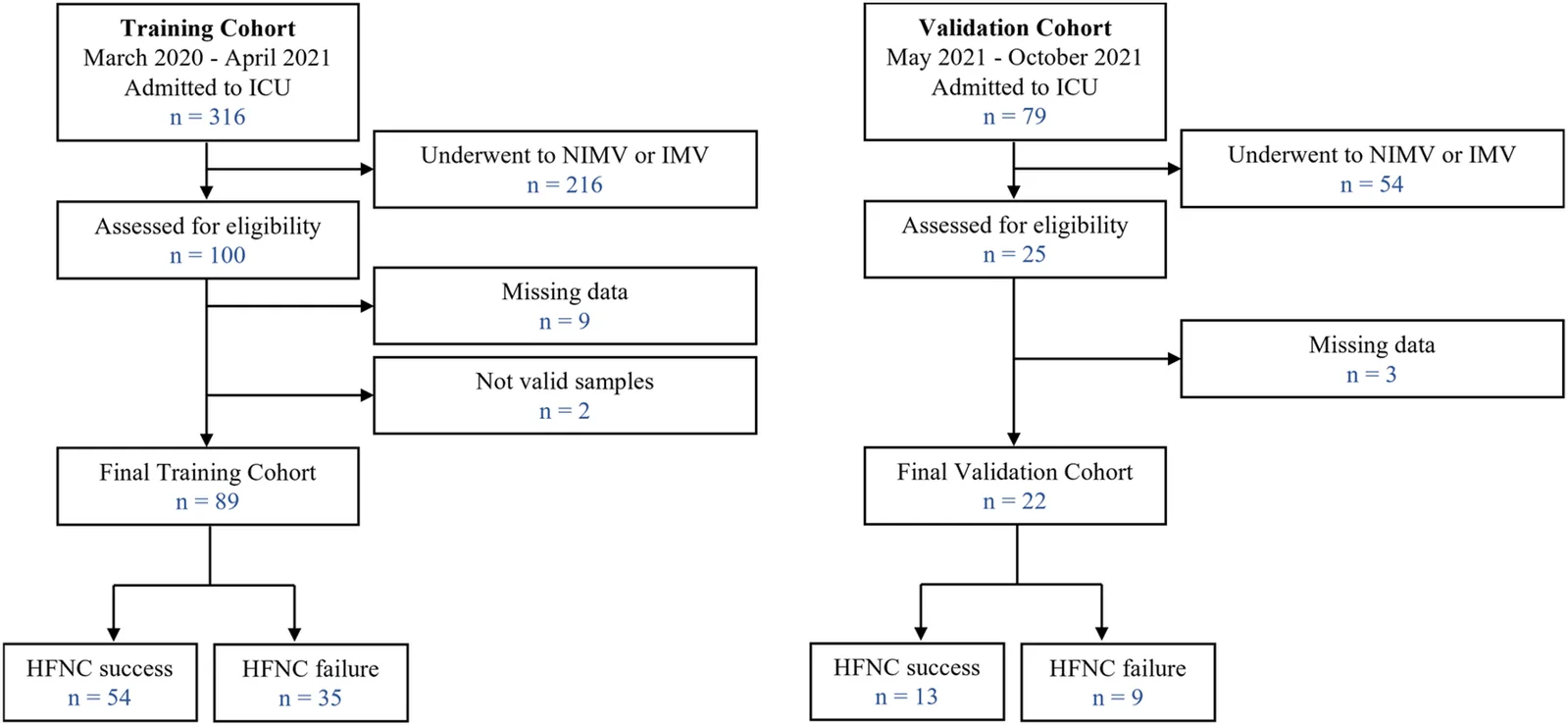
Study flow diagram. Flow diagram of patient screening, eligibility, and inclusion. NIMV, non-invasive mechanical ventilation; IMV, invasive mechanical ventilation.

Baseline characteristics are presented in Table 1. The cohorts differed in age, with a lower mean age in the Validation Cohort compared with the Training Cohort (50 ± 11 vs. 59 ± 11 years, *p* = 0.021), while other clinical characteristics were broadly comparable.

**Table 1:** Patient characteristics at inclusion (T0) for training and validation cohorts.

Variables	Training (*n* = 89)	Validation (*n* = 22)	*p*-value*
Age (years)	59 ± 12	50 ± 11	**0**.**021**
Gender (female), *n* (%)	30 (33.71)	9 (40.91)	0.830
Obesity, *n* (%)	13 (14.61)	5 (22.73)	0.830
Diabetes Mellitus, *n* (%)	17 (19.1)	4 (18.18)	1.000
Dyslipidemia, *n* (%)	26 (29.21)	3 (13.64)	0.480
Arterial Hypertension, *n* (%)	43 (48.31)	4 (18.18)	0.130
BMI (Kg/m^2^)	28 ± 5	29 ± 9	1.000
Symptom days prior to D1	10 ± 6	11 ± 9	0.980
Number of comorbidities	1.37 ± 1.34	1.14 ± 1.21	0.820
APACHE II Score	14 ± 6	14 ± 6	0.950
Norepinephrine (>0.1 μg/kg/min), *n* (%)	40 (44.94)	9 (40.91)	1.000

BMI, body mass index; APACHE II, acute physiology and chronic health evaluation.

*Values reflect *p*-values adjusted using the Benjamini & Hochberg method.

No significant differences were observed in vital signs, oxygenation indices, admission timing, or initial HFNC settings (Table 2). HFNC failure rates were comparable across cohorts (39% in the Training Cohort vs. 41% in the Validation Cohort, *p* = 1.000).

**Table 2:** Clinical characteristics on T0: training and validation cohorts.

Variables	Training (*n* = 89)	Validation (*n* = 22)	*p*-value*
Respiratory condition and vital signs			
Heart rate (bpm)	77 ± 16	71 ± 15	0.400
Mean Arterial pressure (mmHg)	91 ± 17	89 ± 12	0.900
Respiratory rate (RR, breaths/min)	23 ± 8	25 ± 8	0.760
SpO₂ (%)	94 ± 4	94 ± 3	0.970
HFNC flow (L/min)	59 ± 4	55 ± 13	0.110
HFNC FiO_2_ (ratio)	65 ± 13	66 ± 16	0.840
SatO_2_/F_I_O_2_ (ratio)	153 ± 40	152 ± 43	0.880
ROX index	7 ± 4	7 ± 3	0.840
Arterial blood gases			
pH	7.43 ± 0.06	7.47 ± 0.03	0.040
PaCO₂ (mmHg)	34.18 ± 6.35	33.35 ± 3.7	0.790
PaO₂ (mmHg)	89.14 ± 50.21	68.24 ± 25.38	0.300
HCO₃- (mmol/L)	23.48 ± 3.51	24.83 ± 1.8	0.540
Base excess (mmol/L)	−1.9 ± 5.19	−0.06 ± 2.27	0.560
SatO₂ (%)	93.75 ± 6.47	91.19 ± 9.1	0.430
PaO₂/FiO₂	148.02 ± 112.75	111.42 ± 46.69	0.330
Lactate Plasma (mmoL/L)	1.68 ± 0.65	1.61 ± 0.57	0.870
Timing delays			
Symptoms start to ICU admission (days)	10 (7–11)	9 (7–13)	1.000
Hospital admission to ICU admission (days)	3 (1–6)	2 (1–4)	0.890
Hospital admission to T0 (days)	3 (1–6)	2 (1–4)	0.790
Medications			
Vaccination status (complete), *n* (%)	0 (0)	1 (4.55)	0.830
Remdesivir, *n* (%)	15 (20.83)	0 (0)	0.500
Tocilizumab, *n* (%)	25 (34.72)	4 (22.22)	0.830
Hydroxychloroquine, *n* (%)	13 (18.06)	0 (0)	0.500
Dexamethasone, *n* (%)	62 (86.11)	18 (100)	0.580

SpO₂, saturation of peripheral oxygen; HFNC, high-flow nasal cannula; FᵢO₂, fraction of inspired oxygen; SatO₂, peripheral oxygen saturation; ROX, ratio of oxygen saturation; PaCO₂, carbon dioxide partial pressure in arterial blood gas sample; PaO₂, oxygen partial pressure in arterial blood gas sample; HCO₃-, bicarbonate.

*Values reflect *p*-values adjusted using the Benjamini & Hochberg method.

### Model development and selected variables

A total of 164 variables (25 clinical and 139 metabolomic features) were considered for model development. Through recursive feature elimination with bootstrap resampling, models with seven variables achieved the highest median AUC; however, six-variable models demonstrated comparable performance without significant differences and were therefore selected as the optimal configuration (Figure 3).

**Figure 3:**
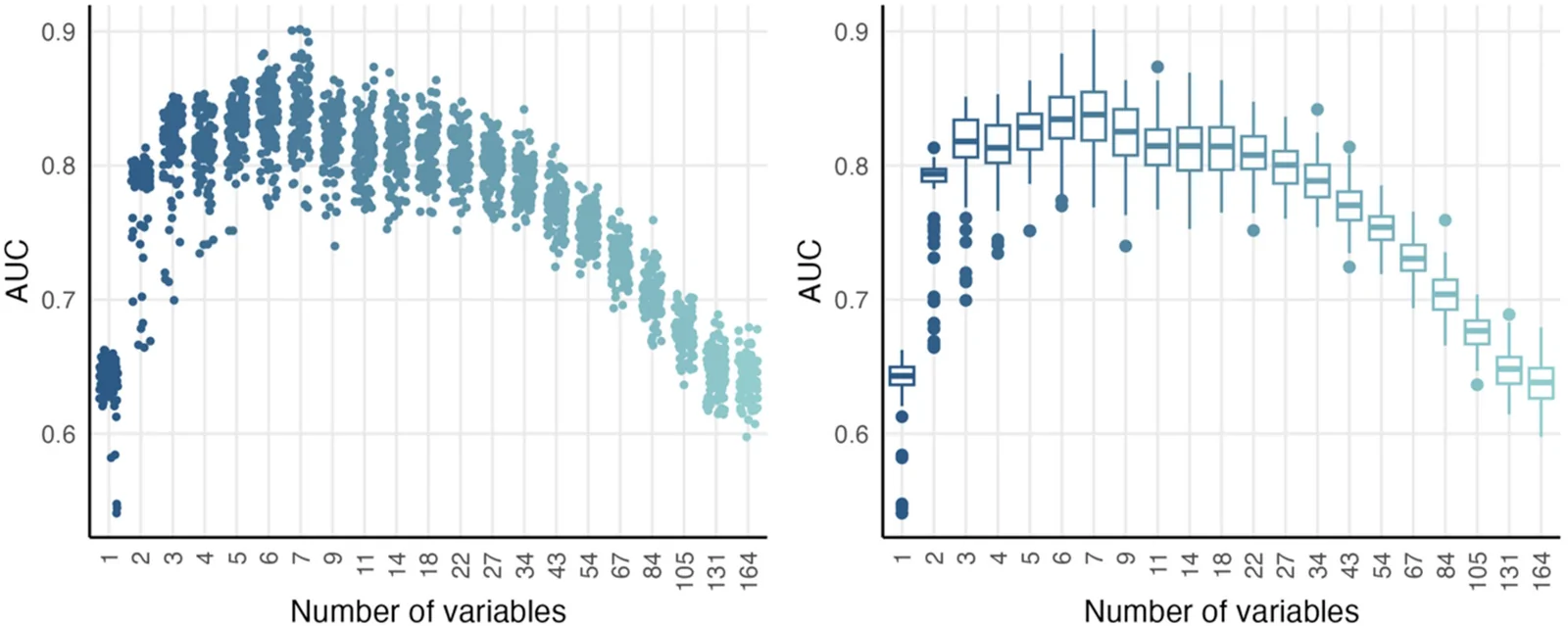
Model performance across different numbers of variables. Distribution of AUC values across models with varying numbers of variables evaluated through 100 iterations of recursive feature elimination (RFE) in the training cohort. Left panel: individual model performance; right panel: distribution summarized as boxplots.

The six-variable model (‘Best Prediction model’) included the following variables: SpO₂/FiO₂ ratio, anthranilic acid, octanoic acid, 20*β*-dihydrocortisone, DAG(16:1), and DAG(18:0/20:4). These variables were consistently identified across more than 40% of the top-performing models.

To improve clinical applicability, a simplified three-variable model (‘Clinical Practice model’) was also derived, including SpO₂/FiO₂ ratio, anthranilic acid, and octanoic acid.

### Model performance

In the Training Cohort, the Best Prediction model achieved an AUC of 0.87 (95% CI: 0.79–0.94), while the Clinical Practice model achieved an AUC of 0.71 (95% CI: 0.62–0.82).

In the Validation Cohort, both models demonstrated high discrimination, with AUCs of 0.91 (95% CI: 0.78–1.00) for the Best Prediction model and 0.91 (95% CI: 0.77–1.00) for the Clinical Practice model (Figure 4).

**Figure 4:**
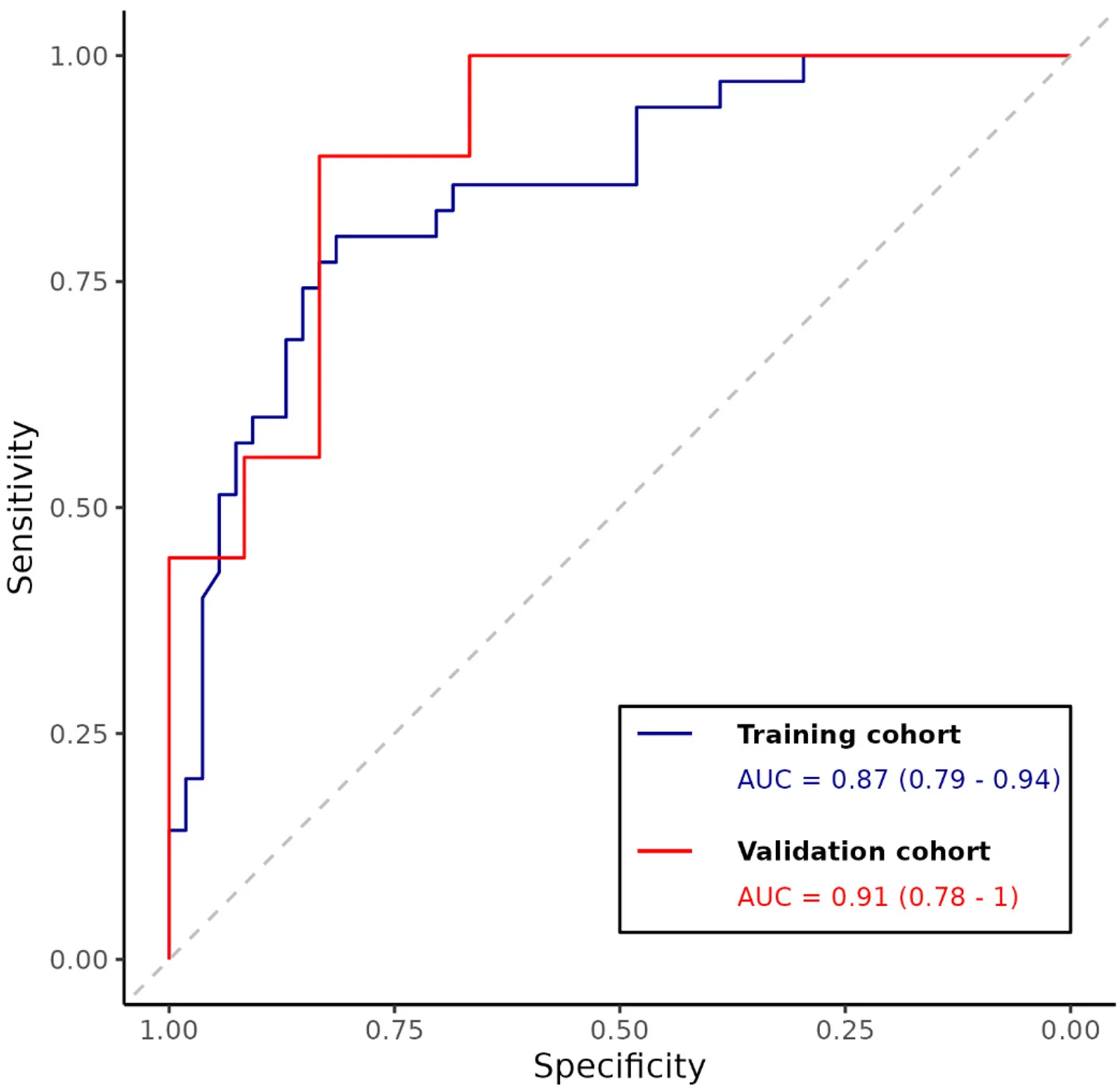
ROC curves for the best prediction model. Receiver operating characteristic (ROC) curves of the Best Prediction Random Forest model in the training cohort (blue) and validation cohort (red). The model includes SpO₂/FiO₂ ratio, anthranilic acid, octanoic acid, 20*β*-dihydrocortisone, DAG(16:1), and DAG(18:0/20:4). AUC, area under the receiver operating characteristic curve.

Additional performance metrics showed that, in the Training Cohort, the Best Prediction model achieved a sensitivity of 0.80 (95% CI: 0.77–0.83), specificity of 0.80 (95% CI: 0.76–0.83), and F1-score of 0.76, while the Clinical Practice model achieved a sensitivity of 0.63 (95% CI: 0.45–0.79), specificity of 0.76 (95% CI: 0.62–0.87), and F1-score of 0.63. In the Validation Cohort, both models achieved a sensitivity of 1.00 (95% CI: 0.72–1.00), with specificities of 0.75 (95% CI: 0.43–0.95) and 0.78 (95% CI: 0.40–0.95), and F1-scores of 0.85 and 0.86 for the Best Prediction and Clinical Practice models, respectively (Figure 5).

**Figure 5:**
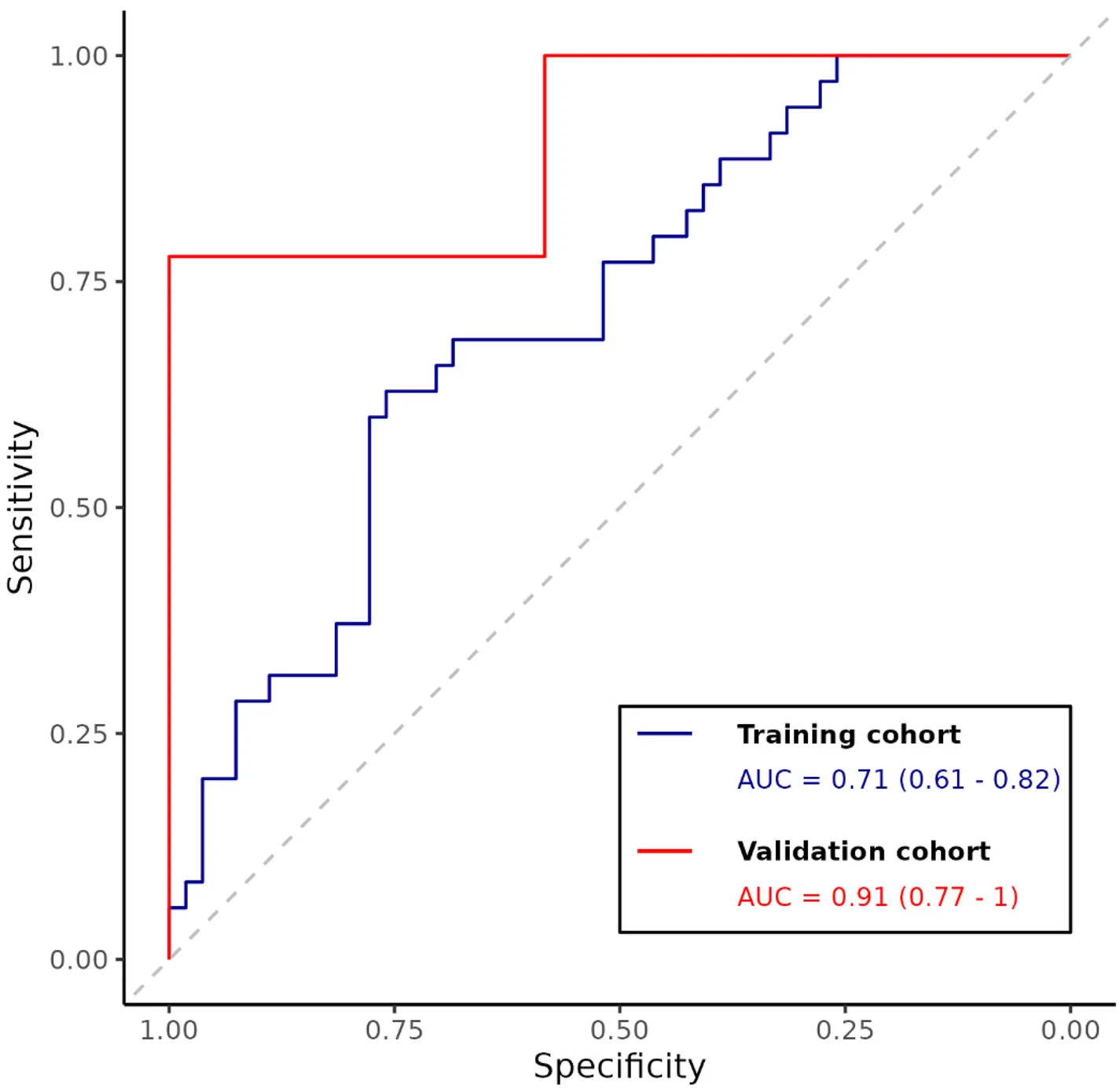
ROC curves for the clinical practice model. Receiver operating characteristic (ROC) curves of the simplified Random Forest model in the training cohort (blue) and validation cohort (red), including SpO₂/FiO₂ ratio, anthranilic acid, and octanoic acid. AUC, area under the receiver operating characteristic curve.

Given the limited validation sample (*n* = 22), these estimates should be interpreted with caution. In particular, the observed sensitivity of 1.00 represents the point estimate in this cohort and should not be interpreted as evidence of perfect sensitivity in the target population.

Permutation testing confirmed that model performance in the validation cohort was significantly greater than expected by chance (*p* < 0.001 for both models).

### Comparison with ROX index

Comparisons between the predictive models and the ROX index are shown in Figures 6,7.

**Figure 6:**
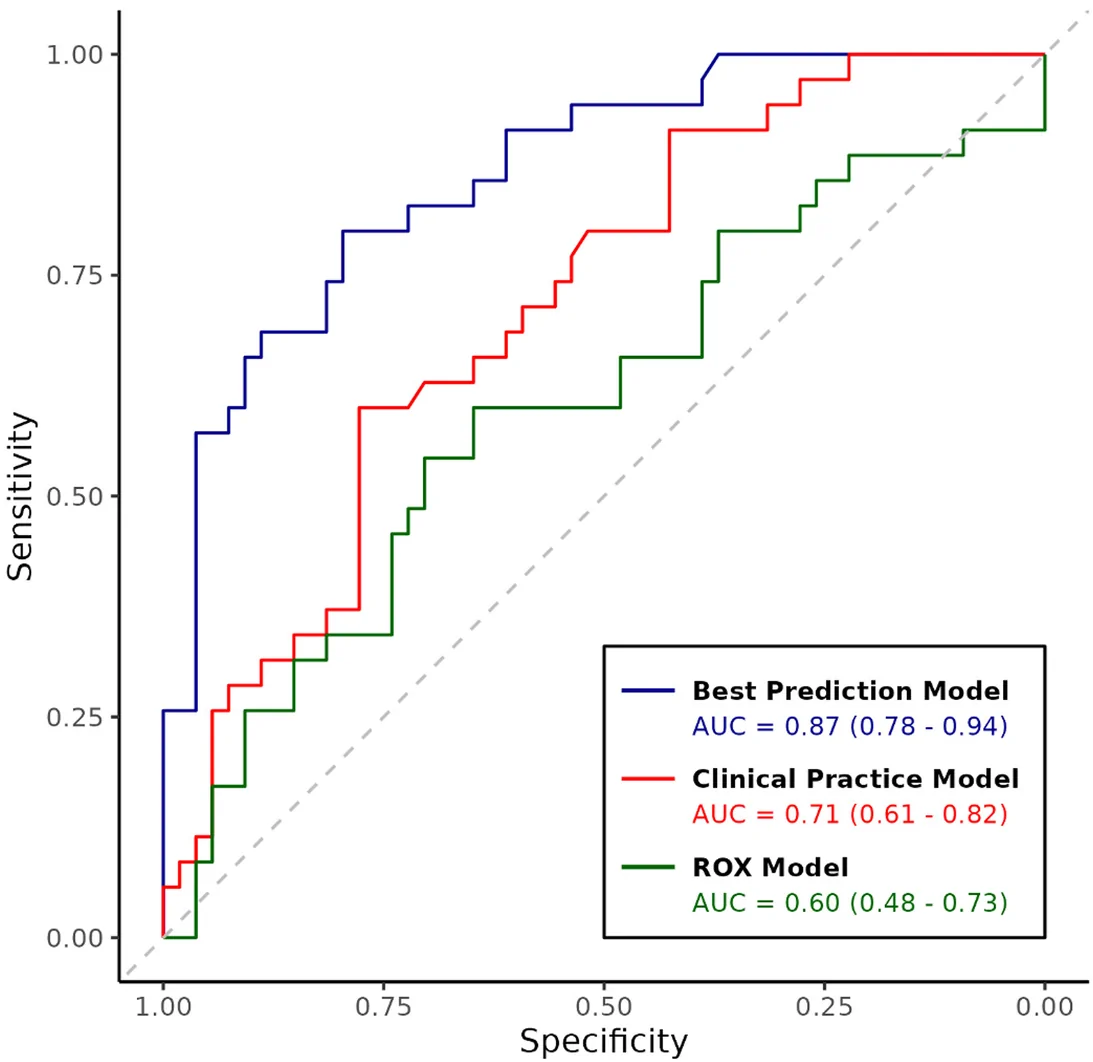
Model performance comparison in the training cohort. Comparison of ROC curves between the Best Prediction model, Clinical Practice model, and ROX index in the training cohort. The Best Prediction model significantly outperformed both the Clinical Practice model and the ROX index. AUC, area under the receiver operating characteristic (ROC) curve.

**Figure 7:**
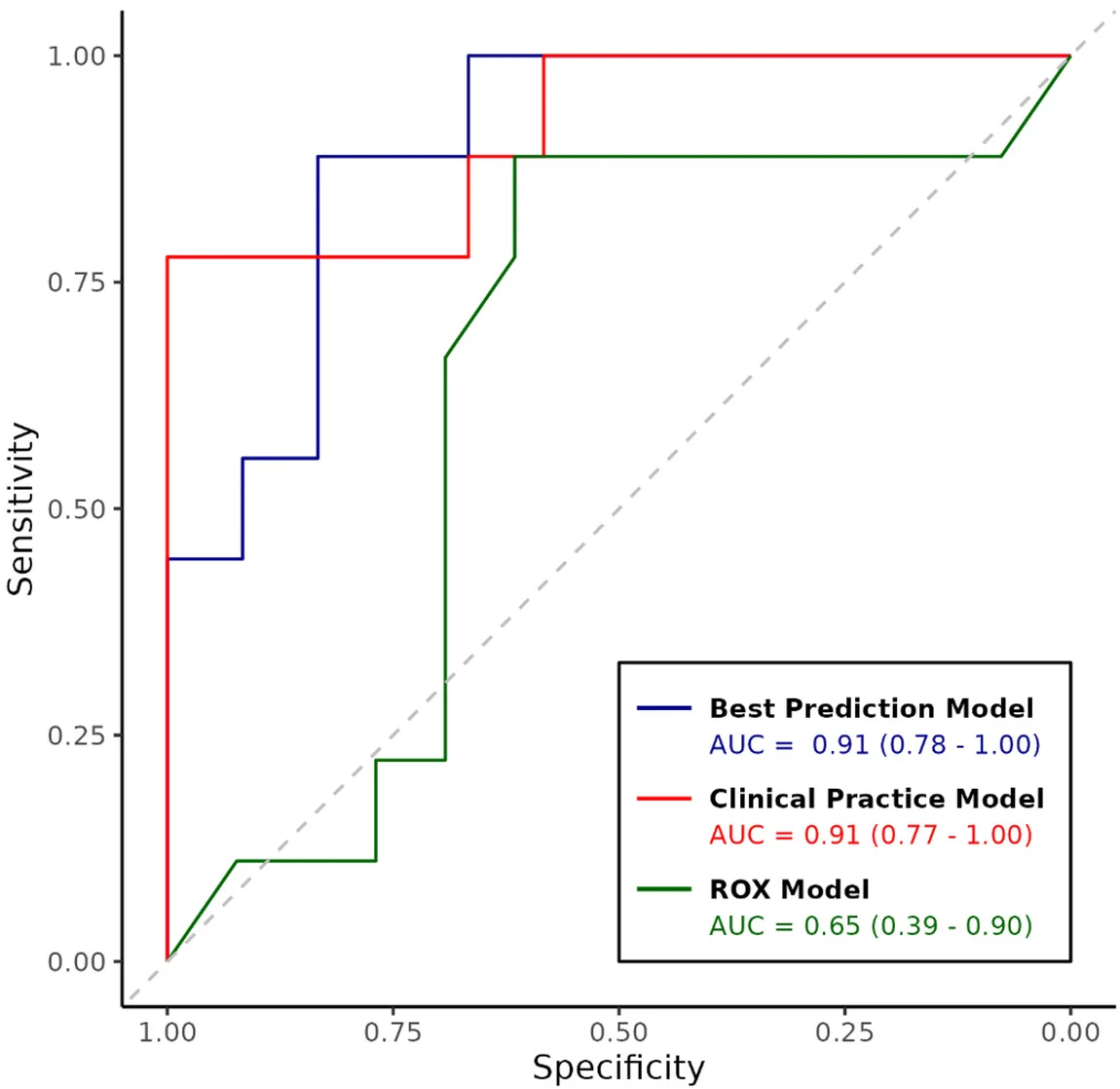
Model performance comparison in the validation cohort. Comparison of ROC curves between the Best Prediction model, Clinical Practice model, and ROX index in the validation cohort. Differences should be interpreted cautiously due to limited sample size. AUC, area under the receiver operating characteristic (ROC) curve.

In the Training Cohort, the Best Prediction model significantly outperformed the ROX index (*p* < 0.001; power=0.91) and the Clinical Practice model (*p* = 0.002; power=0.88). In contrast, no statistically significant difference was observed between the Clinical Practice model and the ROX index (*p* = 0.30; power=0.18).

In the Validation Cohort, both the Best Prediction model and the Clinical Practice model showed statistically significant differences compared with the ROX index (*p* = 0.032 and *p* = 0.025, respectively), although statistical power was limited (44%–50%) due to the small sample size.

### Model calibration and stability

Visual inspection of the calibration plots showed no obvious major discrepancy between predicted and observed probabilities; however, given the small validation cohort, these plots should be interpreted descriptively rather than as definitive evidence of model calibration.

Model performance remained consistent across repeated random data partitioning (100 iterations; 70% training, 30% testing), supporting the stability of the predictive models across different dataset splits.

### Metabolic findings

HFNC failure was associated with distinct metabolic patterns, including higher levels of kynurenine pathway metabolites (including anthranilic acid), medium-chain fatty acids (notably octanoic acid), and cortisol-related metabolites.

Additional methodological details, extended analyses, and complementary results, including sensitivity analyses and model development procedures, are provided in the Supplementary Material.

## Discussion

This study developed and validated a predictive model integrating clinical and metabolomic data to identify HFNC failure in patients with COVID-19-related AHRF. The main findings are: 1) a combined clinical-metabolomic model demonstrated high discriminative performance in both training and validation cohorts; and 2) a simplified model including only three variables retained comparable performance in the validation cohort, providing encouraging evidence that requires further evaluation in larger independent cohorts.

Several approaches have been proposed to predict HFNC failure, including the ROX index and other clinical scores. While these tools are simple and widely used, their predictive performance varies across studies and clinical settings (12). In COVID-19-related AHRF, reported AUC values for the ROX index range from moderate to good discrimination depending on timing and population (40–42). In our study, both the comprehensive and simplified models showed improved predictive performance compared with the ROX index, particularly in the training cohort, while differences in the validation cohort should be interpreted cautiously given the limited sample size and reduced statistical power.

The integration of metabolomic data provides a potential explanation for the improved predictive performance observed. The selected metabolites, including anthranilic acid and octanoic acid, are involved in pathways related to inflammation, energy metabolism, and mitochondrial function (43). In addition, broader metabolomic patterns associated with HFNC failure included alterations in the kynurenine pathway, medium-chain fatty acids, and cortisol metabolism, suggesting a link between systemic inflammatory response, metabolic dysregulation, and respiratory failure progression (44–48).

From a clinical perspective, the simplified model is particularly relevant, as it combines an easily accessible physiological parameter (SpO_2_/FiO_2_ ratio) with a limited number of metabolites, potentially enabling translation into clinical practice. The two selected metabolites (octanoic and anthranilic acids) belong to the same chemical family (organic carboxylic acids) and could potentially be measured within a single analytical platform, facilitating their future quantification through dedicated assays in conventional laboratory settings. Additionally, these metabolites appear to be less influenced by pre-analytical factors such as fasting status or timing of sample collection, which is particularly relevant in critically ill patients presenting under variable physiological conditions. Although metabolomic assays are not yet routinely available in critical care settings, the identification of a reduced set of robust and biologically relevant metabolites may facilitate the development of targeted and clinically applicable assays. However, these findings should be considered exploratory and require validation in independent and larger cohorts before clinical implementation.

This study has several strengths. First, it integrates clinical and metabolomic data, capturing both physiological and biological dimensions of AHRF. Second, the use of an internally validated modeling pipeline in which feature selection was repeated within resampling reduces information leakage and limits optimism associated with high-dimensional model development. Third, the use of a temporally independent validation cohort provides an additional level of validation across different phases of the COVID-19 pandemic.

### Limitations

Several limitations should be acknowledged. The relatively small sample size, particularly in the validation cohort, limits the precision of performance estimates and reduces statistical power. The relatively modest number of patients and HFNC failure events may also result in instability of predictor selection and uncertainty in estimated performance, which cannot be eliminated by internal validation. The small validation cohort also precluded precise quantitative characterization of calibration; calibration slope and intercept and overall probabilistic prediction error should therefore be assessed in larger independent cohorts. In addition, the study was conducted in a single center and exclusively included patients with COVID-19-related AHRF, which may limit generalizability. However, the biological pathways identified, including those related to inflammation, metabolic dysregulation, and mitochondrial function, are common to AHRF of different etiologies, suggesting that the proposed approach may have broader applicability beyond COVID-19, although this requires further validation. Although temporal validation was performed, external validation in independent and more diverse cohorts is needed. Finally, the availability and standardization of metabolomic measurements may limit immediate clinical implementation.

## Conclusion

A predictive model integrating clinical and metabolomic data showed promising performance for identifying high-flow nasal cannula (HFNC) failure in patients with COVID-19-related acute hypoxemic respiratory failure. A simplified three-variable model retained promising discrimination in the validation cohort, supporting further prospective evaluation of its potential clinical utility. These findings highlight the value of combining physiological and biological information to improve risk stratification in AHRF. Further studies are required to validate these results in larger and more diverse populations and to assess their applicability beyond COVID-19.

## Data Availability

The raw data supporting the conclusions of this article will be made available by the authors, without undue reservation. Requests to access the study dataset should be directed to the corresponding author, Oscar J. Pozo, at opozo@researchmar.net.
