# Supplementary material for: Prediction of high-flow nasal cannula failure in COVID-19 acute hypoxemic respiratory failure using combined clinical and metabolomic data: a machine learning study
Source: Front Med (Lausanne). 2026 Oct 30;13:1946899. doi: 10.3389/fmed.2026.1946899 (PMC13759415; doi:10.3389/fmed.2026.1946899)
Supplement: Supplementary file 1 [file Datasheet1.docx]

**Online Data Supplement**

**Prediction of high-flow nasal cannula failure in COVID-19 acute hypoxemic respiratory failure using combined clinical and metabolomic data: a machine learning study**

Francisco José Parrilla-Gómez

Pau Nebot

Sergio Oller-Moreno

Judith Marin-Corral

Clara Vilà-Vilardell

Andrea Castellvi-Font

Purificación Pérez-Terán

Aleix Granado-García

Rosana Muñoz_Bermúdez

Llesmil Ahuir-Bauset

Cristina Soriano

Carles Camañes

Noemí Haro

Alex Gomez-Gomez

Eva Martín-López

Oscar J Pozo

Santiago Marco

Joan Ramon Masclans Enviz

**Supplemental Methods**

*Study population and data collection*

The dataset analyzed comprised 111 patients with acute hypoxemic respiratory failure (AHRF) due to COVID-19 who received high-flow nasal cannula (HFNC) therapy. Of these, 67 patients responded successfully to HFNC, while 44 experienced HFNC failure requiring intubation.

For each patient, a total of 164 variables were collected at the time of study inclusion (T0), including 25 clinical variables and 139 metabolomic measurements (111 individual metabolites and 28 metabolomic ratios). Detailed summary statistics and analyses of these variables are provided in Tables E5, E6, and E7.

After application of the complete-case criterion, the analytical dataset comprised 111 patients. No missing values remained among the 164 candidate variables used for modeling; therefore, all patients in the analytical dataset had complete predictor data and no imputation was required.

*Cohort partitioning for model development*

To develop the machine learning (ML) model and ensure robust validation, the dataset was partitioned to separate external validation samples from the model training phase. Patient admission dates (March 2020 to October 2021) were used to define temporally distinct cohorts.

April 2021, corresponding to the period with the lowest ICU admissions and the onset of the fifth COVID-19 wave in Catalonia, was selected as the cutoff. Patients admitted before this date were assigned to the Training cohort, while those admitted from April 2021 onward were reserved for the Validation cohort (Figure E1).

Feature selection and model complexity were optimized using AUC-based criteria through internal validation with bootstrap resampling (Figure E2).

The development and validation cohorts were kept strictly separated throughout the analysis. The complete model-development and internal-validation workflow was conducted within the Training Cohort: internal resampling, feature ranking and RFE, mtry and model-complexity optimization, model fitting, and internal performance assessment. The temporally independent Validation Cohort remained untouched until the final modeling procedure had been defined and was used only for final performance assessment.

*Classification method*

Given the high-dimensional structure of the dataset, combining clinical and metabolomic variables, we selected the Random Forest algorithm due to its ability to capture complex, non-linear relationships and to handle both categorical and continuous data.

Random Forest uses bootstrap sampling internally to construct individual decision trees, so that each tree is exposed to a slightly different sample of the training data. This tree-level sampling is distinct from the internal-validation resampling framework used for model development and performance estimation.

Model complexity was controlled using the area under the receiver operating characteristic curve (AUC) as the primary performance metric. The number of trees was fixed at 500, and the *mtry* parameter (number of randomly selected variables at each split) was optimized during internal validation to maximize AUC at each iteration of feature selection.

All models were implemented in R (version as reported in the main manuscript).

*Feature selection*

To identify the most relevant clinical and metabolomic predictors, we used a wrapper-based computational feature selection approach combining Random Forest variable importance with Recursive Feature Elimination (RFE) (2).

Initially, a Random Forest model including all variables was constructed. Variable importance was quantified using the mean decrease in Gini impurity, generating a ranking of features. Subsequently, the 20% least important variables were iteratively removed, and a new model was fitted using the remaining features. This process continued until no variables remained.

To ensure robustness, the entire procedure was repeated 100 times. Due to the stochastic nature of bootstrap resampling, the order in which variables were eliminated varied across iterations. As a result, each of the 100 models followed a slightly different RFE path (Figure 1, main manuscript), leading to variability in selected feature combinations.

Importantly, the complete feature-selection and model-optimization procedure was performed within the internal-validation framework. Thus, variable-importance ranking, RFE, mtry and model-complexity optimization, and model fitting were repeated using only the corresponding training data in each resampling iteration.

After implementing RFE, we aimed to determine the optimal number of variables. Models with seven variables achieved the highest median AUC (Figure 1, main manuscript). However, to balance predictive performance and model simplicity, we compared AUC distributions using the Wilcoxon test.

The difference between 7-variable and 6-variable models was not statistically significant, whereas models with fewer than six variables showed significantly lower performance. Therefore, six variables were selected as the optimal number, balancing parsimony and predictive accuracy.

Next, we identified the most frequently selected variables across the 100 six-variable models. The combination of SpO₂/FiO₂ ratio, anthranilic acid, octanoic acid, 20β-dihydrocortisone, DAG(16:1), and DAG(18:0/20:4) appeared in more than 40% of models (Figure E2) and was selected as the final feature set.

Boxplot distributions of these variables are shown in Figure E3. Significant differences between outcomes were observed for several variables, particularly SpO₂/FiO₂ ratio and anthranilic acid.

Importantly, this approach emphasizes that predictive value arises not only from individual variables but also from interactions between them, which may not be captured through univariate analyses alone.

To develop a simplified model for clinical application, we additionally analyzed the frequency of individual variables across models. The three most frequently selected variables-SpO₂/FiO₂ ratio, anthranilic acid, and octanoic acid-were used to construct the Clinical Practice model (Figure E4). In addition to selection stability, clinical feasibility was considered pragmatically according to measurement availability and timeliness, analytical robustness, potential implementation using a limited analytical platform, and the additional measurement burden required in the intended clinical setting. These considerations were used to simplify the model after the primary statistical feature-selection procedure and did not use information from the Validation Cohort.

*Model performance and statistical validation*

Model performance was primarily assessed using the AUC. As reported in the main manuscript, the final six-variable model (Best Prediction model) achieved an AUC of 0.87 (95% CI: 0.79–0.94) in internal validation and 0.91 (95% CI: 0.78–1.00) in external validation.

The three-variable Clinical Practice model achieved an AUC of 0.71 (95% CI: 0.60–0.80) in internal validation and 0.91 (95% CI: 0.77–1.00) in external validation.

To further validate these findings, permutation testing was performed on the external validation cohort (1,000 iterations). Outcome labels were randomly shuffled to generate a null distribution of AUC values expected by chance.

The observed AUC of 0.91 for the six-variable model was significantly higher than the null distribution (p < 0.001), indicating that the model’s performance was unlikely due to chance (Figure E5). The same procedure confirmed the significance of the Clinical Practice model (p < 0.001; Figure E6).

Given the limited size of the validation cohort (n=22), performance estimates are associated with considerable uncertainty. This may partially explain the higher AUC observed in external validation compared to internal validation. These uncertainties are reflected in the reported confidence intervals.

Accordingly, the observed sensitivity of 1.00 should be interpreted as a sample-specific point estimate rather than as evidence of perfect sensitivity.

A Z-test comparing AUCs (0.71 vs. 0.91) yielded statistical significance (p=0.012), although statistical power was limited (58%), indicating borderline robustness. Differences between COVID-19 waves may also have influenced model performance, although this requires further investigation.

*Adjustment for cohort differences (age distribution)*

A significant age difference was observed between cohorts (59 ± 11 years in Training vs. 50 ± 11 in Validation; p=0.021).

To address potential covariate shift, overlap weighting (OW) based on propensity scores was applied, a method particularly suited for small sample sizes. This approach achieved exact mean balance in age (standardized mean difference = 0 after weighting; Figure E7).

Model performance remained stable after adjustment. For the Best Prediction model, AUC increased from 0.87 to 0.89 in training; for the Clinical model, from 0.71 to 0.75. External validation AUC remained unchanged (0.91).

These findings suggest that differences in age distribution had limited impact on model performance.

*Model calibration*

The Hosmer–Lemeshow test was not considered appropriate for the validation cohort due to its small sample size (n=22), which results in insufficient observations per decile and violation of chi-square assumptions.

Although applicable in the training cohort (n=89), the test does not assess generalizability and may yield misleading results in overfitted models.

Therefore, calibration was assessed using calibration plots in the Validation Cohort. Visual inspection showed no obvious major discrepancy between predicted and observed probabilities; however, given the small validation cohort, the plots were interpreted descriptively rather than as definitive evidence of model calibration (Figure E8). Quantitative estimates of calibration intercept and slope, as well as overall probabilistic prediction error, should be assessed in larger independent cohorts.

*Model stability across data partitions*

To further assess robustness, models were trained using 100 random data partitions (70% training, 30% testing), ensuring balance in class distribution, sex, and age.

Results (Table E8) demonstrate consistent superiority of the six-variable model over the Clinical Practice model, and of both over the ROX index.

The distribution of AUC values across models differed significantly (p < 0.001), and all models performed significantly better than random classification, supporting the robustness of the modeling approach.

**Supplemental metabolomic data acquisition and analysis**

A total of 111 metabolites and 28 metabolic ratios were quantified using liquid chromatography–tandem mass spectrometry (LC–MS/MS).

The system consisted of an Acquity UPLC instrument (Waters Associates, Milford, MA, USA) coupled with a triple quadrupole mass spectrometer (TQS Micro, Waters). Targeted metabolites were analyzed using selected reaction monitoring (SRM). Peak integration and data processing were performed using MassLynx software (version 4.1).

**Supplemental summary of standardized High-Flow Nasal Cannula (HFNC) Management Protocol in Hospital del Mar, Barcelona**

All patients received HFNC therapy according to a standardized institutional protocol applied by the ICU team.

1. Indications for HFNC Initiation

HFNC was initiated in adult patients with acute hypoxemic respiratory failure meeting the following criteria:

Persistent hypoxemia (SpO₂/FiO₂ ≤ 200 or PaO₂/FiO₂ ≤ 200)

Respiratory rate ≥ 30 breaths/min

Signs of increased work of breathing

Need for FiO₂ ≥ 0.4 to maintain SpO₂ ≥ 92%

2. Initial HFNC Settings

HFNC was delivered using a heated humidified system with:

Initial flow: 60 L/min

Initial FiO₂: titrated to maintain SpO₂ between 92–96%

Temperature: 37°C adjusted according to patient comfort.

Flow was then adjusted based on patient comfort, respiratory effort, and oxygenation response.

3. Monitoring and Reassessment

Patients were continuously monitored with:

SpO₂ and respiratory rate

Heart rate and blood pressure

Clinical signs of respiratory distress

Arterial blood gases were obtained at clinician discretion.

Clinical reassessment was performed at least every 2–4 hours during the first 24 hours.

4. Criteria for HFNC Failure and Intubation

Intubation was considered in the presence of one or more of the following:

Persistent or worsening hypoxemia despite FiO₂ ≥ 0.8 and flow ≥ 60 L/min

Signs of respiratory muscle fatigue

Hemodynamic instability

Altered mental status

Severe acidosis (pH < 7.25)

The final decision to intubate was made by the attending intensivist according to institutional standards.

**References**

1.	Breiman L. Random forests. *Mach Learn* 2001;45:5–32.

2.	Darst BF, Malecki KC, Engelman CD. Using recursive feature elimination in random forest to account for correlated variables in high dimensional data. *BMC Genet* 2018;19:65.

**Tables**

**Table E1:** Patients demographic variables at study inclusion (T0).

A. Training Cohort

|  | **ALL**  (n = 89) | **SUCCESS**  (n = 54) | **FAILURE**  (n = 35) | **p-value*** |
| --- | --- | --- | --- | --- |
| Age (years) | 59 ± 12 | 56 ± 12 | 62 ± 9 | 0.330 |
| Gender (female), n (%) | 30 (33.71) | 20 (37.04) | 10 (28.57) | 0.660 |
| Obesity, n (%) | 13 (14.61) | 5 (9.26) | 8 (22.86) | 0.280 |
| Diabetes Mellitus, n (%) | 17 (19.1) | 7 (12.96) | 10 (28.57) | 0.280 |
| Dyslipidemia, n (%) | 26 (29.21) | 14 (25.93) | 12 (34.29) | 0.660 |
| Arterial hypertension, n (%) | 43 (48.31) | 23 (42.59) | 20 (57.14) | 0.390 |
| BMI (Kg/m²) | 28 ± 5 | 28 ± 5 | 28 ± 6 | 0.920 |
| Symptom days before day 1 | 10 ± 6 | 11 ± 6 | 9 ± 4 | 0.460 |
| Number of comorbidities | 1.37 ± 1.34 | 1.3 ± 1.38 | 1.49 ± 1.29 | 0.720 |
| APACHE II (Score) | 14 ± 6 | 13 ± 6 | 17 ± 5 | **0.022** |
| Norepinephrine (>0.1 μg/kg/min), n (%) | 40 (44.94) | 5 (9.26) | 35 (100) | **< 0.001** |

*Values reflect p-values adjusted using the Benjamini & Hochberg method.

BMI: Body Mass Index, APACHE II: Acute Physiology and Chronic Health Evaluation.

B. Validation Cohort

|  | **ALL**  (n = 22) | **SUCCESS**  (n = 13) | **FAILURE**  (n = 9) | **p-value*** |
| --- | --- | --- | --- | --- |
| Age (years) | 50 ± 11 | 52 ± 10 | 47 ± 11 | 0.810 |
| Gender (female), n (%) | 9 (40.91) | 7 (53.85) | 2 (22.22) | 0.990 |
| Obesity, n (%) | 5 (22.73) | 4 (30.77) | 1 (11.11) | 1.000 |
| Diabetes Mellitus, n (%) | 4 (18.18) | 3 (23.08) | 1 (11.11) | 1.000 |
| Dyslipidemia, n (%) | 3 (13.64) | 1 (7.69) | 2 (22.22) | 1.000 |
| Arterial hypertension, n (%) | 4 (18.18) | 2 (15.38) | 2 (22.22) | 1.000 |
| BMI (Kg/m²) | 29 ± 9 | 28 ± 5 | 31 ± 14 | 1.000 |
| Symptom days before day 1 | 11 ± 9 | 9 ± 3 | 15 ± 14 | 0.830 |
| Number of comorbidities | 1.14 ± 1.21 | 0.62 ± 0.77 | 1.89 ± 1.36 | 0.310 |
| APACHE II (Score) | 14 ± 6 | 14 ± 7 | 14 ± 6 | 1.000 |
| Norepinephrine (>0.1 μg/kg/min), n (%) | 9 (40.91) | 1 (7.69) | 8 (88.89) | **0.008** |

*Values reflect p-values adjusted using the Benjamini & Hochberg method.

BMI: Body Mass Index, APACHE II: Acute Physiology and Chronic Health Evaluation.

C. Training and Validation Cohorts

|  | **ALL**  (n = 111) | **SUCCESS**  (n = 67) | **FAILURE**  (n = 44) | **p-value*** |
| --- | --- | --- | --- | --- |
| Age (years) | 57 ± 12 | 56 ± 12 | 59 ± 11 | 0.450 |
| Gender (female), n (%) | 39 (35.14) | 27 (40.30) | 12 (27.27) | 0.410 |
| Obesity, n (%) | 18 (16.22) | 9 (13.43) | 9 (20.45) | 0.560 |
| Diabetes Mellitus, n (%) | 21 (18.92) | 10 (14.93) | 11 (25.00) | 0.410 |
| Dyslipidemia, n (%) | 29 (26.13) | 15 (22.39) | 14 (31.82) | 0.490 |
| Arterial hypertension, n (%) | 47 (42.34) | 25 (37.31) | 22 (50.00) | 0.410 |
| BMI (Kg/m²) | 28 ± 6 | 28 ± 5 | 29 ± 8 | 0.800 |
| Symptom days before day 1 | 11 ± 7 | 11 ± 6 | 10 ± 8 | 0.720 |
| Number of comorbidities | 1.32 ± 1.32 | 1.16 ± 1.31 | 1.57 ± 1.30 | 0.340 |
| APACHE II (Score) | 14 ± 6 | 13 ± 6 | 16 ± 5 | **0.035** |
| Norepinephrine (>0.1 μg/kg/min), n (%) | 49 (44.14) | 6 (8.96) | 43 (97.73) | **< 0.001** |

*Values reflect p-values adjusted using the Benjamini & Hochberg method.

BMI: Body Mass Index, APACHE II: Acute Physiology and Chronic Health Evaluation.

**Table E2:** Clinical characteristics at study inclusion (T0)

**A.** Training Cohort

|  | **ALL**  (n = 89) | **SUCCESS**  (n = 54) | **FAILURE**  (n = 35) | **p-value*** |
| --- | --- | --- | --- | --- |
| **Respiratory condition and vital signs** |  |  |  |  |
| Heart rate (bpm) | 77 ± 16 | 75 ± 15 | 80 ± 18 | 0.850 |
| MAP | 91 ± 17 | 92 ± 16 | 91 ± 18 | 0.940 |
| Respiratory rate (RR) | 23 ± 8 | 23 ± 7 | 24 ± 8 | 0.940 |
| SpO₂ | 94 ± 4 | 95 ± 3 | 93 ± 4 | **0.031** |
| HFNC L/min | 59 ± 4 | 58 ± 5 | 60 ± 0 | 0.340 |
| HFNC FᵢO₂ | 65 ± 13 | 61 ± 14 | 70 ± 9 | 0.091 |
| SatO₂/FᵢO₂ | 153 ± 40 | 165 ± 45 | 134 ± 19 | **0.022** |
| ROX INDEX | 7 ± 4 | 8 ± 4 | 6 ± 3 | 0.330 |
| **Blood arterial gases** |  |  |  |  |
| pH | 7.43 ± 0.06 | 7.43 ± 0.03 | 7.42 ± 0.09 | 0.960 |
| PaCO₂ (mmHg) | 34.18 ± 6.35 | 34.84 ± 6.58 | 32.79 ± 5.77 | 0.620 |
| PaO₂ (mmHg) | 89.14 ± 50.21 | 91.98 ± 57.18 | 83.17 ± 31.38 | 1.000 |
| HCO₃- (mmol/L) | 23.48 ± 3.51 | 23.78 ± 2.82 | 22.84 ± 4.67 | 0.940 |
| Base excess (mmol/L) | -1.9 ± 5.19 | -1.44 ± 4.23 | -2.86 ± 6.81 | 0.940 |
| SatO₂ (%) | 93.75 ± 6.47 | 93.69 ± 6.6 | 93.88 ± 6.38 | 0.990 |
| PaO₂/FiO₂ | 148.02 ± 112.75 | 160.72 ± 130.89 | 120.5 ± 49.05 | 0.450 |
| Lactate Plasma (mmoL/L) | 1.68 ± 0.65 | 1.65 ± 0.56 | 1.72 ± 0.83 | 0.940 |
| **Timing delays** |  |  |  |  |
| Symptoms start to ICU admission (days) | 10 (7 - 11) | 10 (8 - 12) | 9 (6 - 11) | 0.500 |
| Hospital admission to ICU admission (days) | 3 (1 - 6) | 3 (1 - 5) | 2 (1 - 6) | 0.840 |
| Hospital admission to T0 (days) | 3 (1 - 6) | 4 (1 - 5) | 2 (1 - 6) | 0.750 |
| **Medication at inclusion** |  |  |  |  |
| Vaccination status (complete), n (%) | 0 (0) | 0 (0) | 0 (0) |  |
| Remdesivir, n (%) | 15 (20.83) | 12 (26.09) | 3 (11.54) | 0.390 |
| Tocilizumab, n (%) | 25 (34.72) | 17 (36.96) | 8 (30.77) | 0.860 |
| Hydroxychloroquine, n (%) | 13 (18.06) | 9 (19.57) | 4 (15.38) | 0.900 |
| Dexamethasone, n (%) | 62 (86.11) | 37 (80.43) | 25 (96.15) | 0.280 |

*Values reflect p-values adjusted using the Benjamini & Hochberg method.

MAP: Mean Arterial Pressure, RR: Respiratory Rate, SpO₂: Saturation of peripheral oxygen, HFNC: High-flow nasal cannula, FᵢO₂: Fraction of Inspired Oxygen, SatO₂: Peripheral oxygen saturation, ROX: Ratio of Oxygen Saturation, PaCO₂: Carbon dioxide partial pressure in arterial blood gas sample, PaO₂: Oxygen partial pressure in arterial blood gas sample, HCO₃-: Bicarbonate.

**B.** Validation Cohort

|  | **ALL**  (n = 22) | **SUCCESS**  (n = 13) | **FAILURE**  (n = 9) | **p-value*** |
| --- | --- | --- | --- | --- |
| **Respiratory condition and vital signs** |  |  |  |  |
| Heart rate (bpm) | 71 ± 15 | 66 ± 11 | 77 ± 17 | 0.720 |
| MAP | 89 ± 12 | 89 ± 12 | 90 ± 12 | 1.000 |
| Respiratory rate (RR) | 25 ± 8 | 27 ± 7 | 22 ± 9 | 0.810 |
| SpO₂ | 94 ± 3 | 94 ± 3 | 95 ± 2 | 1.000 |
| HFNC L/min | 55 ± 13 | 55 ± 15 | 54 ± 10 | 0.760 |
| HFNC FᵢO₂ | 66 ± 16 | 62 ± 13 | 72 ± 19 | 0.480 |
| SatO₂/FᵢO₂ | 152 ± 43 | 158 ± 37 | 143 ± 51 | 0.480 |
| ROX INDEX | 7 ± 3 | 6 ± 3 | 8 ± 4 | 1.000 |
| **Blood arterial gases** |  |  |  |  |
| pH | 7.47 ± 0.03 | 7.47 ± 0.03 | 7.48 ± 0.04 | 0.950 |
| PaCO₂ (mmHg) | 33.35 ± 3.7 | 34.31 ± 3.34 | 31.44 ± 4.01 | 0.730 |
| PaO₂ (mmHg) | 68.24 ± 25.38 | 67.47 ± 20.16 | 69.78 ± 36.55 | 1.000 |
| HCO₃- (mmol/L) | 24.83 ± 1.8 | 25.22 ± 1.98 | 24.06 ± 1.21 | 0.730 |
| Base excess (mmol/L) | -0.06 ± 2.27 | 0.4 ± 2.52 | -0.98 ± 1.49 | 0.760 |
| SatO₂ (%) | 91.19 ± 9.1 | 92.92 ± 3.88 | 87.72 ± 15.28 | 1.000 |
| PaO₂/FiO₂ | 111.42 ± 46.69 | 114.49 ± 43.55 | 105.27 ± 57.37 | 0.480 |
| Lactate Plasma (mmoL/L) | 1.61 ± 0.57 | 1.49 ± 0.39 | 1.88 ± 0.86 | 0.980 |
| **Timing delays** |  |  |  |  |
| Symptoms start to ICU admission (days) | 9 (7 - 13) | 8 (7 - 9) | 11 (7 - 14) | 0.830 |
| Hospital admission to ICU admission (days) | 2 (1 - 4) | 2 (1 - 3) | 4 (2 - 6) | 0.730 |
| Hospital admission to D1 (days) | 2 (1 - 4) | 2 (1 - 3) | 4 (2 - 6) | 0.730 |
| **Medications at inclusion** |  |  |  |  |
| Vaccination status (complete), n (%) | 1 (4.55) | 0 (0) | 1 (11.11) | 1.000 |
| Remdesivir, n (%) | 0 (0) | 0 (0) | 0 (0) |  |
| Tocilizumab, n (%) | 4 (22.22) | 2 (18.18) | 2 (28.57) | 1.000 |
| Hydroxychloroquine, n (%) | 0 (0) | 0 (0) | 0 (0) |  |
| Dexamethasone, n (%) | 18 (100) | 11 (100) | 7 (100) |  |

*Values reflect p-values adjusted using the Benjamini & Hochberg method.

MAP: Mean Arterial Pressure, RR: Respiratory Rate, SpO₂: Saturation of peripheral oxygen, HFNC: High-flow nasal cannula, FᵢO₂: Fraction of Inspired Oxygen, SatO₂: Peripheral oxygen saturation, ROX: Ratio of Oxygen Saturation, PaCO₂: Carbon dioxide partial pressure in arterial blood gas sample, PaO₂: Oxygen partial pressure in arterial blood gas sample, HCO₃-: Bicarbonate

**C.** Training and Validation Cohorts

|  | **ALL**  (n = 111) | **SUCCESS**  (n = 67) | **FAILURE**  (n = 44) | **p-value*** |
| --- | --- | --- | --- | --- |
| **Respiratory condition and vital signs** |  |  |  |  |
| Heart rate (bpm) | 76 ± 16 | 74 ± 15 | 79 ± 18 | 0.580 |
| MAP | 91 ± 16 | 91 ± 16 | 91 ± 16 | 0.970 |
| Respiratory rate (RR) | 24 ± 8 | 24 ± 8 | 23 ± 8 | 0.980 |
| SpO₂ | 94 ± 3 | 95 ± 3 | 93 ± 4 | 0.070 |
| HFNC L/min | 58 ± 7 | 58 ± 8 | 59 ± 5 | 0.760 |
| HFNC FᵢO₂ | 0.65 ± 0.14 | 0.62 ± 0.14 | 0.71 ± 0.12 | **0.016** |
| SatO₂/FᵢO₂ | 153 ± 40 | 164 ± 43 | 136 ± 28 | **< 0.001** |
| ROX INDEX | 7.37 ± 3.83 | 7.82 ± 4.13 | 6.69 ± 3.26 | 0.420 |
| **Blood arterial gases** |  |  |  |  |
| pH | 7.44 ± 0.06 | 7.44 ± 0.04 | 7.43 ± 0.08 | 0.970 |
| PaCO₂ (mmHg) | 34.02 ± 5.9 | 34.74 ± 6.04 | 32.51 ± 5.4 | 0.400 |
| PaO₂ (mmHg) | 84.91 ± 46.88 | 87.08 ± 52.68 | 80.38 ± 32.15 | 1.000 |
| HCO₃- (mmol/L) | 23.75 ± 3.27 | 24.07 ± 2.72 | 23.1 ± 4.19 | 0.800 |
| Base excess (mmol/L) | -1.52 ± 4.79 | -1.07 ± 4 | -2.47 ± 6.11 | 0.820 |
| SatO₂ (%) | 93.23 ± 7.09 | 93.54 ± 6.12 | 92.6 ± 8.89 | 1.000 |
| PaO₂/FiO₂ | 140.39 ± 103.35 | 151.28 ± 119.47 | 117.19 ± 49.99 | 0.360 |
| Lactate Plasma (mmoL/L) | 1.66 ± 0.63 | 1.62 ± 0.53 | 1.75 ± 0.82 | 1.000 |
| **Timing delays** |  |  |  |  |
| Symptoms start to ICU admission (days) | 9 (7 - 13) | 8 (7 - 9) | 11 (7 - 14) | 0.740 |
| Hospital admission to ICU admission (days) | 2 (1 - 4) | 2 (1 - 3) | 4 (2 - 6) | 0.990 |
| Hospital admission to D1 (days) | 3 (1 - 4) | 2 (1 - 3) | 4 (2 - 6) | 0.930 |
| **Medications at inclusion** |  |  |  |  |
| Vaccination status (complete), n (%) | 1 (0.90) | 0 (0.00) | 1 (2.27) | 0.410 |
| Remdesivir, n (%) | 15 (16.67) | 12 (21.05) | 3 (9.09) | 0.410 |
| Tocilizumab, n (%) | 29 (32.22) | 19 (33.33) | 10 (30.30) | 0.950 |
| Hydroxychloroquine, n (%) | 13 (14.44) | 9 (15.79) | 4 (12.12) | 0.940 |
| Dexamethasone, n (%) | 80 (88.89) | 48 (84.21) | 32 (96.97) | 0.410 |

*Values reflect p-values adjusted using the Benjamini & Hochberg method.

MAP: Mean Arterial Pressure, RR: Respiratory Rate, SpO₂: Saturation of peripheral oxygen, HFNC: High-flow nasal cannula, FᵢO₂: Fraction of Inspired Oxygen, SatO₂: Peripheral oxygen saturation, ROX: Ratio of Oxygen Saturation, PaCO₂: Carbon dioxide partial pressure in arterial blood gas sample, PaO₂: Oxygen partial pressure in arterial blood gas sample, HCO₃-: Bicarbonate.

**Table E3:** Laboratory results at T0.

**A.** Training vs. Validation Cohorts

|  | **ALL**  (n = 111) | **TRAINING**  (n = 89) | **VALIDATION**  (n = 22) | **p-value*** |
| --- | --- | --- | --- | --- |
| **Plasma Biochemistry** |  |  |  |  |
| Glucose (mg/dL) | 150.84 ± 69.73 | 149.91 ± 73.28 | 154.63 ± 54.46 | 0.840 |
| Urea (mg/dl) | 43.62 ± 22.72 | 43.94 ± 24.37 | 42.29 ± 14.47 | 0.950 |
| Creatinine (mg/dl) | 0.96 ± 0.92 | 1 ± 1.02 | 0.78 ± 0.22 | 0.960 |
| GFT (mL/min) | 90.63 ± 24.63 | 88.37 ± 26.1 | 99.68 ± 14.92 | 0.430 |
| Sodium (mmol/L) | 137.96 ± 4.29 | 138.13 ± 4.49 | 137.26 ± 3.38 | 0.720 |
| Potassium (mmol/L) | 4.27 ± 0.48 | 4.27 ± 0.5 | 4.29 ± 0.42 | 1.000 |
| Calcium (mg/dL) | 8.46 ± 0.71 | 8.46 ± 0.76 | 8.45 ± 0.42 | 0.960 |
| Ionized calcium measured (mmol/L) | 1.13 ± 0.07 | 1.12 ± 0.07 | 1.13 ± 0.05 | 0.890 |
| Magnesium (mg/dL) | 2.24 ± 0.25 | 2.24 ± 0.24 | 2.27 ± 0.28 | 0.970 |
| Phosphate (mg/dL) | 3.16 ± 0.88 | 3.2 ± 0.88 | 3 ± 0.92 | 0.890 |
| LDH (U/L) | 448.56 ± 203.15 | 423.79 ± 157.35 | 544.89 ± 313.6 | 0.680 |
| Creatine phosphokinase (u/l) | 221.9 ± 525.88 | 199.54 ± 550.59 | 303.11 ± 427.24 | 0.840 |
| Total bilirubin (mg/dL) | 0.5 ± 0.33 | 0.48 ± 0.31 | 0.56 ± 0.43 | 0.690 |
| AST (GOT) (U/L) | 67.78 ± 87.04 | 66.07 ± 92.78 | 74.83 ± 59.32 | 0.410 |
| ALT (GPT) (U/L) | 72.49 ± 102.95 | 70.41 ± 109.85 | 80.84 ± 70.56 | 0.430 |
| Alkaline phosphatase (U/L) | 89.24 ± 68.67 | 88.59 ± 71.64 | 92 ± 56.09 | 1.000 |
| Proteins totals (g/dL) | 6.36 ± 0.67 | 6.37 ± 0.68 | 6.34 ± 0.64 | 0.980 |
| Albumin (g/dL) | 3.5 ± 0.47 | 3.47 ± 0.47 | 3.64 ± 0.49 | 0.480 |
| Procalcitonin (ng/ml) | 3.75 ± 15.45 | 4.74 ± 17.35 | 0.17 ± 0.22 | 0.170 |
| C-reactive protein (mg/dl) | 10.63 ± 8.97 | 11.68 ± 9.33 | 6.45 ± 5.83 | 0.160 |
| Troponin I (ng/l) | 21.9 ± 33.27 | 23.87 ± 37.07 | 14.18 ± 0.68 | 0.400 |
| Ferritin (ng/ml) | 2160.21 ± 2633.15 | 2171.98 ± 2639.9 | 2103.7 ± 2741.13 | 0.850 |
| Interleukin 6 (pg/ml) | 147.65 ± 402.73 | 105.29 ± 119.7 | 301.2 ± 838.33 | 0.870 |
| **Hemogram** |  |  |  |  |
| Hemoglobin (g/dL) | 12.98 ± 1.83 | 12.9 ± 1.9 | 13.33 ± 1.55 | 0.790 |
| Hematocrits (%) | 39.74 ± 5.38 | 39.31 ± 5.46 | 41.46 ± 4.79 | 0.480 |
| Leukocytes (10³u/μl) | 9.44 ± 4.56 | 9.55 ± 4.86 | 8.98 ± 3.16 | 0.980 |
| Neutrophils T (10³u/μl) | 8.09 ± 4.34 | 8.22 ± 4.61 | 7.54 ± 3.11 | 1.000 |
| Lymphocytes T (10³u/μl) | 0.86 ± 0.44 | 0.85 ± 0.47 | 0.9 ± 0.34 | 0.720 |
| Monocytes T (10³u/μl) | 0.45 ± 0.31 | 0.44 ± 0.32 | 0.49 ± 0.29 | 0.720 |
| Eosinophils T (10³u/μl) | 0.03 ± 0.06 | 0.02 ± 0.06 | 0.03 ± 0.06 | 0.930 |
| Basophilos T (10³u/μl) | 0.01 ± 0.01 | 0.01 ± 0.01 | 0.02 ± 0.01 | 0.680 |
| Plateles (10³u/μl) | 266.81 ± 112.85 | 268.44 ± 104.37 | 260.21 ± 145.49 | 0.720 |
| **Coagulation** |  |  |  |  |
| T. prothrombin (activity) (%) | 101.27 ± 19.65 | 98.23 ± 18.56 | 114.11 ± 19.44 | **0.040** |
| T. prothrombin (seconds) | 10.92 ± 0.86 | 11.01 ± 0.89 | 10.54 ± 0.6 | 0.220 |
| T. prothrombin (ratio) | 1.03 ± 0.08 | 1.03 ± 0.08 | 1 ± 0.06 | 0.310 |
| INR Plasma | 1.04 ± 0.09 | 1.05 ± 0.09 | 1 ± 0.06 | 0.270 |
| APTT (seconds) | 28.8 ± 4.88 | 29.22 ± 5.13 | 27.09 ± 3.28 | 0.380 |
| APTT (ratio) | 1.08 ± 0.18 | 1.09 ± 0.19 | 1.04 ± 0.13 | 0.790 |
| D-Dimer (μg/l) | 2918.4 ± 6946.69 | 2474.08 ± 6247.12 | 4794.44 ± 9338.44 | 1.000 |

*Values reflect p-values adjusted using the Benjamini & Hochberg method.

GFT: Glomerular Filtration Rate, LDH: Lactate dehydrogenase, AST: Aspartate aminotransferase, ALT: Alanine aminotransferase, INR: International normalized ratio, APTT: activated partial thromboplastin time.

**B.** Training Cohort

|  | **ALL**  (n = 89) | **SUCCESS**  (n = 54) | **FAILURE**  (n = 35) | **p-value*** |
| --- | --- | --- | --- | --- |
| **Plasma Biochemistry** |  |  |  |  |
| Glucose (mg/dL) | 149.91 ± 73.28 | 142.94 ± 52.42 | 164.4 ± 104.13 | 0.850 |
| Urea (mg/dl) | 43.94 ± 24.37 | 38.83 ± 14.45 | 54.79 ± 35.68 | 0.500 |
| Creatinine (mg/dl) | 1 ± 1.02 | 0.85 ± 0.77 | 1.33 ± 1.37 | 0.280 |
| GFT (mL/min) | 88.37 ± 26.1 | 93.43 ± 22 | 78.04 ± 30.9 | 0.130 |
| Sodium (mmol/L) | 138.13 ± 4.49 | 138.85 ± 3.64 | 136.6 ± 5.68 | 0.420 |
| Potassium (mmol/L) | 4.27 ± 0.5 | 4.26 ± 0.5 | 4.3 ± 0.53 | 0.940 |
| Calcium (mg/dL) | 8.46 ± 0.76 | 8.57 ± 0.55 | 8.22 ± 1.06 | 0.760 |
| Ionized calcium measured (mmol/L) | 1.12 ± 0.07 | 1.13 ± 0.07 | 1.11 ± 0.09 | 0.860 |
| Magnesium (mg/dL) | 2.24 ± 0.24 | 2.26 ± 0.25 | 2.18 ± 0.23 | 0.850 |
| Phosphate (mg/dL) | 3.2 ± 0.88 | 3.23 ± 0.9 | 3.12 ± 0.87 | 0.940 |
| LDH (U/L) | 423.79 ± 157.35 | 399.68 ± 130.42 | 473.04 ± 195.74 | 0.700 |
| Creatine phosphokinase (u/l) | 199.54 ± 550.59 | 142.55 ± 165.98 | 299.84 ± 890.58 | 0.960 |
| Total bilirubin (mg/dL) | 0.48 ± 0.31 | 0.42 ± 0.22 | 0.61 ± 0.41 | 0.450 |
| AST (GOT) (U/L) | 66.07 ± 92.78 | 58.68 ± 69.32 | 81.46 ± 129.3 | 0.850 |
| ALT (GPT) (U/L) | 70.41 ± 109.85 | 66.55 ± 86.66 | 78.28 ± 148.21 | 0.980 |
| Alkaline phosphatase (U/L) | 88.59 ± 71.64 | 82.57 ± 45.71 | 100.88 ± 107.01 | 0.960 |
| Proteins totals (g/dL) | 6.37 ± 0.68 | 6.47 ± 0.7 | 6.07 ± 0.52 | 0.460 |
| Albumin (g/dL) | 3.47 ± 0.47 | 3.51 ± 0.49 | 3.36 ± 0.37 | 0.700 |
| Procalcitonin (ng/ml) | 4.74 ± 17.35 | 5.16 ± 20.23 | 3.98 ± 10.68 | 0.940 |
| C-reactive protein (mg/dl) | 11.68 ± 9.33 | 10.42 ± 9.97 | 14.24 ± 7.42 | 0.280 |
| Troponin I (ng/l) | 23.87 ± 37.07 | 17.87 ± 13.37 | 34.73 ± 58.83 | 0.940 |
| Ferritin (ng/ml) | 2171.98 ± 2639.9 | 1924.45 ± 2460.99 | 2716.53 ± 3015.06 | 0.700 |
| Interleukin 6 (pg/ml) | 105.29 ± 119.7 | 105.08 ± 140.73 | 105.76 ± 50.99 | 0.570 |
| **Hemogram** |  |  |  |  |
| Hemoglobin (g/dL) | 12.9 ± 1.9 | 12.87 ± 1.85 | 12.95 ± 2.03 | 1.000 |
| Hematocrits (%) | 39.31 ± 5.46 | 39.39 ± 5.07 | 39.16 ± 6.29 | 0.940 |
| Leukocytes (10³u/μl) | 9.55 ± 4.86 | 9.19 ± 4.37 | 10.29 ± 5.77 | 0.850 |
| Neutrophils T (10³u/μl) | 8.22 ± 4.61 | 7.84 ± 4.23 | 9.01 ± 5.32 | 0.830 |
| Lymphocytes T (10³u/μl) | 0.85 ± 0.47 | 0.89 ± 0.5 | 0.78 ± 0.39 | 0.810 |
| Monocytes T (10³u/μl) | 0.44 ± 0.32 | 0.42 ± 0.28 | 0.47 ± 0.4 | 1.000 |
| Eosinophils T (10³u/μl) | 0.02 ± 0.06 | 0.03 ± 0.06 | 0.02 ± 0.05 | 0.980 |
| Basophilos T (10³u/μl) | 0.01 ± 0.01 | 0.01 ± 0.01 | 0.01 ± 0.01 | 0.940 |
| Plateles (10³u/μl) | 268.44 ± 104.37 | 270 ± 100.2 | 265.2 ± 114.64 | 0.940 |
| **Coagulation** |  |  |  |  |
| T. prothrombin (activity) (%) | 98.23 ± 18.56 | 98.39 ± 17.17 | 97.91 ± 21.49 | 0.960 |
| T. prothrombin (seconds) | 11.01 ± 0.89 | 11 ± 0.72 | 11.03 ± 1.18 | 0.700 |
| T. prothrombin (ratio) | 1.03 ± 0.08 | 1.03 ± 0.06 | 1.04 ± 0.11 | 0.850 |
| INR Plasma | 1.05 ± 0.09 | 1.04 ± 0.07 | 1.06 ± 0.13 | 0.960 |
| APTT (seconds) | 29.22 ± 5.13 | 29.41 ± 5.74 | 28.82 ± 3.59 | 1.000 |
| APTT (ratio) | 1.09 ± 0.19 | 1.1 ± 0.21 | 1.06 ± 0.14 | 0.850 |
| D-Dimer (μg/l) | 2474.08 ± 6247.12 | 2216.15 ± 6891.26 | 3032.92 ± 4634.76 | 0.500 |

*Values reflect p-values adjusted using the Benjamini & Hochberg method.

GFT: Glomerular Filtration Rate, LDH: Lactate dehydrogenase, AST: Aspartate aminotransferase, ALT: Alanine aminotransferase, INR: International normalized ratio, APTT: activated partial thromboplastin time.

**C.** Validation Cohort

|  | **ALL**  (n = 22) | **SUCCESS**  (n = 13) | **FAILURE**  (n = 9) | **p-value*** |
| --- | --- | --- | --- | --- |
| **Plasma Biochemistry** |  |  |  |  |
| Glucose (mg/dL) | 154.63 ± 54.46 | 152.31 ± 49.33 | 159.67 ± 69.23 | 1.000 |
| Urea (mg/dl) | 42.29 ± 14.47 | 38.64 ± 11.48 | 50.2 ± 18.11 | 0.720 |
| Creatinine (mg/dl) | 0.78 ± 0.22 | 0.79 ± 0.24 | 0.74 ± 0.15 | 1.000 |
| GFT (mL/min) | 99.68 ± 14.92 | 95.69 ± 14.57 | 108.33 ± 12.68 | 0.610 |
| Sodium (mmol/L) | 137.26 ± 3.38 | 138.08 ± 3.45 | 135.5 ± 2.66 | 0.640 |
| Potassium (mmol/L) | 4.29 ± 0.42 | 4.33 ± 0.3 | 4.2 ± 0.63 | 0.940 |
| Calcium (mg/dL) | 8.45 ± 0.42 | 8.5 ± 0.46 | 8.38 ± 0.36 | 0.900 |
| Ionized calcium measured (mmol/L) | 1.13 ± 0.05 | 1.12 ± 0.03 | 1.14 ± 0.07 | 1.000 |
| Magnesium (mg/dL) | 2.27 ± 0.28 | 2.2 ± 0.25 | 2.36 ± 0.34 | 0.980 |
| Phosphate (mg/dL) | 3 ± 0.92 | 3.22 ± 0.46 | 2.7 ± 1.41 | 0.980 |
| LDH (U/L) | 544.89 ± 313.6 | 432.08 ± 188.76 | 770.5 ± 405.42 | 0.480 |
| Creatine phosphokinase (u/l) | 303.11 ± 427.24 | 363.85 ± 484.73 | 171.5 ± 250.66 | 0.950 |
| Total bilirubin (mg/dL) | 0.56 ± 0.43 | 0.46 ± 0.24 | 0.78 ± 0.67 | 0.760 |
| AST (GOT) (U/L) | 74.83 ± 59.32 | 92.67 ± 65.35 | 39.17 ± 16.7 | 0.480 |
| ALT (GPT) (U/L) | 80.84 ± 70.56 | 94.54 ± 79.49 | 51.17 ± 34.9 | 0.740 |
| Alkaline phosphatase (U/L) | 92 ± 56.09 | 81.67 ± 50.44 | 112.67 ± 65.81 | 0.760 |
| Proteins totals (g/dL) | 6.34 ± 0.64 | 6.46 ± 0.68 | 6.12 ± 0.61 | 0.870 |
| Albumin (g/dL) | 3.64 ± 0.49 | 3.7 ± 0.53 | 3.52 ± 0.45 | 0.870 |
| Procalcitonin (ng/ml) | 0.17 ± 0.22 | 0.17 ± 0.26 | 0.16 ± 0.12 | 1.000 |
| C-reactive protein (mg/dl) | 6.45 ± 5.83 | 6.79 ± 5.48 | 5.73 ± 7.04 | 0.900 |
| Troponin I (ng/l) | 14.18 ± 0.68 | 14 ± 0 | 14.53 ± 1.18 | 0.730 |
| Ferritin (ng/ml) | 2103.7 ± 2741.13 | 2214.17 ± 3452.8 | 1938 ± 1615.76 | 1.000 |
| Interleukin 6 (pg/ml) | 301.2 ± 838.33 | 39.53 ± 25.79 | 876.88 ± 1425.22 | 0.480 |
| **Hemogram** |  |  |  |  |
| Hemoglobin (g/dL) | 13.33 ± 1.55 | 13.42 ± 1.19 | 13.12 ± 2.27 | 1.000 |
| Hematocrits (%) | 41.46 ± 4.79 | 41.8 ± 3.28 | 40.73 ± 7.46 | 1.000 |
| Leukocytes (10³u/μl) | 8.98 ± 3.16 | 8.81 ± 2.56 | 9.35 ± 4.46 | 0.980 |
| Neutrophils T (10³u/μl) | 7.54 ± 3.11 | 7.3 ± 2.46 | 8.06 ± 4.45 | 0.960 |
| Lymphocytes T (10³u/μl) | 0.9 ± 0.34 | 1 ± 0.36 | 0.7 ± 0.2 | 0.340 |
| Monocytes T (10³u/μl) | 0.49 ± 0.29 | 0.47 ± 0.24 | 0.53 ± 0.4 | 1.000 |
| Eosinophils T (10³u/μl) | 0.03 ± 0.06 | 0.03 ± 0.07 | 0.03 ± 0.06 | 0.980 |
| Basophilos T (10³u/μl) | 0.02 ± 0.01 | 0.01 ± 0.02 | 0.02 ± 0.01 | 0.280 |
| Plateles (10³u/μl) | 260.21 ± 145.49 | 253.23 ± 129.62 | 275.33 ± 188.36 | 1.000 |
| **Coagulation** |  |  |  |  |
| T. prothrombin (activity) (%) | 114.11 ± 19.44 | 122.74 ± 17.2 | 96.85 ± 9.93 | 0.072 |
| T. prothrombin (seconds) | 10.54 ± 0.6 | 10.24 ± 0.44 | 11.15 ± 0.36 | 0.072 |
| T. prothrombin (ratio) | 1 ± 0.06 | 0.97 ± 0.04 | 1.05 ± 0.03 | 0.072 |
| INR Plasma | 1 ± 0.06 | 0.97 ± 0.03 | 1.04 ± 0.05 | 0.210 |
| APTT (seconds) | 27.09 ± 3.28 | 27.45 ± 3.31 | 26.38 ± 3.4 | 1.000 |
| APTT (ratio) | 1.04 ± 0.13 | 1.06 ± 0.13 | 1.01 ± 0.13 | 1.000 |
| D-Dimer (μg/l) | 4794.44 ± 9338.44 | 1323.33 ± 2263.4 | 11736.67 ± 14088.35 | 0.470 |

*Values reflect p-values adjusted using the Benjamini & Hochberg method.

GFT: Glomerular Filtration Rate, LDH: Lactate dehydrogenase, AST: Aspartate aminotransferase, ALT: Alanine aminotransferase, INR: International normalized ratio, APTT: activated partial thromboplastin time.

**D.** Training and Validation Cohorts

|  | **ALL**  (n = 111) | **SUCCESS**  (n = 67) | **FAILURE**  (n = 44) | **p-value*** |
| --- | --- | --- | --- | --- |
| **Plasma Biochemistry** |  |  |  |  |
| Glucose (mg/dL) | 150.84 ± 69.73 | 144.82 ± 51.58 | 163.48 ± 97.35 | 0.790 |
| Urea (mg/dl) | 43.62 ± 22.72 | 38.79 ± 13.83 | 53.9 ± 32.81 | 0.320 |
| Creatinine (mg/dl) | 0.96 ± 0.92 | 0.84 ± 0.69 | 1.21 ± 1.25 | 0.320 |
| GFT (mL/min) | 90.63 ± 24.63 | 93.89 ± 20.62 | 83.9 ± 30.64 | 0.490 |
| Sodium (mmol/L) | 137.96 ± 4.29 | 138.7 ± 3.59 | 136.39 ± 5.21 | 0.170 |
| Potassium (mmol/L) | 4.27 ± 0.48 | 4.27 ± 0.46 | 4.28 ± 0.54 | 0.790 |
| Calcium (mg/dL) | 8.46 ± 0.71 | 8.56 ± 0.53 | 8.26 ± 0.95 | 0.630 |
| Ionized calcium measured (mmol/L) | 1.13 ± 0.07 | 1.13 ± 0.06 | 1.12 ± 0.08 | 0.930 |
| Magnesium (mg/dL) | 2.24 ± 0.25 | 2.25 ± 0.24 | 2.23 ± 0.26 | 0.980 |
| Phosphate (mg/dL) | 3.16 ± 0.88 | 3.23 ± 0.84 | 3.02 ± 0.98 | 0.790 |
| LDH (U/L) | 448.56 ± 203.15 | 406.27 ± 142.9 | 534.59 ± 272.93 | 0.350 |
| Creatine phosphokinase (u/l) | 221.9 ± 525.88 | 193.02 ± 283.34 | 275 ± 804.76 | 1.000 |
| Total bilirubin (mg/dL) | 0.5 ± 0.33 | 0.43 ± 0.22 | 0.64 ± 0.46 | 0.290 |
| AST (GOT) (U/L) | 67.78 ± 87.04 | 65.26 ± 69.38 | 73 ± 116.64 | 1.000 |
| ALT (GPT) (U/L) | 72.49 ± 102.95 | 72.23 ± 85.4 | 73.03 ± 133.77 | 0.790 |
| Alkaline phosphatase (U/L) | 89.24 ± 68.67 | 82.4 ± 46.22 | 103.16 ± 99.52 | 0.840 |
| Proteins totals (g/dL) | 6.36 ± 0.67 | 6.47 ± 0.69 | 6.08 ± 0.52 | 0.320 |
| Albumin (g/dL) | 3.5 ± 0.47 | 3.54 ± 0.5 | 3.4 ± 0.38 | 0.620 |
| Procalcitonin (ng/ml) | 3.75 ± 15.45 | 4 ± 17.79 | 3.28 ± 9.72 | 1.000 |
| C-reactive protein (mg/dl) | 10.63 ± 8.97 | 9.69 ± 9.31 | 12.59 ± 8 | 0.350 |
| Troponin I (ng/l) | 21.9 ± 33.27 | 17.06 ± 11.97 | 30.84 ± 53.25 | 0.780 |
| Ferritin (ng/ml) | 2160.21 ± 2633.15 | 1969.03 ± 2584.58 | 2552.63 ± 2758.97 | 0.620 |
| Interleukin 6 (pg/ml) | 147.65 ± 402.73 | 90.94 ± 127.76 | 273.39 ± 690.72 | 0.140 |
| **Hemogram** |  |  |  |  |
| Hemoglobin (g/dL) | 12.98 ± 1.83 | 12.98 ± 1.75 | 12.98 ± 2.04 | 1.000 |
| Hematocrits (%) | 39.74 ± 5.38 | 39.87 ± 4.84 | 39.47 ± 6.43 | 0.930 |
| Leukocytes (10³u/μl) | 9.44 ± 4.56 | 9.12 ± 4.06 | 10.11 ± 5.48 | 0.770 |
| Neutrophils T (10³u/μl) | 8.09 ± 4.34 | 7.74 ± 3.93 | 8.83 ± 5.1 | 0.740 |
| Lymphocytes T (10³u/μl) | 0.86 ± 0.44 | 0.91 ± 0.47 | 0.77 ± 0.36 | 0.490 |
| Monocytes T (10³u/μl) | 0.45 ± 0.31 | 0.43 ± 0.27 | 0.48 ± 0.39 | 0.990 |
| Eosinophils T (10³u/μl) | 0.03 ± 0.06 | 0.03 ± 0.06 | 0.02 ± 0.05 | 0.890 |
| Basophilos T (10³u/μl) | 0.01 ± 0.01 | 0.01 ± 0.01 | 0.02 ± 0.01 | 0.550 |
| Plateles (10³u/μl) | 266.81 ± 112.85 | 266.65 ± 105.81 | 267.16 ± 128.23 | 0.970 |
| **Coagulation** |  |  |  |  |
| T. prothrombin (activity) (%) | 101.27 ± 19.65 | 103.03 ± 19.57 | 97.7 ± 19.65 | 0.740 |
| T. prothrombin (seconds) | 10.92 ± 0.86 | 10.85 ± 0.73 | 11.05 ± 1.07 | 0.950 |
| T. prothrombin (ratio) | 1.03 ± 0.08 | 1.02 ± 0.06 | 1.04 ± 0.1 | 0.740 |
| INR Plasma | 1.04 ± 0.09 | 1.03 ± 0.07 | 1.06 ± 0.12 | 0.740 |
| APTT (seconds) | 28.8 ± 4.88 | 29.03 ± 5.39 | 28.33 ± 3.64 | 0.970 |
| APTT (ratio) | 1.08 ± 0.18 | 1.09 ± 0.2 | 1.05 ± 0.13 | 0.740 |
| D-Dimer (μg/l) | 2918.4 ± 6946.69 | 2048.75 ± 6281.86 | 4773.67 ± 7987.27 | 0.170 |

*Values reflect p-values adjusted using the Benjamini & Hochberg method.

GFT: Glomerular Filtration Rate, LDH: Lactate dehydrogenase, AST: Aspartate aminotransferase, ALT: Alanine aminotransferase, INR: International normalized ratio, APTT: activated partial thromboplastin time.

**Table E4**: Clinical Outcomes

A. Training vs. Validation Cohorts

|  | **ALL**  (n = 111) | **TRAINING**  (n = 89) | **VALIDATION**  (n = 22) | **p-value*** |
| --- | --- | --- | --- | --- |
| Total hospital (days) | 9 (7 - 12) | 10 (7 - 11) | 9 (7 - 13) | 1.000 |
| Total ICU (days) | 3 (1 - 5) | 3 (1 - 6) | 2 (1 - 4) | 0.890 |
| Total IMV (days) | 3 (1 - 6) | 3 (1 - 6) | 2 (1 - 4) | 0.790 |
| Exitus ICU, n (%) | 1 (4.55) | 0 (0) | 1 (11.11) | 1.000 |
| Exitus hospital, n (%) | 1 (4.55) | 0 (0) | 1 (11.11) | 1.000 |

*Values reflect p-values adjusted using the Benjamini & Hochberg method.

ICU: intensive care unit, IMV: Invasive Mechanical Ventilation.

B. Training Cohort

|  | **ALL**  (n = 89) | **SUCCESS**  (n = 54) | **FAILURE**  (n = 35) | **p-value*** |
| --- | --- | --- | --- | --- |
| Total hospital (days) | 23 (16 - 32) | 17 (13 - 26) | 32 (25 - 39) | **< 0.001** |
| Total ICU (days) | 7 (4 - 17) | 4 (3 - 6) | 18 (12 - 28) | **< 0.001** |
| Total IMV (days) | 0 (0 - 10) | 0 (0 - 0) | 13 (8 - 26) | **< 0.001** |
| Exitus ICU, n (%) | 9 (10.11) | 0 (0) | 9 (25.71) | **< 0.001** |
| Exitus hospital, n (%) | 10 (11.24) | 0 (0) | 10 (28.57) | **< 0.001** |

*Values reflect p-values adjusted using the Benjamini & Hochberg method.

ICU: intensive care unit, IMV: Invasive Mechanical Ventilation.

# C. Validation Cohort

|  | **ALL**  (n = 22) | **SUCCESS**  (n = 13) | **FAILURE**  (n = 9) | **p-value*** |
| --- | --- | --- | --- | --- |
| Total hospital (days) | 25 (13 - 40) | 14 (10 - 24) | 49 (34 - 55) | 0.072 |
| Total ICU (days) | 7 (4 - 19) | 5 (3 - 7) | 32 (12 - 38) | 0.053 |
| Total IMV (days) | 0 (0 - 11) | 0 (0 - 0) | 25 (8 - 30) | **0.002** |
| Exitus ICU, n (%) | 1 (4.55) | 0 (0) | 1 (11.11) | 1.000 |
| Exitus hospital, n (%) | 1 (4.55) | 0 (0) | 1 (11.11) | 1.000 |

*Values reflect p-values adjusted using the Benjamini & Hochberg method.

ICU: intensive care unit, IMV: Invasive Mechanical Ventilation.

# D. Training and Validation Cohorts

|  | **ALL**  (n = 111) | **SUCCESS**  (n = 67) | **FAILURE**  (n = 44) | **p-value*** |
| --- | --- | --- | --- | --- |
| Total hospital (days) | 25 (13 - 40) | 15 (10 - 25) | 49 (34 - 55) | **< 0.001** |
| Total ICU (days) | 7 (4 - 19) | 5 (3 - 7) | 32 (12 - 39) | **< 0.001** |
| Total IMV (days) | 0 (0 - 11) | 0 (0 – 0) | 13 (8 - 26) | **< 0.001** |
| Exitus ICU, n (%) | 10 (9.01) | 0 (0.00) | 10 (22.73) | **< 0.001** |
| Exitus hospital, n (%) | 11 (9.91) | 0 (0.00) | 11 (25.00) | **< 0.001** |

*Values reflect p-values adjusted using the Benjamini & Hochberg method.

ICU: intensive care unit, IMV: Invasive Mechanical Ventilation.

**Table E5.** Abbreviations and Units of the Analyzed Metabolites

| **Variable** | **Full name** | **Units** |
| --- | --- | --- |
| 20aDHE | 20α-Dihydrocortisone | ng/mL |
| 20aDHF | 20α-Dihydrocortisol | ng/mL |
| 20bDHE | 20β-Dihydrocortisone | ng/mL |
| 20bDHF | 20β-Dihydrocortisol | ng/mL |
| 3OHKynurenine | 3-hydroxykynurenine | ng/mL |
| 5aTHF | 5α-Tetrahydrocortisol | ng/mL |
| 5bTHF | 5β-Tetrahydrocortisol | ng/mL |
| 5HIAA | 5-hydroxyindoleacetic acid | ng/mL |
| 6OHF | 6β-Hydroxycortisol | ng/mL |
| A | 11-dehydrocorticosterone | ng/mL |
| Acetilcarnitine | Acetilcarnitine | ng/mL |
| aCOBut | α-Ketobutyric acid | ng/mL |
| AED | Androstenedione | ng/mL |
| aKetoglutaric | α-Ketoglutaric acid | ng/mL |
| aKIC | α-Ketoisocaproic acid | RESPONSE |
| Anthranilic | Anthranilic acid | RESPONSE |
| aOHBut | α-Hydroxybutyric acid | ng/mL |
| aOHGlut | α-Hydroxyglutaric acid | RESPONSE |
| B | Corticosterone | ng/mL |
| betaine | Betaine | RESPONSE |
| bOHbMetBut | β-Hydroxy β-methylbutyric acid | RESPONSE |
| bOHBut | β-Hydroxybutyric acid | ng/mL |
| Butyric | Butyric acid | RESPONSE |
| Carnitine | Carnitine | ng/mL |
| Cer181140 | Ceramide 14:0 | RESPONSE |
| Cer181160 | Ceramide 16:0 | ng/mL |
| Cer181180 | Ceramide 18:0 | ng/mL |
| Cer181200 | Ceramide 20:0 | ng/mL |
| Cer181220 | Ceramide 22:0 | ng/mL |
| Cer181240 | Ceramide 24:0 | ng/mL |
| Cer181241 | Ceramide 24:1 | ng/mL |
| Choline | Choline | ng/mL |
| Citric | Citric acid | ng/mL |
| Cortisol | Cortisol | ng/mL |
| Cortisone | Cortisone | ng/mL |
| Creatine | Creatine | ng/mL |
| Creatinine | Creatinine | ng/mL |
| DAG160 | Diacylglycerol 16:0,16:0 | ng/mL |
| DAG160180 | Diacylglycerol 16:0,18:0 | ng/mL |
| DAG160182 | Diacylglycerol 16:0,18:2 | RESPONSE |
| DAG161 | Diacylglycerol 16:1,16:1 | RESPONSE |
| DAG180 | Diacylglycerol 18:0,18:0 | ng/mL |
| DAG180181 | Diacylglycerol 18:0,18:1 | RESPONSE |
| DAG180182 | Diacylglycerol 18:0,18:2 | ng/mL |
| DAG180204 | Diacylglycerol 18:0,20:4 | ng/mL |
| DAG181 | Diacylglycerol 18:1,18:1 | ng/mL |
| DAG181160 | Diacylglycerol 18:1,16:0 | RESPONSE |
| DAG182 | Diacylglycerol 18:2,18:2 | RESPONSE |
| Decanoic | Decanoic acid | ng/mL |
| Dodecanoic | Dodecanoic acid | ng/mL |
| Gluconic | Gluconic acid | ng/mL |
| Glutamic | Glutamic acid | ng/mL |
| Glutamine | Glutamine | ng/mL |
| HexCer140 | Hexosylceramide 14:0 | RESPONSE |
| HexCer160 | Hexosylceramide 16:0 | ng/mL |
| HexCer161 | Hexosylceramide 16:1 | RESPONSE |
| HexCer180 | Hexosylceramide 18:0 | ng/mL |
| HexCer181 | Hexosylceramide 18:1 | ng/mL |
| HexCer200 | Hexosylceramide 20:0 | RESPONSE |
| HexCer220 | Hexosylceramide 22:0 | RESPONSE |
| HexCer240 | Hexosylceramide 24:0 | RESPONSE |
| HexCer241 | Hexosylceramide 24:1 | ng/mL |
| Hippuric | Hippuric acid | ng/mL |
| Indole3Acetic | Indole-3-acetic acid | RESPONSE |
| Indole3Lactic | Indole-3-lactic acid | RESPONSE |
| Isoleucine | Isoleucine | ng/mL |
| Isovaleric | Isovaleric acid | RESPONSE |
| Kynurenic | Kynurenic acid | ng/mL |
| Kynurenine | Kynurenine | ng/mL |
| Lactic | Lactic acid | ng/mL |
| Leucine | Leucine | ng/mL |
| LPC140 | Lysophosphatidylcholine 14:0 | ng/mL |
| LPC150 | Lysophosphatidylcholine 15:0 | ng/mL |
| LPC160 | Lysophosphatidylcholine 16:0 | ng/mL |
| LPC161 | Lysophosphatidylcholine 16:1 | RESPONSE |
| LPC180 | Lysophosphatidylcholine 18:0 | ng/mL |
| LPC181 | Lysophosphatidylcholine 18:1 | ng/mL |
| LPC182 | Lysophosphatidylcholine 18:2 | RESPONSE |
| LPC183 | Lysophosphatidylcholine 18:3 | RESPONSE |
| LPC200 | Lysophosphatidylcholine 20:0 | RESPONSE |
| LPC201 | Lysophosphatidylcholine 20:1 | RESPONSE |
| LPC202 | Lysophosphatidylcholine 20:2 | RESPONSE |
| LPC203 | Lysophosphatidylcholine 20:3 | RESPONSE |
| LPC204 | Lysophosphatidylcholine 20:4 | RESPONSE |
| LPC205 | Lysophosphatidylcholine 20:5 | RESPONSE |
| LPC220 | Lysophosphatidylcholine 22:0 | ng/mL |
| LPC224 | Lysophosphatidylcholine 22:4 | RESPONSE |
| LPC225 | Lysophosphatidylcholine 22:5 | RESPONSE |
| LPC226 | Lysophosphatidylcholine 22:6 | RESPONSE |
| MAG140 | Monoacylglycerol 14:0 | RESPONSE |
| MAG160 | Monoacylglycerol 16:0 | ng/mL |
| MAG181 | Monoacylglycerol 18:1 | ng/mL |
| MAG182 | Monoacylglycerol 18:2 | ng/mL |
| MAG204 | Monoacylglycerol 20:4 | ng/mL |
| Malic | Malic acid | ng/mL |
| MalonylSemiald | Malonyl semialdehyde | ng/mL |
| Methionine | Methionine | ng/mL |
| Nicotinamide | Nicotinamide | RESPONSE |
| Octanoic | Octanoic acid | ng/mL |
| Phenylalanine | Phenylalanine | ng/mL |
| Prog | Progesterone | ng/mL |
| Propionic | Propionic acid | RESPONSE |
| Pyruvic | Pyruvic acid | ng/mL |
| Serotonin | Serotonin | ng/mL |
| Succinic | Succinic acid | ng/mL |
| T | Testosterone | ng/mL |
| TMAO | Trimethylamine N-oxide | RESPONSE |
| Tryptophan | Tryptophan | ng/mL |
| Tyrosine | Tyrosine | ng/mL |
| Valeric | Valeric acid | RESPONSE |
| Valine | Valine | ng/mL |

Response = instrumental area of the analyte/instrumental area of the internal standard

**Table E6**: Abbreviations and Units of the Analyzed Metabolomic ratios.

| Variable | Full name |
| --- | --- |
| 20bDHF/Cortisol | 20β-Dihydrocortisol/Cortisol |
| Glutamic/aKetoglutaric | Glutamic acid/α-Ketoglutaric acid |
| Glutamine/Glutamic | Glutamine/Glutamic acid |
| 20aDHF/Cortisol | 20α-Dihydrocortisol/Cortisol |
| 20aDHF/Cortisone | 20α-Dihydrocortisone/Cortisone |
| 20bDHF/Cortisone | 20β-Dihydrocortisone/Cortisone |
| 3OHKynurenine/Kynurenine | 3-hydroxykynurenine/Kynurenine |
| 5HIAA/Tryptophan | 5-hydroxyindoleacetic acid/Tryptophan |
| aKetoglutaric/Citric | α-Ketoglutaric acid/Citric acid |
| aKIC/Leucine | α-Ketoisocaproic acid/Leucine |
| BCAA/AAA | Σ Branched-chain amino acids/Σ Aromatic amino acids |
| Citric/Lactic | Citric acid/Lactic acid |
| Citric/Malic | Citric acid/Malic acid |
| Citric/Pyruvic | Citric acid/Pyruvic acid |
| Cortisol/Cortisone | Cortisol/Cortisone |
| Creatine/Creatinine | Creatine/Creatinine |
| HexCer160/Cer181160 | Hexosylceramide 16:0/Ceramide 16:0 |
| HexCer180/Cer181180 | Hexosylceramide 18:0/Ceramide 18:0 |
| HexCer200/Cer181200 | Hexosylceramide 20:0/Ceramide 20:0 |
| HexCer220/Cer181220 | Hexosylceramide 22:0/Ceramide 22:0 |
| HexCer24/Cer1811241 | Hexosylceramide 24:1/Ceramide 24:1 |
| HexCer240/Cer181240 | Hexosylceramide 24:0/Ceramide 24:0 |
| Kynurenic/Kynurenine | Kynurenic acid/Kynurenine |
| Kynurenine/Tryptophan | Kynurenine/Tryptophan |
| Lactic/Pyruvic | Lactic acid/Pyruvic acid |
| Malic/Succinic | Malic acid/Succinic acid |
| Succinic/aKetoglutaric | Succinic acid/α-Ketoglutaric acid |
| TMAO/Choline | Trimethylamine N-oxide/Choline |

**Table E7**: Metabolites analyzed.

| **A.** Training vs. Validation Cohorts |  |  |  |  |
| --- | --- | --- | --- | --- |
|  | **ALL** (n = 111) | **TRAINING** (n = 89) | **VALIDATION** (n = 22) | **p-value*** |
| Valine (ng/mL) | 9946.63 ± 3040.96 | 10161.45 ± 3047.33 | 9036.23 ± 2909.24 | 0.480 |
| Leucine (ng/mL) | 24951.34 ± 7675.27 | 24949.91 ± 7809.28 | 24957.44 ± 7261.17 | 1.000 |
| Isoleucine (ng/mL) | 13210.91 ± 4187.01 | 13524.38 ± 4197.24 | 11882.39 ± 3967.32 | 0.290 |
| Methionine (ng/mL) | 3241.52 ± 1432.25 | 3153.46 ± 1358.41 | 3614.76 ± 1697.01 | 0.680 |
| Tryptophan (ng/mL) | 6035.73 ± 1741.52 | 6016.54 ± 1664.2 | 6117.05 ± 2081.95 | 0.960 |
| Serotonin (ng/mL) | 9.2 ± 11 | 9.06 ± 10.29 | 9.79 ± 13.91 | 1.000 |
| 5-hydroxyindoleacetic acid (ng/mL) | 19.54 ± 43.09 | 20.66 ± 47.25 | 14.81 ± 16.35 | 0.400 |
| Kynurenine (ng/mL) | 776.66 ± 489.33 | 785.04 ± 513.23 | 741.18 ± 379.92 | 0.990 |
| 3-hydroxykynurenine (ng/mL) | 24.22 ± 50 | 21.5 ± 48.5 | 35.73 ± 55.69 | **0.021** |
| Kynurenic acid (ng/mL) | 87.25 ± 225.6 | 96.86 ± 249.41 | 46.51 ± 39.11 | 0.880 |
| Anthranilic acid (a.u.) | 13914.81 ± 11480.52 | 14299.19 ± 12135.53 | 12285.78 ± 8178.43 | 0.790 |
| Phenylalanine (ng/mL) | 7489.6 ± 1716.1 | 7536.24 ± 1730.37 | 7291.94 ± 1680.8 | 0.900 |
| Tyrosine (ng/mL) | 6520.62 ± 2128 | 6606.19 ± 2174.83 | 6157.99 ± 1922.88 | 0.840 |
| Nicotinamide (a.u.) | 0.26 ± 0.2 | 0.27 ± 0.2 | 0.21 ± 0.22 | 0.150 |
| Creatinine (ng/mL) | 1667.68 ± 1843.56 | 1712.91 ± 1929.35 | 1484.7 ± 1469.84 | 0.880 |
| Creatine (ng/mL) | 2366.27 ± 2629.71 | 2450.09 ± 2858.89 | 2027.17 ± 1350.35 | 0.890 |
| Choline (ng/mL) | 140.23 ± 89.91 | 143.4 ± 91.85 | 127.4 ± 82.3 | 0.780 |
| Glutamine (ng/mL) | 14860.73 ± 5062.74 | 14137.64 ± 4617.7 | 17785.99 ± 5807.28 | 0.087 |
| Glutamic acid (ng/mL) | 2395.13 ± 1458.6 | 2555.7 ± 1540.29 | 1745.53 ± 806.8 | 0.082 |
| Acetilcarnitine (ng/mL) | 310.33 ± 226.9 | 317.31 ± 242.29 | 282.13 ± 150.45 | 0.960 |
| Carnitine (ng/mL) | 1725.51 ± 760.42 | 1718.98 ± 806.05 | 1751.93 ± 552.54 | 0.820 |
| Trimethylamine N-oxide (a.u.) | 0.43 ± 1.23 | 0.46 ± 1.35 | 0.29 ± 0.52 | 0.880 |
| Betaine (a.u.) | 2.48 ± 1.42 | 2.53 ± 1.47 | 2.3 ± 1.19 | 0.790 |
| Diacylglycerol 16:1,16:1 (a.u.) | 1.47 ± 0.81 | 1.48 ± 0.82 | 1.46 ± 0.79 | 1.000 |
| Diacylglycerol 16:0,16:0 (ng/mL) | 483.56 ± 360.67 | 362.49 ± 255.53 | 973.34 ± 307.11 | **< 0.001** |
| Diacylglycerol 16:0,18:2 (a.u.) | 18.61 ± 13.65 | 19.38 ± 14.72 | 15.48 ± 7.5 | 0.870 |
| Diacylglycerol 18:1,16:0 (a.u.) | 84.66 ± 56.9 | 89.5 ± 59.86 | 65.12 ± 38.04 | 0.290 |
| Diacylglycerol 16:0,18:0 (ng/mL) | 1440.93 ± 2030.06 | 639.54 ± 412.18 | 4682.93 ± 2672.47 | **< 0.001** |
| Diacylglycerol 18:2,18:2 (a.u.) | 5.89 ± 5.74 | 4.7 ± 4.86 | 10.73 ± 6.54 | **< 0.001** |
| Diacylglycerol 18:0,18:2 (ng/mL) | 1545.95 ± 1232.4 | 1600.04 ± 1293.68 | 1327.11 ± 938.03 | 0.790 |
| Diacylglycerol 18:1,18:1 (ng/mL) | 2389.73 ± 1243.67 | 2394.95 ± 1334.43 | 2368.59 ± 799.49 | 0.900 |
| Diacylglycerol 18:0,18:1 (a.u.) | 3.37 ± 3.91 | 3.17 ± 3.24 | 4.17 ± 5.95 | 0.820 |
| Diacylglycerol 18:0,18:0 (ng/mL) | 28.34 ± 42.26 | 9.37 ± 6.35 | 105.1 ± 38.56 | **< 0.001** |
| Diacylglycerol 18:0,20:4 (ng/mL) | 334.76 ± 319.91 | 285.56 ± 264.3 | 533.79 ± 437.72 | **0.014** |
| Monoacylglycerol 14:0 (a.u.) | 18.17 ± 20.15 | 16.55 ± 20.7 | 24.73 ± 16.61 | **0.018** |
| Monoacylglycerol 16:0 (ng/mL) | 20862.27 ± 32038.92 | 8189.86 ± 7683.26 | 72127.92 ± 41177.37 | < 0.001 |
| Monoacylglycerol 18:2 (ng/mL) | 2472.95 ± 1997.54 | 2395.35 ± 2120.87 | 2786.87 ± 1386.72 | 0.240 |
| Monoacylglycerol 18:1 (ng/mL) | 3424.99 ± 2469.71 | 3473.89 ± 2677.77 | 3227.14 ± 1360.48 | 0.750 |
| Monoacylglycerol 20:4 (ng/mL) | 221.91 ± 185.93 | 201.89 ± 183.7 | 302.9 ± 176.38 | **0.021** |
| Ceramide 14:0 (a.u.) | 7.27 ± 7.66 | 6.63 ± 8.06 | 9.89 ± 5.11 | **0.017** |
| Ceramide 16:0 (ng/mL) | 1094.19 ± 645.27 | 1088.97 ± 699.75 | 1115.31 ± 358.49 | 0.660 |
| Ceramide 18:0 (ng/mL) | 558.68 ± 352.16 | 532.07 ± 357.23 | 666.33 ± 315.79 | 0.230 |
| Ceramide 20:0 (ng/mL) | 946.69 ± 1013.38 | 760.14 ± 583.07 | 1701.35 ± 1791.77 | 0.100 |
| Ceramide 22:0 (ng/mL) | 582.82 ± 493.1 | 526.21 ± 487.7 | 811.82 ± 456.52 | **0.024** |
| Ceramide 24:0 (ng/mL) | 1210.74 ± 650.74 | 1197.75 ± 699.94 | 1263.29 ± 401.9 | 0.630 |
| Ceramide 24:1 (ng/mL) | 2439.03 ± 1139.01 | 2391.53 ± 1221.68 | 2631.19 ± 702.19 | 0.410 |
| Hexosylceramide 14:0 (a.u.) | 0.81 ± 0.89 | 0.74 ± 0.92 | 1.08 ± 0.71 | 0.055 |
| Hexosylceramide 16:0 (ng/mL) | 20649.71 ± 15643.63 | 18345.97 ± 14847.37 | 29969.38 ± 15640.68 | **0.006** |
| Hexosylceramide 18:0 (ng/mL) | 167.27 ± 88.45 | 167.56 ± 94.72 | 166.1 ± 58.17 | 0.940 |
| Hexosylceramide 20:0 (a.u.) | 0.22 ± 0.14 | 0.22 ± 0.15 | 0.21 ± 0.09 | 1.000 |
| Hexosylceramide 22:0 (a.u.) | 7.46 ± 5.13 | 7.29 ± 5.25 | 8.12 ± 4.66 | 0.720 |
| Hexosylceramide 24:0 (a.u.) | 17.05 ± 12.48 | 17.51 ± 12.76 | 15.2 ± 11.36 | 0.790 |
| Hexosylceramide 16:1 (a.u.) | 3.71 ± 3.3 | 3.76 ± 3.59 | 3.5 ± 1.66 | 0.760 |
| Hexosylceramide 18:1 (ng/mL) | 304.66 ± 258.19 | 277.04 ± 247.98 | 416.4 ± 274.17 | **0.022** |
| Hexosylceramide 24:1 (ng/mL) | 4946.83 ± 2995.55 | 4755.09 ± 3129.66 | 5722.49 ± 2274.25 | 0.160 |
| Lysophosphatidylcholine 14:0 (ng/mL) | 1146.25 ± 888.43 | 1110.67 ± 894.74 | 1290.18 ± 867.57 | 0.430 |
| Lysophosphatidylcholine 15:0 (ng/mL) | 1418.32 ± 1172.35 | 1444.29 ± 1260.92 | 1313.24 ± 722.71 | 0.880 |
| Lysophosphatidylcholine 16:0 (ng/mL) | 126584.08 ± 42350.09 | 125981.45 ± 44083.56 | 129021.97 ± 35260.57 | 0.790 |
| Lysophosphatidylcholine 18:0 (ng/mL) | 68960.38 ± 30396.37 | 67611.29 ± 31839.21 | 74418.03 ± 23510.19 | 0.380 |
| Lysophosphatidylcholine 20:0 (a.u.) | 0.68 ± 0.77 | 0.69 ± 0.84 | 0.65 ± 0.39 | 0.780 |
| Lysophosphatidylcholine 22:0 (ng/mL) | 97.92 ± 136.05 | 87.34 ± 90.66 | 140.7 ± 245.2 | 0.630 |
| Lysophosphatidylcholine 22:6 (a.u.) | 5.01 ± 4.67 | 4.82 ± 3.43 | 5.77 ± 7.99 | 1.000 |
| Lysophosphatidylcholine 16:1 (a.u.) | 1.31 ± 0.75 | 1.25 ± 0.74 | 1.51 ± 0.78 | 0.420 |
| Lysophosphatidylcholine 18:1 (ng/mL) | 78278.79 ± 29940.57 | 74384.62 ± 28072.69 | 94032.47 ± 32694.78 | **0.046** |
| Lysophosphatidylcholine 18:3 (a.u.) | 0.23 ± 0.21 | 0.21 ± 0.19 | 0.35 ± 0.23 | **0.005** |
| Lysophosphatidylcholine 18:2 (a.u.) | 22.26 ± 8.36 | 20.95 ± 7.84 | 27.53 ± 8.46 | **0.006** |
| Lysophosphatidylcholine 20:1 (a.u.) | 0.28 ± 0.2 | 0.27 ± 0.21 | 0.29 ± 0.13 | 0.630 |
| Lysophosphatidylcholine 20:2 (a.u.) | 0.39 ± 0.24 | 0.33 ± 0.19 | 0.6 ± 0.28 | **< 0.001** |
| Lysophosphatidylcholine 20:3 (a.u.) | 1.95 ± 1.18 | 1.63 ± 0.81 | 3.24 ± 1.56 | **< 0.001** |
| Lysophosphatidylcholine 20:4 (a.u.) | 6.41 ± 3.07 | 5.95 ± 2.9 | 8.31 ± 3.11 | **0.021** |
| Lysophosphatidylcholine 20:5 (a.u.) | 0.47 ± 0.62 | 0.39 ± 0.28 | 0.79 ± 1.24 | 0.480 |
| Lysophosphatidylcholine 22:4 (a.u.) | 0.6 ± 0.54 | 0.53 ± 0.4 | 0.86 ± 0.88 | 0.400 |
| Lysophosphatidylcholine 22:5 (a.u.) | 1.04 ± 1.39 | 0.88 ± 0.64 | 1.68 ± 2.81 | 0.540 |
| Lactic acid (ng/mL) | 127293.63 ± 70223.36 | 130810.91 ± 68471.19 | 113064.62 ± 76941.28 | 0.410 |
| Pyruvic acid (ng/mL) | 18186.96 ± 8580.17 | 17176.3 ± 8215.99 | 22275.54 ± 8995.32 | 0.150 |
| Citric acid (ng/mL) | 2109.25 ± 902.81 | 2213.02 ± 969 | 1689.45 ± 323.22 | **0.034** |
| α-Ketoglutaric acid (ng/mL) | 682.17 ± 347.78 | 670.66 ± 359.75 | 728.75 ± 297.32 | 0.450 |
| Succinic acid (ng/mL) | 165.11 ± 76.75 | 174.69 ± 75.32 | 126.34 ± 71.53 | **0.005** |
| Malic acid (ng/mL) | 420.48 ± 286.65 | 431.89 ± 308.17 | 374.34 ± 172.23 | 0.840 |
| α-Ketobutyric acid (ng/mL) | 610112.88 ± 436997.03 | 585804.73 ± 443092.44 | 708450.36 ± 406134.49 | 0.400 |
| α-Hydroxybutyric acid (ng/mL) | 35201819.97 ± 16615856.41 | 35350670.73 ± 16713449.52 | 34599650.96 ± 16587027.69 | 1.000 |
| β-Hydroxybutyric acid (ng/mL) | 14048266.99 ± 18450914.2 | 15284424.31 ± 19837319.25 | 9047448.74 ± 10076170.76 | 0.570 |
| α-Hydroxyglutaric acid (a.u.) | 0.03 ± 0.02 | 0.03 ± 0.02 | 0.03 ± 0.02 | 0.640 |
| Hippuric acid (ng/mL) | 36.66 ± 87.78 | 42.94 ± 96.47 | 11.25 ± 22.81 | 0.150 |
| α-Ketoisocaproic acid (a.u.) | 1.85 ± 1.18 | 1.92 ± 1.24 | 1.55 ± 0.81 | 0.640 |
| β-Hydroxy β-methylbutyric acid (a.u.) | 0.09 ± 0.07 | 0.1 ± 0.07 | 0.05 ± 0.03 | **0.006** |
| Propionic acid (a.u.) | 4.52 ± 4.33 | 4.94 ± 4.7 | 2.83 ± 1.29 | 0.170 |
| Butyric acid (a.u.) | 0.35 ± 0.18 | 0.34 ± 0.18 | 0.4 ± 0.14 | 0.360 |
| Valeric acid (a.u.) | 0.37 ± 0.52 | 0.43 ± 0.56 | 0.16 ± 0.09 | **0.025** |
| Isovaleric acid (a.u.) | 0.84 ± 1.15 | 0.96 ± 1.25 | 0.34 ± 0.24 | **0.016** |
| Octanoic acid (ng/mL) | 516943.85 ± 1323373.35 | 418001.41 ± 963379.85 | 917210.98 ± 2252786.96 | **0.024** |
| Decanoic acid (ng/mL) | 1444205.72 ± 3634559.48 | 1219143.01 ± 2727207.25 | 2354686.65 ± 6078231.5 | 0.600 |
| Dodecanoic acid (ng/mL) | 4562445.14 ± 4094657.4 | 4861093.71 ± 4097257.72 | 3354275.93 ± 3945651.52 | 0.240 |
| Indole-3-acetic acid (a.u.) | 33370.82 ± 38036.48 | 35557.1 ± 41310.95 | 24526.29 ± 18019.14 | 0.880 |
| Indole-3-lactic acid (a.u.) | 6786.22 ± 6567.99 | 6997.55 ± 7175.54 | 5931.29 ± 3040.97 | 1.000 |
| Malonyl semialdehyde (ng/mL) | 39.34 ± 59.91 | 41.78 ± 64.89 | 29.48 ± 32.07 | 0.930 |
| Gluconic acid (ng/mL) | 7595.55 ± 36982.96 | 4397.6 ± 12771.35 | 20532.7 ± 79133.87 | 0.170 |
| Cortisol (ng/mL) | 9.34 ± 10.54 | 9.71 ± 10.61 | 7.91 ± 10.36 | 0.610 |
| 20α-Dihydrocortisol (ng/mL) | 1.13 ± 2.64 | 1.16 ± 2.7 | 1.02 ± 2.43 | 0.560 |
| 20β-Dihydrocortisol (ng/mL) | 1.31 ± 2.59 | 1.3 ± 2.41 | 1.34 ± 3.28 | 0.640 |
| 5α-Tetrahydrocortisol (ng/mL) | 3.64 ± 9.4 | 3.51 ± 8.57 | 4.12 ± 12.37 | 0.760 |
| 5β-Tetrahydrocortisol (ng/mL) | 1.06 ± 2.72 | 1.15 ± 2.97 | 0.71 ± 1.36 | 0.750 |
| 6β-Hydroxycortisol (ng/mL) | 0.22 ± 0.5 | 0.25 ± 0.55 | 0.1 ± 0.18 | 0.790 |
| Cortisone (ng/mL) | 1.15 ± 1.41 | 1.13 ± 1.34 | 1.24 ± 1.7 | 0.950 |
| 20α-Dihydrocortisone (ng/mL) | 0.24 ± 0.32 | 0.24 ± 0.33 | 0.23 ± 0.29 | 0.950 |
| 20β-Dihydrocortisone (ng/mL) | 0.11 ± 0.21 | 0.11 ± 0.22 | 0.11 ± 0.16 | 0.950 |
| Corticosterone (ng/mL) | 0.16 ± 0.37 | 0.15 ± 0.35 | 0.19 ± 0.45 | 1.000 |
| 11-dehydrocorticosterone (ng/mL) | 0.02 ± 0.03 | 0.01 ± 0.03 | 0.02 ± 0.04 | 0.630 |
| Testosterone (ng/mL) | 0.2 ± 0.17 | 0.2 ± 0.16 | 0.19 ± 0.22 | 0.830 |
| Androstenedione (ng/mL) | 0.08 ± 0.16 | 0.08 ± 0.17 | 0.09 ± 0.14 | 1.000 |
| Progesterone (ng/mL) | 0.03 ± 0.08 | 0.03 ± 0.08 | 0.03 ± 0.03 | 0.820 |
| Kynurenine/Tryptophan | 0.16 ± 0.18 | 0.16 ± 0.19 | 0.15 ± 0.14 | 1.000 |
| 5-hydroxyindoleacetic acid/Tryptophan | 0.01 ± 0.02 | 0.01 ± 0.02 | 0.0055 ± 0.01 | 0.430 |
| 3-hydroxykynurenine/Kynurenine | 0.03 ± 0.04 | 0.02 ± 0.04 | 0.04 ± 0.04 | **0.017** |
| Kynurenic acid/Kynurenine | 0.08 ± 0.08 | 0.08 ± 0.09 | 0.06 ± 0.03 | 0.580 |
| Σ Branched-chain amino acids/Σ Aromatic amin | 2.42 ± 0.55 | 2.43 ± 0.57 | 2.37 ± 0.45 | 0.950 |
| Creatine/Creatinine | 1.8 ± 1.33 | 1.8 ± 1.35 | 1.79 ± 1.27 | 1.000 |
| Glutamine/Glutamic acid | 8.25 ± 5.66 | 7.2 ± 4.02 | 12.51 ± 8.76 | **0.007** |
| Trimethylamine N-oxide/Choline | 0.0043 ± 0.02 | 0.0048 ± 0.02 | 0.0021 ± 0 | 0.680 |
| Hexosylceramide 16:0/Ceramide 16:0 | 21.33 ± 16.45 | 19.4 ± 16 | 29.11 ± 16.31 | **0.035** |
| Hexosylceramide 18:0/Ceramide 18:0 | 0.38 ± 0.22 | 0.39 ± 0.23 | 0.31 ± 0.17 | 0.570 |
| Hexosylceramide 20:0/Ceramide 20:0 | 0.00038 ± 0.00031 | 0.0004 ± 0.00031 | 0.00028 ± 0.0003 | 0.150 |
| Hexosylceramide 22:0/Ceramide 22:0 | 0.02 ± 0.02 | 0.02 ± 0.02 | 0.01 ± 0.01 | 0.200 |
| Hexosylceramide 24:0/Ceramide 24:0 | 0.02 ± 0.01 | 0.02 ± 0.01 | 0.01 ± 0.01 | 0.520 |
| Hexosylceramide 24:1/Ceramide 24:1 | 2.14 ± 1.1 | 2.1 ± 1.13 | 2.29 ± 0.98 | 0.720 |
| Lactic acid/Pyruvic acid | 7.71 ± 4.05 | 8.4 ± 4.19 | 4.91 ± 1.42 | **< 0.001** |
| α-Ketoglutaric acid/Citric acid | 0.35 ± 0.16 | 0.32 ± 0.16 | 0.43 ± 0.15 | **0.015** |
| Succinic acid/α-Ketoglutaric acid | 0.29 ± 0.17 | 0.31 ± 0.17 | 0.19 ± 0.1 | **0.004** |
| Malic acid/Succinic acid | 2.64 ± 1.1 | 2.49 ± 1 | 3.24 ± 1.31 | 0.120 |
| Citric acid/Malic acid | 5.74 ± 2.07 | 5.85 ± 2.03 | 5.31 ± 2.2 | 0.630 |
| Citric acid/Pyruvic acid | 0.15 ± 0.12 | 0.16 ± 0.13 | 0.08 ± 0.03 | **0.003** |
| Citric acid/Lactic acid | 0.02 ± 0.01 | 0.02 ± 0.01 | 0.02 ± 0.01 | 0.990 |
| Glutamic acid/α-Ketoglutaric acid | 4.27 ± 4.14 | 4.69 ± 4.49 | 2.55 ± 1.12 | **0.018** |
| α-Ketoisocaproic acid/Leucine | 7.5e-05 ± 4.4e-05 | 7.8e-05 ± 4.6e-05 | 6.6e-05 ± 3.6e-05 | 0.640 |
| Cortisol/Cortisone | 9.37 ± 7.1 | 10.1 ± 7.65 | 6.48 ± 2.9 | 0.230 |
| 20α-Dihydrocortisol/Cortisol | 0.09 ± 0.08 | 0.09 ± 0.08 | 0.1 ± 0.09 | 1.000 |
| 20β-Dihydrocortisol/Cortisol | 0.11 ± 0.11 | 0.11 ± 0.07 | 0.14 ± 0.19 | 1.000 |
| 20α-Dihydrocortisone/Cortisone | 0.21 ± 0.12 | 0.21 ± 0.12 | 0.2 ± 0.1 | 0.980 |
| 20β-Dihydrocortisone/Cortisone | 0.09 ± 0.07 | 0.09 ± 0.07 | 0.09 ± 0.06 | 0.890 |
| ***** Values reflect p-values adjusted using the Benjamini & Hochberg method. | | | | |

| **A.** Training Cohort |  |  |  |  |
| --- | --- | --- | --- | --- |
|  | **ALL** (n = 89) | **SUCCESS** (n = 54) | **FAILURE** (n = 35) | **p-value*** |
| Valine (ng/mL) | 10161.45 ± 3047.33 | 10473.69 ± 2761.87 | 9679.7 ± 3427.9 | 0.590 |
| Leucine (ng/mL) | 24949.91 ± 7809.28 | 25392.59 ± 7166.05 | 24266.9 ± 8775.1 | 0.700 |
| Isoleucine (ng/mL) | 13524.38 ± 4197.24 | 13783.65 ± 3985.99 | 13124.37 ± 4534.12 | 0.700 |
| Methionine (ng/mL) | 3153.46 ± 1358.41 | 3352.31 ± 1522.35 | 2846.66 ± 1001.83 | 0.500 |
| Tryptophan (ng/mL) | 6016.54 ± 1664.2 | 6265.83 ± 1705.36 | 5631.92 ± 1543.99 | 0.570 |
| Serotonin (ng/mL) | 9.06 ± 10.29 | 8.14 ± 9.59 | 10.48 ± 11.27 | 0.700 |
| 5-hydroxyindoleacetic acid (ng/mL) | 20.66 ± 47.25 | 13 ± 30.04 | 32.48 ± 64.31 | 0.420 |
| Kynurenine (ng/mL) | 785.04 ± 513.23 | 697.58 ± 411.16 | 919.98 ± 622.36 | 0.390 |
| 3-hydroxykynurenine (ng/mL) | 21.5 ± 48.5 | 12.31 ± 25.56 | 35.67 ± 68.78 | 0.490 |
| Kynurenic acid (ng/mL) | 96.86 ± 249.41 | 61.34 ± 144.39 | 151.65 ± 351.3 | 0.490 |
| Anthranilic acid (a.u.) | 14299.19 ± 12135.53 | 11731.05 ± 7983.2 | 18261.45 ± 15974.66 | 0.190 |
| Phenylalanine (ng/mL) | 7536.24 ± 1730.37 | 7360.4 ± 1583.56 | 7807.53 ± 1927.62 | 0.850 |
| Tyrosine (ng/mL) | 6606.19 ± 2174.83 | 6851.62 ± 2202.85 | 6227.52 ± 2105.83 | 0.590 |
| Nicotinamide (a.u.) | 0.27 ± 0.2 | 0.27 ± 0.21 | 0.26 ± 0.18 | 1.000 |
| Creatinine (ng/mL) | 1712.91 ± 1929.35 | 1371.33 ± 1338.65 | 2239.91 ± 2523.86 | 0.450 |
| Creatine (ng/mL) | 2450.09 ± 2858.89 | 2096.78 ± 1352.95 | 2995.2 ± 4218.62 | 1.000 |
| Choline (ng/mL) | 143.4 ± 91.85 | 128.74 ± 57.59 | 166.01 ± 125.68 | 0.720 |
| Glutamine (ng/mL) | 14137.64 ± 4617.7 | 14803.94 ± 5090.46 | 13109.62 ± 3605.93 | 0.490 |
| Glutamic acid (ng/mL) | 2555.7 ± 1540.29 | 2510.89 ± 1677.12 | 2624.85 ± 1322.08 | 0.700 |
| Acetilcarnitine (ng/mL) | 317.31 ± 242.29 | 299.32 ± 209.91 | 345.06 ± 286.27 | 0.890 |
| Carnitine (ng/mL) | 1718.98 ± 806.05 | 1742.65 ± 646.55 | 1682.46 ± 1013.77 | 0.620 |
| Trimethylamine N-oxide (a.u.) | 0.46 ± 1.35 | 0.31 ± 0.73 | 0.7 ± 1.95 | 0.580 |
| Betaine (a.u.) | 2.53 ± 1.47 | 2.31 ± 0.76 | 2.87 ± 2.13 | 0.710 |
| Diacylglycerol 16:1,16:1 (a.u.) | 1.48 ± 0.82 | 1.5 ± 0.69 | 1.44 ± 1 | 0.700 |
| Diacylglycerol 16:0,16:0 (ng/mL) | 362.49 ± 255.53 | 386.82 ± 278.22 | 324.96 ± 214.35 | 0.700 |
| Diacylglycerol 16:0,18:2 (a.u.) | 19.38 ± 14.72 | 19.02 ± 12.89 | 19.93 ± 17.35 | 0.960 |
| Diacylglycerol 18:1,16:0 (a.u.) | 89.5 ± 59.86 | 91.01 ± 59.72 | 87.16 ± 60.87 | 0.890 |
| Diacylglycerol 16:0,18:0 (ng/mL) | 639.54 ± 412.18 | 655.89 ± 395.06 | 614.32 ± 441.99 | 0.850 |
| Diacylglycerol 18:2,18:2 (a.u.) | 4.7 ± 4.86 | 4.06 ± 3.45 | 5.67 ± 6.4 | 0.700 |
| Diacylglycerol 18:0,18:2 (ng/mL) | 1600.04 ± 1293.68 | 1584.34 ± 1320.79 | 1624.28 ± 1269.4 | 0.940 |
| Diacylglycerol 18:1,18:1 (ng/mL) | 2394.95 ± 1334.43 | 2407.05 ± 1354.91 | 2376.29 ± 1321.61 | 0.940 |
| Diacylglycerol 18:0,18:1 (a.u.) | 3.17 ± 3.24 | 3.02 ± 3.44 | 3.4 ± 2.95 | 0.700 |
| Diacylglycerol 18:0,18:0 (ng/mL) | 9.37 ± 6.35 | 8.78 ± 5.61 | 10.28 ± 7.33 | 0.870 |
| Diacylglycerol 18:0,20:4 (ng/mL) | 285.56 ± 264.3 | 222.52 ± 178.99 | 382.82 ± 338.84 | 0.450 |
| Monoacylglycerol 14:0 (a.u.) | 16.55 ± 20.7 | 17.67 ± 20.32 | 14.83 ± 21.45 | 0.700 |
| Monoacylglycerol 16:0 (ng/mL) | 8189.86 ± 7683.26 | 8863.14 ± 8056.67 | 7151.08 ± 7055.22 | 0.700 |
| Monoacylglycerol 18:2 (ng/mL) | 2395.35 ± 2120.87 | 2430.79 ± 2346.86 | 2340.69 ± 1746.85 | 0.960 |
| Monoacylglycerol 18:1 (ng/mL) | 3473.89 ± 2677.77 | 3578.4 ± 2858.03 | 3312.65 ± 2404.52 | 0.960 |
| Monoacylglycerol 20:4 (ng/mL) | 201.89 ± 183.7 | 190.38 ± 158.81 | 219.64 ± 217.91 | 0.940 |
| Ceramide 14:0 (a.u.) | 6.63 ± 8.06 | 6 ± 6.13 | 7.59 ± 10.39 | 0.810 |
| Ceramide 16:0 (ng/mL) | 1088.97 ± 699.75 | 948.49 ± 460.89 | 1305.71 ± 925.47 | 0.400 |
| Ceramide 18:0 (ng/mL) | 532.07 ± 357.23 | 483.17 ± 365.48 | 607.52 ± 335.29 | 0.320 |
| Ceramide 20:0 (ng/mL) | 760.14 ± 583.07 | 662.69 ± 521.72 | 910.5 ± 645.96 | 0.280 |
| Ceramide 22:0 (ng/mL) | 526.21 ± 487.7 | 504.51 ± 534.95 | 559.69 ± 409.4 | 0.600 |
| Ceramide 24:0 (ng/mL) | 1197.75 ± 699.94 | 1205.04 ± 728.49 | 1186.51 ± 663.74 | 1.000 |
| Ceramide 24:1 (ng/mL) | 2391.53 ± 1221.68 | 2191.18 ± 973.01 | 2700.63 ± 1491.65 | 0.580 |
| Hexosylceramide 14:0 (a.u.) | 0.74 ± 0.92 | 0.73 ± 1.06 | 0.76 ± 0.67 | 0.580 |
| Hexosylceramide 16:0 (ng/mL) | 18345.97 ± 14847.37 | 17430.25 ± 15937.06 | 19758.78 ± 13086.27 | 0.570 |
| Hexosylceramide 18:0 (ng/mL) | 167.56 ± 94.72 | 161.29 ± 83.47 | 177.25 ± 110.45 | 0.940 |
| Hexosylceramide 20:0 (a.u.) | 0.22 ± 0.15 | 0.22 ± 0.14 | 0.24 ± 0.17 | 0.960 |
| Hexosylceramide 22:0 (a.u.) | 7.29 ± 5.25 | 7.24 ± 5.55 | 7.38 ± 4.83 | 0.940 |
| Hexosylceramide 24:0 (a.u.) | 17.51 ± 12.76 | 16.76 ± 11.79 | 18.65 ± 14.23 | 0.940 |
| Hexosylceramide 16:1 (a.u.) | 3.76 ± 3.59 | 3.77 ± 4.07 | 3.75 ± 2.75 | 0.740 |
| Hexosylceramide 18:1 (ng/mL) | 277.04 ± 247.98 | 254.65 ± 264.88 | 311.57 ± 218.57 | 0.430 |
| Hexosylceramide 24:1 (ng/mL) | 4755.09 ± 3129.66 | 4486.93 ± 2856.62 | 5168.83 ± 3512.88 | 0.850 |
| Lysophosphatidylcholine 14:0 (ng/mL) | 1110.67 ± 894.74 | 1078.74 ± 849.17 | 1159.94 ± 971.54 | 0.940 |
| Lysophosphatidylcholine 15:0 (ng/mL) | 1444.29 ± 1260.92 | 1339.85 ± 928.98 | 1605.43 ± 1651 | 1.000 |
| Lysophosphatidylcholine 16:0 (ng/mL) | 125981.45 ± 44083.56 | 124034.64 ± 35741.6 | 128985.11 ± 54983.9 | 1.000 |
| Lysophosphatidylcholine 18:0 (ng/mL) | 67611.29 ± 31839.21 | 63023.93 ± 22668.23 | 74688.94 ± 41686.9 | 0.750 |
| Lysophosphatidylcholine 20:0 (a.u.) | 0.69 ± 0.84 | 0.56 ± 0.52 | 0.89 ± 1.16 | 0.460 |
| Lysophosphatidylcholine 22:0 (ng/mL) | 87.34 ± 90.66 | 81.54 ± 75.19 | 96.29 ± 111.02 | 0.710 |
| Lysophosphatidylcholine 22:6 (a.u.) | 4.82 ± 3.43 | 4.67 ± 3.29 | 5.04 ± 3.68 | 0.940 |
| Lysophosphatidylcholine 16:1 (a.u.) | 1.25 ± 0.74 | 1.26 ± 0.75 | 1.24 ± 0.73 | 0.960 |
| Lysophosphatidylcholine 18:1 (ng/mL) | 74384.62 ± 28072.69 | 72065.25 ± 26851.41 | 77963.07 ± 29901.36 | 0.730 |
| Lysophosphatidylcholine 18:3 (a.u.) | 0.21 ± 0.19 | 0.2 ± 0.17 | 0.22 ± 0.22 | 0.940 |
| Lysophosphatidylcholine 18:2 (a.u.) | 20.95 ± 7.84 | 21.65 ± 8.42 | 19.88 ± 6.83 | 0.720 |
| Lysophosphatidylcholine 20:1 (a.u.) | 0.27 ± 0.21 | 0.23 ± 0.13 | 0.34 ± 0.29 | 0.330 |
| Lysophosphatidylcholine 20:2 (a.u.) | 0.33 ± 0.19 | 0.3 ± 0.14 | 0.38 ± 0.24 | 0.500 |
| Lysophosphatidylcholine 20:3 (a.u.) | 1.63 ± 0.81 | 1.65 ± 0.86 | 1.6 ± 0.74 | 1.000 |
| Lysophosphatidylcholine 20:4 (a.u.) | 5.95 ± 2.9 | 5.78 ± 2.57 | 6.19 ± 3.36 | 0.940 |
| Lysophosphatidylcholine 20:5 (a.u.) | 0.39 ± 0.28 | 0.35 ± 0.25 | 0.44 ± 0.31 | 0.670 |
| Lysophosphatidylcholine 22:4 (a.u.) | 0.53 ± 0.4 | 0.56 ± 0.44 | 0.49 ± 0.34 | 1.000 |
| Lysophosphatidylcholine 22:5 (a.u.) | 0.88 ± 0.64 | 0.89 ± 0.67 | 0.87 ± 0.6 | 1.000 |
| Lactic acid (ng/mL) | 130810.91 ± 68471.19 | 129142.11 ± 66637.11 | 133385.62 ± 72119.51 | 1.000 |
| Pyruvic acid (ng/mL) | 17176.3 ± 8215.99 | 17119.88 ± 8919.33 | 17263.34 ± 7119.58 | 0.940 |
| Citric acid (ng/mL) | 2213.02 ± 969 | 2083.64 ± 719.55 | 2412.63 ± 1247.22 | 0.770 |
| α-Ketoglutaric acid (ng/mL) | 670.66 ± 359.75 | 692.4 ± 395.05 | 637.12 ± 299.63 | 0.870 |
| Succinic acid (ng/mL) | 174.69 ± 75.32 | 171.89 ± 65.84 | 179.01 ± 88.86 | 1.000 |
| Malic acid (ng/mL) | 431.89 ± 308.17 | 400.61 ± 192.02 | 480.14 ± 429.38 | 0.980 |
| α-Ketobutyric acid (ng/mL) | 585804.73 ± 443092.44 | 545038.64 ± 411874.93 | 648700.98 ± 486824.95 | 0.830 |
| α-Hydroxybutyric acid (ng/mL) | 35350670.73 ± 16713449.52 | 34744922.06 ± 16564615.67 | 36285254.39 ± 17140421.54 | 0.890 |
| β-Hydroxybutyric acid (ng/mL) | 15284424.31 ± 19837319.25 | 11875472.02 ± 11320065.89 | 20543950.71 ± 27781893.79 | 0.720 |
| α-Hydroxyglutaric acid (a.u.) | 0.03 ± 0.02 | 0.02 ± 0.02 | 0.04 ± 0.03 | 0.320 |
| Hippuric acid (ng/mL) | 42.94 ± 96.47 | 24.79 ± 44.92 | 70.94 ± 140.04 | 0.400 |
| α-Ketoisocaproic acid (a.u.) | 1.92 ± 1.24 | 1.85 ± 1.1 | 2.03 ± 1.44 | 0.980 |
| β-Hydroxy β-methylbutyric acid (a.u.) | 0.1 ± 0.07 | 0.08 ± 0.05 | 0.12 ± 0.09 | 0.700 |
| Propionic acid (a.u.) | 4.94 ± 4.7 | 4.69 ± 3.22 | 5.31 ± 6.39 | 0.850 |
| Butyric acid (a.u.) | 0.34 ± 0.18 | 0.34 ± 0.17 | 0.34 ± 0.2 | 0.980 |
| Valeric acid (a.u.) | 0.43 ± 0.56 | 0.41 ± 0.42 | 0.45 ± 0.74 | 0.620 |
| Isovaleric acid (a.u.) | 0.96 ± 1.25 | 1.02 ± 1.3 | 0.87 ± 1.18 | 0.700 |
| Octanoic acid (ng/mL) | 418001.41 ± 963379.85 | 225652.96 ± 101363.34 | 714767.58 ± 1495561.78 | 0.680 |
| Decanoic acid (ng/mL) | 1219143.01 ± 2727207.25 | 725970.69 ± 585223.62 | 1980037.45 ± 4211206.32 | 0.770 |
| Dodecanoic acid (ng/mL) | 4861093.71 ± 4097257.72 | 4790021.97 ± 4184532.18 | 4970747.25 ± 4016736.41 | 0.930 |
| Indole-3-acetic acid (a.u.) | 35557.1 ± 41310.95 | 32190.43 ± 37790.47 | 40751.4 ± 46315.27 | 0.830 |
| Indole-3-lactic acid (a.u.) | 6997.55 ± 7175.54 | 5569.52 ± 3747.1 | 9200.79 ± 10155.85 | 0.390 |
| Malonyl semialdehyde (ng/mL) | 41.78 ± 64.89 | 47.85 ± 77.56 | 32.41 ± 37.04 | 0.530 |
| Gluconic acid (ng/mL) | 4397.6 ± 12771.35 | 2013.21 ± 3100.23 | 8076.38 ± 19601.44 | 0.580 |
| Cortisol (ng/mL) | 9.71 ± 10.61 | 8.81 ± 10.28 | 11.18 ± 11.13 | 0.450 |
| 20α-Dihydrocortisol (ng/mL) | 1.16 ± 2.7 | 0.88 ± 1.51 | 1.62 ± 3.94 | 0.390 |
| 20β-Dihydrocortisol (ng/mL) | 1.3 ± 2.41 | 0.99 ± 1.72 | 1.81 ± 3.21 | 0.280 |
| 5α-Tetrahydrocortisol (ng/mL) | 3.51 ± 8.57 | 2.62 ± 6.28 | 4.97 ± 11.34 | 0.530 |
| 5β-Tetrahydrocortisol (ng/mL) | 1.15 ± 2.97 | 0.82 ± 2.03 | 1.69 ± 4.04 | 0.400 |
| 6β-Hydroxycortisol (ng/mL) | 0.25 ± 0.55 | 0.2 ± 0.44 | 0.34 ± 0.7 | 1.000 |
| Cortisone (ng/mL) | 1.13 ± 1.34 | 1.11 ± 1.48 | 1.16 ± 1.1 | 0.760 |
| 20α-Dihydrocortisone (ng/mL) | 0.24 ± 0.33 | 0.22 ± 0.36 | 0.26 ± 0.26 | 0.580 |
| 20β-Dihydrocortisone (ng/mL) | 0.11 ± 0.22 | 0.11 ± 0.25 | 0.13 ± 0.15 | 0.500 |
| Corticosterone (ng/mL) | 0.15 ± 0.35 | 0.15 ± 0.42 | 0.15 ± 0.19 | 0.500 |
| 11-dehydrocorticosterone (ng/mL) | 0.01 ± 0.03 | 0.01 ± 0.03 | 0.02 ± 0.02 | 0.760 |
| Testosterone (ng/mL) | 0.2 ± 0.16 | 0.21 ± 0.18 | 0.18 ± 0.13 | 0.940 |
| Androstenedione (ng/mL) | 0.08 ± 0.17 | 0.06 ± 0.05 | 0.11 ± 0.27 | 0.810 |
| Progesterone (ng/mL) | 0.03 ± 0.08 | 0.04 ± 0.11 | 0.02 ± 0.02 | 0.700 |
| Kynurenine/Tryptophan | 0.16 ± 0.19 | 0.14 ± 0.19 | 0.2 ± 0.19 | 0.290 |
| 5-hydroxyindoleacetic acid/Tryptophan | 0.01 ± 0.02 | 0.004 ± 0.02 | 0.01 ± 0.02 | 0.390 |
| 3-hydroxykynurenine/Kynurenine | 0.02 ± 0.04 | 0.02 ± 0.04 | 0.03 ± 0.05 | 0.600 |
| Kynurenic acid/Kynurenine | 0.08 ± 0.09 | 0.07 ± 0.06 | 0.11 ± 0.12 | 0.870 |
| Σ Branched-chain amino acids/Σ Aromatic amin | 2.43 ± 0.57 | 2.46 ± 0.57 | 2.39 ± 0.58 | 0.850 |
| Creatine/Creatinine | 1.8 ± 1.35 | 1.96 ± 1.47 | 1.55 ± 1.13 | 0.710 |
| Glutamine/Glutamic acid | 7.2 ± 4.02 | 7.9 ± 4.31 | 6.13 ± 3.28 | 0.390 |
| Trimethylamine N-oxide/Choline | 0.0048 ± 0.02 | 0.0026 ± 0.01 | 0.01 ± 0.03 | 0.850 |
| Hexosylceramide 16:0/Ceramide 16:0 | 19.4 ± 16 | 20.34 ± 18.53 | 17.95 ± 11.12 | 0.980 |
| Hexosylceramide 18:0/Ceramide 18:0 | 0.39 ± 0.23 | 0.43 ± 0.26 | 0.33 ± 0.18 | 0.530 |
| Hexosylceramide 20:0/Ceramide 20:0 | 0.0004 ± 0.00031 | 0.00045 ± 0 | 0.00035 ± 0.00028 | 0.500 |
| Hexosylceramide 22:0/Ceramide 22:0 | 0.02 ± 0.02 | 0.03 ± 0.02 | 0.02 ± 0.02 | 0.580 |
| Hexosylceramide 24:0/Ceramide 24:0 | 0.02 ± 0.01 | 0.01 ± 0.01 | 0.02 ± 0.01 | 0.850 |
| Hexosylceramide 24:1/Ceramide 24:1 | 2.1 ± 1.13 | 2.14 ± 1.2 | 2.04 ± 1.02 | 0.940 |
| Lactic acid/Pyruvic acid | 8.4 ± 4.19 | 8.54 ± 4.2 | 8.19 ± 4.22 | 0.850 |
| α-Ketoglutaric acid/Citric acid | 0.32 ± 0.16 | 0.35 ± 0.19 | 0.28 ± 0.1 | 0.600 |
| Succinic acid/α-Ketoglutaric acid | 0.31 ± 0.17 | 0.3 ± 0.17 | 0.32 ± 0.17 | 0.850 |
| Malic acid/Succinic acid | 2.49 ± 1 | 2.43 ± 0.91 | 2.59 ± 1.13 | 0.940 |
| Citric acid/Malic acid | 5.85 ± 2.03 | 5.72 ± 2.08 | 6.05 ± 1.97 | 0.710 |
| Citric acid/Pyruvic acid | 0.16 ± 0.13 | 0.16 ± 0.13 | 0.17 ± 0.13 | 0.870 |
| Citric acid/Lactic acid | 0.02 ± 0.01 | 0.02 ± 0.01 | 0.02 ± 0.01 | 0.850 |
| Glutamic acid/α-Ketoglutaric acid | 4.69 ± 4.49 | 4.39 ± 4.22 | 5.17 ± 4.91 | 0.680 |
| α-Ketoisocaproic acid/Leucine | 7.8e-05 ± 4.6e-05 | 7.4e-05 ± 3.9e-05 | 8.5e-05 ± 5.6e-05 | 0.940 |
| Cortisol/Cortisone | 10.1 ± 7.65 | 8.87 ± 4.96 | 12.12 ± 10.48 | 0.710 |
| 20α-Dihydrocortisol/Cortisol | 0.09 ± 0.08 | 0.08 ± 0.06 | 0.11 ± 0.11 | 0.620 |
| 20β-Dihydrocortisol/Cortisol | 0.11 ± 0.07 | 0.09 ± 0.06 | 0.13 ± 0.08 | 0.340 |
| 20α-Dihydrocortisone/Cortisone | 0.21 ± 0.12 | 0.19 ± 0.08 | 0.25 ± 0.16 | 0.700 |
| 20β-Dihydrocortisone/Cortisone | 0.09 ± 0.07 | 0.08 ± 0.07 | 0.1 ± 0.08 | 0.450 |
| *Values reflect p-values adjusted using the Benjamini & Hochberg method. | | | | |

| **A.** Validation Cohort |  |  |  |  |
| --- | --- | --- | --- | --- |
|  | **ALL** (n = 22) | **SUCCESS** (n = 13) | **FAILURE** (n = 9) | **p-value*** |
| Valine (ng/mL) | 9036.23 ± 2909.24 | 8689.49 ± 2356.86 | 9498.55 ± 3619.46 | 0.950 |
| Leucine (ng/mL) | 24957.44 ± 7261.17 | 23784.94 ± 5803.91 | 26520.77 ± 8982.35 | 0.970 |
| Isoleucine (ng/mL) | 11882.39 ± 3967.32 | 11102.28 ± 2944.45 | 12922.55 ± 5029.71 | 0.970 |
| Methionine (ng/mL) | 3614.76 ± 1697.01 | 3457.42 ± 1606.84 | 3824.55 ± 1887.53 | 0.990 |
| Tryptophan (ng/mL) | 6117.05 ± 2081.95 | 6275.39 ± 1872.74 | 5905.93 ± 2434.37 | 1.000 |
| Serotonin (ng/mL) | 9.79 ± 13.91 | 12.81 ± 17.3 | 5.75 ± 6.31 | 0.820 |
| 5-hydroxyindoleacetic acid (ng/mL) | 14.81 ± 16.35 | 9.68 ± 4.03 | 21.66 ± 23.53 | 0.170 |
| Kynurenine (ng/mL) | 741.18 ± 379.92 | 527.05 ± 106.24 | 1026.69 ± 429.94 | 0.072 |
| 3-hydroxykynurenine (ng/mL) | 35.73 ± 55.69 | 18.77 ± 19.84 | 58.34 ± 78.78 | 0.580 |
| Kynurenic acid (ng/mL) | 46.51 ± 39.11 | 32.82 ± 16.18 | 64.76 ± 52.99 | 0.260 |
| Anthranilic acid (a.u.) | 12285.78 ± 8178.43 | 8529.65 ± 3487.89 | 17293.94 ± 10055.34 | 0.072 |
| Phenylalanine (ng/mL) | 7291.94 ± 1680.8 | 7055.55 ± 1518.21 | 7607.13 ± 1922.98 | 0.970 |
| Tyrosine (ng/mL) | 6157.99 ± 1922.88 | 6214.87 ± 1931.55 | 6082.14 ± 2025.44 | 1.000 |
| Nicotinamide (a.u.) | 0.21 ± 0.22 | 0.26 ± 0.27 | 0.14 ± 0.09 | 0.910 |
| Creatinine (ng/mL) | 1484.7 ± 1469.84 | 1121.27 ± 350.65 | 2009.66 ± 2227.56 | 0.470 |
| Creatine (ng/mL) | 2027.17 ± 1350.35 | 1660.23 ± 1306.71 | 2557.2 ± 1300.16 | 0.670 |
| Choline (ng/mL) | 127.4 ± 82.3 | 118.48 ± 63.18 | 140.27 ± 107.12 | 1.000 |
| Glutamine (ng/mL) | 17785.99 ± 5807.28 | 18321.65 ± 6747.2 | 17012.25 ± 4370.37 | 1.000 |
| Glutamic acid (ng/mL) | 1745.53 ± 806.8 | 1707.68 ± 877.65 | 1800.2 ± 739.98 | 1.000 |
| Acetilcarnitine (ng/mL) | 282.13 ± 150.45 | 249.94 ± 92.11 | 328.63 ± 206.34 | 0.950 |
| Carnitine (ng/mL) | 1751.93 ± 552.54 | 1788.87 ± 597.06 | 1698.58 ± 511.16 | 1.000 |
| Trimethylamine N-oxide (a.u.) | 0.29 ± 0.52 | 0.29 ± 0.54 | 0.28 ± 0.53 | 1.000 |
| Betaine (a.u.) | 2.3 ± 1.19 | 2.14 ± 1.11 | 2.54 ± 1.32 | 0.810 |
| Diacylglycerol 16:1,16:1 (a.u.) | 1.46 ± 0.79 | 1.57 ± 0.95 | 1.31 ± 0.5 | 1.000 |
| Diacylglycerol 16:0,16:0 (ng/mL) | 973.34 ± 307.11 | 927.85 ± 265.32 | 1039.05 ± 365.75 | 0.980 |
| Diacylglycerol 16:0,18:2 (a.u.) | 15.48 ± 7.5 | 13.1 ± 5.88 | 18.93 ± 8.55 | 0.350 |
| Diacylglycerol 18:1,16:0 (a.u.) | 65.12 ± 38.04 | 56.21 ± 27.22 | 77.99 ± 48.7 | 0.730 |
| Diacylglycerol 16:0,18:0 (ng/mL) | 4682.93 ± 2672.47 | 4327.67 ± 2452.31 | 5196.08 ± 3037.42 | 1.000 |
| Diacylglycerol 18:2,18:2 (a.u.) | 10.73 ± 6.54 | 8.62 ± 4.93 | 13.78 ± 7.62 | 0.620 |
| Diacylglycerol 18:0,18:2 (ng/mL) | 1327.11 ± 938.03 | 1032.32 ± 613.65 | 1752.93 ± 1183.09 | 0.350 |
| Diacylglycerol 18:1,18:1 (ng/mL) | 2368.59 ± 799.49 | 2442.02 ± 807.73 | 2262.52 ± 823.27 | 0.980 |
| Diacylglycerol 18:0,18:1 (a.u.) | 4.17 ± 5.95 | 3.27 ± 3.53 | 5.48 ± 8.42 | 0.970 |
| Diacylglycerol 18:0,18:0 (ng/mL) | 105.1 ± 38.56 | 109.66 ± 43.85 | 98.52 ± 30.6 | 0.790 |
| Diacylglycerol 18:0,20:4 (ng/mL) | 533.79 ± 437.72 | 495.16 ± 349.62 | 589.58 ± 560.06 | 0.950 |
| Monoacylglycerol 14:0 (a.u.) | 24.73 ± 16.61 | 22.13 ± 15.03 | 28.49 ± 18.93 | 0.810 |
| Monoacylglycerol 16:0 (ng/mL) | 72127.92 ± 41177.37 | 64794.86 ± 44819.32 | 82720.12 ± 34987.56 | 0.790 |
| Monoacylglycerol 18:2 (ng/mL) | 2786.87 ± 1386.72 | 2353.3 ± 1014.49 | 3413.14 ± 1660.52 | 0.720 |
| Monoacylglycerol 18:1 (ng/mL) | 3227.14 ± 1360.48 | 2977.29 ± 911.94 | 3588.04 ± 1833.91 | 0.980 |
| Monoacylglycerol 20:4 (ng/mL) | 302.9 ± 176.38 | 270.99 ± 96.83 | 349 ± 252.1 | 1.000 |
| Ceramide 14:0 (a.u.) | 9.89 ± 5.11 | 8.22 ± 3.48 | 12.29 ± 6.28 | 0.540 |
| Ceramide 16:0 (ng/mL) | 1115.31 ± 358.49 | 1069.87 ± 322.04 | 1180.93 ± 416.64 | 1.000 |
| Ceramide 18:0 (ng/mL) | 666.33 ± 315.79 | 756.87 ± 326.38 | 535.56 ± 263.49 | 0.630 |
| Ceramide 20:0 (ng/mL) | 1701.35 ± 1791.77 | 2125.43 ± 2053.95 | 1088.78 ± 1176.82 | 0.780 |
| Ceramide 22:0 (ng/mL) | 811.82 ± 456.52 | 648.61 ± 266.36 | 1047.58 ± 578.65 | 0.630 |
| Ceramide 24:0 (ng/mL) | 1263.29 ± 401.9 | 1275.83 ± 308.17 | 1245.18 ± 530.02 | 0.870 |
| Ceramide 24:1 (ng/mL) | 2631.19 ± 702.19 | 2766.48 ± 718.56 | 2435.77 ± 668.65 | 0.840 |
| Hexosylceramide 14:0 (a.u.) | 1.08 ± 0.71 | 0.92 ± 0.68 | 1.29 ± 0.73 | 0.730 |
| Hexosylceramide 16:0 (ng/mL) | 29969.38 ± 15640.68 | 28473.69 ± 16140.67 | 32129.81 ± 15572.03 | 0.840 |
| Hexosylceramide 18:0 (ng/mL) | 166.1 ± 58.17 | 170.21 ± 64.32 | 160.15 ± 51.09 | 1.000 |
| Hexosylceramide 20:0 (a.u.) | 0.21 ± 0.09 | 0.22 ± 0.1 | 0.19 ± 0.09 | 0.980 |
| Hexosylceramide 22:0 (a.u.) | 8.12 ± 4.66 | 8.09 ± 4.74 | 8.16 ± 4.83 | 1.000 |
| Hexosylceramide 24:0 (a.u.) | 15.2 ± 11.36 | 15.07 ± 8.25 | 15.39 ± 15.39 | 0.940 |
| Hexosylceramide 16:1 (a.u.) | 3.5 ± 1.66 | 3.66 ± 1.62 | 3.29 ± 1.79 | 1.000 |
| Hexosylceramide 18:1 (ng/mL) | 416.4 ± 274.17 | 446.29 ± 320.97 | 373.23 ± 198.09 | 1.000 |
| Hexosylceramide 24:1 (ng/mL) | 5722.49 ± 2274.25 | 5494.75 ± 1984.29 | 6051.45 ± 2732.21 | 1.000 |
| Lysophosphatidylcholine 14:0 (ng/mL) | 1290.18 ± 867.57 | 1161.6 ± 488.74 | 1475.91 ± 1245.71 | 1.000 |
| Lysophosphatidylcholine 15:0 (ng/mL) | 1313.24 ± 722.71 | 1251.74 ± 503.53 | 1402.07 ± 987.79 | 1.000 |
| Lysophosphatidylcholine 16:0 (ng/mL) | 129021.97 ± 35260.57 | 126104.11 ± 24249.54 | 133236.66 ± 48454.14 | 1.000 |
| Lysophosphatidylcholine 18:0 (ng/mL) | 74418.03 ± 23510.19 | 72960.4 ± 17369.58 | 76523.5 ± 31463.01 | 1.000 |
| Lysophosphatidylcholine 20:0 (a.u.) | 0.65 ± 0.39 | 0.59 ± 0.29 | 0.75 ± 0.5 | 0.970 |
| Lysophosphatidylcholine 22:0 (ng/mL) | 140.7 ± 245.2 | 86.75 ± 63.05 | 218.64 ± 374.57 | 0.910 |
| Lysophosphatidylcholine 22:6 (a.u.) | 5.77 ± 7.99 | 4.22 ± 1.55 | 8 ± 12.43 | 0.940 |
| Lysophosphatidylcholine 16:1 (a.u.) | 1.51 ± 0.78 | 1.47 ± 0.74 | 1.57 ± 0.87 | 1.000 |
| Lysophosphatidylcholine 18:1 (ng/mL) | 94032.47 ± 32694.78 | 88632.96 ± 23754.51 | 101831.76 ± 42939.09 | 0.980 |
| Lysophosphatidylcholine 18:3 (a.u.) | 0.35 ± 0.23 | 0.3 ± 0.13 | 0.41 ± 0.32 | 1.000 |
| Lysophosphatidylcholine 18:2 (a.u.) | 27.53 ± 8.46 | 25.66 ± 6.43 | 30.23 ± 10.59 | 0.730 |
| Lysophosphatidylcholine 20:1 (a.u.) | 0.29 ± 0.13 | 0.27 ± 0.1 | 0.31 ± 0.18 | 1.000 |
| Lysophosphatidylcholine 20:2 (a.u.) | 0.6 ± 0.28 | 0.54 ± 0.2 | 0.69 ± 0.37 | 0.810 |
| Lysophosphatidylcholine 20:3 (a.u.) | 3.24 ± 1.56 | 3.05 ± 1.29 | 3.5 ± 1.94 | 0.970 |
| Lysophosphatidylcholine 20:4 (a.u.) | 8.31 ± 3.11 | 8.27 ± 2.84 | 8.35 ± 3.65 | 1.000 |
| Lysophosphatidylcholine 20:5 (a.u.) | 0.79 ± 1.24 | 0.45 ± 0.23 | 1.28 ± 1.88 | 1.000 |
| Lysophosphatidylcholine 22:4 (a.u.) | 0.86 ± 0.88 | 0.73 ± 0.64 | 1.05 ± 1.17 | 0.940 |
| Lysophosphatidylcholine 22:5 (a.u.) | 1.68 ± 2.81 | 1.08 ± 0.8 | 2.54 ± 4.28 | 1.000 |
| Lactic acid (ng/mL) | 113064.62 ± 76941.28 | 117091.34 ± 94717.22 | 107248.24 ± 44927.29 | 0.970 |
| Pyruvic acid (ng/mL) | 22275.54 ± 8995.32 | 22171.59 ± 9313.54 | 22425.69 ± 9069.06 | 1.000 |
| Citric acid (ng/mL) | 1689.45 ± 323.22 | 1636.55 ± 296.53 | 1765.85 ± 362.26 | 0.940 |
| α-Ketoglutaric acid (ng/mL) | 728.75 ± 297.32 | 708.21 ± 341.4 | 758.4 ± 235.67 | 0.790 |
| Succinic acid (ng/mL) | 126.34 ± 71.53 | 110.29 ± 50.21 | 149.52 ± 92.88 | 0.870 |
| Malic acid (ng/mL) | 374.34 ± 172.23 | 314.09 ± 146.01 | 461.38 ± 177.38 | 0.350 |
| α-Ketobutyric acid (ng/mL) | 708450.36 ± 406134.49 | 735171.34 ± 391507.09 | 669853.4 ± 447468.79 | 0.980 |
| α-Hydroxybutyric acid (ng/mL) | 34599650.96 ± 16587027.69 | 31905566.95 ± 13948983.58 | 38491105.66 ± 20038037.56 | 0.950 |
| β-Hydroxybutyric acid (ng/mL) | 9047448.74 ± 10076170.76 | 6215523.57 ± 4363492.84 | 13138007.32 ± 14356101.92 | 0.760 |
| α-Hydroxyglutaric acid (a.u.) | 0.03 ± 0.02 | 0.03 ± 0.01 | 0.04 ± 0.02 | 0.730 |
| Hippuric acid (ng/mL) | 11.25 ± 22.81 | 6.54 ± 3.96 | 18.05 ± 35.42 | 1.000 |
| α-Ketoisocaproic acid (a.u.) | 1.55 ± 0.81 | 1.56 ± 0.83 | 1.53 ± 0.83 | 1.000 |
| β-Hydroxy β-methylbutyric acid (a.u.) | 0.05 ± 0.03 | 0.04 ± 0.02 | 0.06 ± 0.04 | 0.810 |
| Propionic acid (a.u.) | 2.83 ± 1.29 | 3.27 ± 1.27 | 2.21 ± 1.1 | 0.480 |
| Butyric acid (a.u.) | 0.4 ± 0.14 | 0.41 ± 0.13 | 0.38 ± 0.16 | 0.950 |
| Valeric acid (a.u.) | 0.16 ± 0.09 | 0.18 ± 0.11 | 0.12 ± 0 | 0.540 |
| Isovaleric acid (a.u.) | 0.34 ± 0.24 | 0.45 ± 0.22 | 0.17 ± 0.14 | 0.072 |
| Octanoic acid (ng/mL) | 917210.98 ± 2252786.96 | 113689.06 ± 50481.92 | 2077853.76 ± 3279256.92 | 0.980 |
| Decanoic acid (ng/mL) | 2354686.65 ± 6078231.5 | 313121.81 ± 226677.27 | 5303613.65 ± 8963655.13 | 0.073 |
| Dodecanoic acid (ng/mL) | 3354275.93 ± 3945651.52 | 1538458.01 ± 1729994.48 | 5977124.02 ± 4824927.37 | 0.350 |
| Indole-3-acetic acid (a.u.) | 24526.29 ± 18019.14 | 25511.28 ± 21970.36 | 23103.52 ± 11153.98 | 1.000 |
| Indole-3-lactic acid (a.u.) | 5931.29 ± 3040.97 | 4947.1 ± 2339 | 7352.91 ± 3495.82 | 0.480 |
| Malonyl semialdehyde (ng/mL) | 29.48 ± 32.07 | 21.75 ± 11.25 | 40.66 ± 47.66 | 0.730 |
| Gluconic acid (ng/mL) | 20532.7 ± 79133.87 | 2873.29 ± 4572.43 | 46040.74 ± 123158.75 | 0.170 |
| Cortisol (ng/mL) | 7.91 ± 10.36 | 6.73 ± 8.49 | 9.61 ± 12.97 | 1.000 |
| 20α-Dihydrocortisol (ng/mL) | 1.02 ± 2.43 | 0.6 ± 0.8 | 1.62 ± 3.72 | 1.000 |
| 20β-Dihydrocortisol (ng/mL) | 1.34 ± 3.28 | 0.79 ± 1.17 | 2.12 ± 5 | 1.000 |
| 5α-Tetrahydrocortisol (ng/mL) | 4.12 ± 12.37 | 2.04 ± 4 | 7.12 ± 18.98 | 1.000 |
| 5β-Tetrahydrocortisol (ng/mL) | 0.71 ± 1.36 | 0.47 ± 0.74 | 1.05 ± 1.94 | 0.960 |
| 6β-Hydroxycortisol (ng/mL) | 0.1 ± 0.18 | 0.05 ± 0.08 | 0.18 ± 0.25 | 0.560 |
| Cortisone (ng/mL) | 1.24 ± 1.7 | 0.94 ± 1.12 | 1.67 ± 2.31 | 1.000 |
| 20α-Dihydrocortisone (ng/mL) | 0.23 ± 0.29 | 0.18 ± 0.17 | 0.3 ± 0.4 | 1.000 |
| 20β-Dihydrocortisone (ng/mL) | 0.11 ± 0.16 | 0.08 ± 0.09 | 0.17 ± 0.23 | 0.980 |
| Corticosterone (ng/mL) | 0.19 ± 0.45 | 0.11 ± 0.2 | 0.3 ± 0.66 | 0.980 |
| 11-dehydrocorticosterone (ng/mL) | 0.02 ± 0.04 | 0.01 ± 0.01 | 0.03 ± 0.06 | 0.870 |
| Testosterone (ng/mL) | 0.19 ± 0.22 | 0.17 ± 0.17 | 0.23 ± 0.27 | 0.980 |
| Androstenedione (ng/mL) | 0.09 ± 0.14 | 0.05 ± 0.05 | 0.14 ± 0.21 | 0.580 |
| Progesterone (ng/mL) | 0.03 ± 0.03 | 0.02 ± 0.02 | 0.04 ± 0.05 | 0.460 |
| Kynurenine/Tryptophan | 0.15 ± 0.14 | 0.09 ± 0.03 | 0.23 ± 0.19 | 0.260 |
| 5-hydroxyindoleacetic acid/Tryptophan | 0.0035 ± 0.01 | 0.0017 ± 0.00092 | 0.01 ± 0.01 | 0.630 |
| 3-hydroxykynurenine/Kynurenine | 0.04 ± 0.04 | 0.04 ± 0.04 | 0.05 ± 0.05 | 1.000 |
| Kynurenic acid/Kynurenine | 0.06 ± 0.03 | 0.06 ± 0.03 | 0.06 ± 0.03 | 1.000 |
| Σ Branched-chain amino acids/Σ Aromatic amin | 2.37 ± 0.45 | 2.27 ± 0.43 | 2.5 ± 0.46 | 0.870 |
| Creatine/Creatinine | 1.79 ± 1.27 | 1.65 ± 1.45 | 1.99 ± 1 | 0.970 |
| Glutamine/Glutamic acid | 12.51 ± 8.76 | 13.95 ± 10.86 | 10.43 ± 4.04 | 0.980 |
| Trimethylamine N-oxide/Choline | 0.0021 ± 0.0025 | 0.0025 ± 0.003 | 0.0017 ± 0.0015 | 1.000 |
| Hexosylceramide 16:0/Ceramide 16:0 | 29.11 ± 16.31 | 28.77 ± 16.48 | 29.6 ± 17.03 | 1.000 |
| Hexosylceramide 18:0/Ceramide 18:0 | 0.31 ± 0.17 | 0.28 ± 0.18 | 0.36 ± 0.16 | 0.730 |
| Hexosylceramide 20:0/Ceramide 20:0 | 0.00028 ± 3e-04 | 0.00028 ± 0.00038 | 0.00029 ± 0.00016 | 0.760 |
| Hexosylceramide 22:0/Ceramide 22:0 | 0.01 ± 0.01 | 0.01 ± 0.01 | 0.01 ± 0.01 | 0.910 |
| Hexosylceramide 24:0/Ceramide 24:0 | 0.01 ± 0.01 | 0.01 ± 0.01 | 0.01 ± 0.01 | 1.000 |
| Hexosylceramide 24:1/Ceramide 24:1 | 2.29 ± 0.98 | 2.08 ± 0.89 | 2.59 ± 1.07 | 0.810 |
| Lactic acid/Pyruvic acid | 4.91 ± 1.42 | 4.98 ± 1.53 | 4.8 ± 1.31 | 1.000 |
| α-Ketoglutaric acid/Citric acid | 0.43 ± 0.15 | 0.43 ± 0.16 | 0.44 ± 0.13 | 1.000 |
| Succinic acid/α-Ketoglutaric acid | 0.19 ± 0.1 | 0.18 ± 0.1 | 0.2 ± 0.11 | 0.980 |
| Malic acid/Succinic acid | 3.24 ± 1.31 | 2.97 ± 0.98 | 3.64 ± 1.66 | 0.780 |
| Citric acid/Malic acid | 5.31 ± 2.2 | 5.83 ± 1.71 | 4.56 ± 2.69 | 0.480 |
| Citric acid/Pyruvic acid | 0.08 ± 0.03 | 0.08 ± 0.02 | 0.09 ± 0.04 | 0.980 |
| Citric acid/Lactic acid | 0.02 ± 0.01 | 0.02 ± 0.01 | 0.02 ± 0.01 | 1.000 |
| Glutamic acid/α-Ketoglutaric acid | 2.55 ± 1.12 | 2.55 ± 1.11 | 2.55 ± 1.21 | 1.000 |
| α-Ketoisocaproic acid/Leucine | 6.6e-05 ± 3.6e-05 | 6.9e-05 ± 3.7e-05 | 6.1e-05 ± 3.6e-05 | 0.950 |
| Cortisol/Cortisone | 6.48 ± 2.9 | 6.32 ± 2.64 | 6.71 ± 3.4 | 1.000 |
| 20α-Dihydrocortisol/Cortisol | 0.1 ± 0.09 | 0.1 ± 0.09 | 0.09 ± 0.1 | 0.910 |
| 20β-Dihydrocortisol/Cortisol | 0.14 ± 0.19 | 0.16 ± 0.23 | 0.12 ± 0.13 | 1.000 |
| 20α-Dihydrocortisone/Cortisone | 0.2 ± 0.1 | 0.21 ± 0.06 | 0.18 ± 0.13 | 0.720 |
| 20β-Dihydrocortisone/Cortisone | 0.09 ± 0.06 | 0.08 ± 0.04 | 0.1 ± 0.08 | 1.000 |
| *Values reflect p-values adjusted using the Benjamini & Hochberg method. | | | | |

| **A**.Training and Validation Cohorts |  |  |  |  |
| --- | --- | --- | --- | --- |
|  | **ALL** (n = 111) | **SUCCESS** (n = 67) | **FAILURE** (n = 44) | **p-value*** |
| Valine (ng/mL) | 9946.63 ± 3040.96 | 10149.29 ± 2764.16 | 9642.65 ± 3425.47 | 0.740 |
| Leucine (ng/mL) | 24951.34 ± 7675.27 | 25100.29 ± 6925.52 | 24727.92 ± 8760.26 | 0.740 |
| Isoleucine (ng/mL) | 13210.91 ± 4187.01 | 13296.13 ± 3938.04 | 13083.09 ± 4579.16 | 0.750 |
| Methionine (ng/mL) | 3241.52 ± 1432.25 | 3371.42 ± 1525.88 | 3046.69 ± 1271.08 | 0.630 |
| Tryptophan (ng/mL) | 6035.73 ± 1741.52 | 6267.57 ± 1721.88 | 5687.97 ± 1732.04 | 0.570 |
| Serotonin (ng/mL) | 9.2 ± 11 | 8.99 ± 11.36 | 9.52 ± 10.57 | 0.810 |
| 5-hydroxyindoleacetic acid (ng/mL) | 19.54 ± 43.09 | 12.4 ± 27.2 | 30.26 ± 58.24 | 0.160 |
| Kynurenine (ng/mL) | 776.66 ± 489.33 | 666.57 ± 379.67 | 941.8 ± 585.28 | 0.048 |
| 3-hydroxykynurenine (ng/mL) | 24.22 ± 50 | 13.49 ± 24.61 | 40.31 ± 70.57 | 0.270 |
| Kynurenic acid (ng/mL) | 87.25 ± 225.6 | 56.16 ± 131.02 | 133.88 ± 315.21 | 0.170 |
| Anthranilic acid (a.u.) | 13914.81 ± 11480.52 | 11148.98 ± 7454.7 | 18063.55 ± 14857.48 | 0.014 |
| Phenylalanine (ng/mL) | 7489.6 ± 1716.1 | 7304.98 ± 1564.87 | 7766.54 ± 1905.96 | 0.740 |
| Tyrosine (ng/mL) | 6520.62 ± 2128 | 6735.85 ± 2156.23 | 6197.78 ± 2067.15 | 0.620 |
| Nicotinamide (a.u.) | 0.26 ± 0.2 | 0.27 ± 0.22 | 0.24 ± 0.17 | 0.810 |
| Creatinine (ng/mL) | 1667.68 ± 1843.56 | 1322.81 ± 1212.97 | 2192.81 ± 2443.08 | 0.180 |
| Creatine (ng/mL) | 2366.27 ± 2629.71 | 2012.07 ± 1345.59 | 2905.61 ± 3797.14 | 0.740 |
| Choline (ng/mL) | 140.23 ± 89.91 | 126.75 ± 58.36 | 160.75 ± 121.39 | 0.740 |
| Glutamine (ng/mL) | 14860.73 ± 5062.74 | 15486.48 ± 5572.29 | 13907.88 ± 4046.05 | 0.490 |
| Glutamic acid (ng/mL) | 2395.13 ± 1458.6 | 2355.04 ± 1581.51 | 2456.17 ± 1263.78 | 0.690 |
| Acetilcarnitine (ng/mL) | 310.33 ± 226.9 | 289.74 ± 193.16 | 341.7 ± 269.75 | 0.740 |
| Carnitine (ng/mL) | 1725.51 ± 760.42 | 1751.62 ± 633.12 | 1685.76 ± 928.05 | 0.550 |
| Trimethylamine N-oxide (a.u.) | 0.43 ± 1.23 | 0.3 ± 0.69 | 0.62 ± 1.76 | 0.550 |
| Betaine (a.u.) | 2.48 ± 1.42 | 2.27 ± 0.83 | 2.8 ± 1.98 | 0.590 |
| Diacylglycerol 16:1,16:1 (a.u.) | 1.47 ± 0.81 | 1.51 ± 0.74 | 1.41 ± 0.92 | 0.620 |
| Diacylglycerol 16:0,16:0 (ng/mL) | 483.56 ± 360.67 | 491.8 ± 348.47 | 471.02 ± 382.25 | 0.780 |
| Diacylglycerol 16:0,18:2 (a.u.) | 18.61 ± 13.65 | 17.87 ± 12.06 | 19.72 ± 15.87 | 0.830 |
| Diacylglycerol 18:1,16:0 (a.u.) | 84.66 ± 56.9 | 84.26 ± 56.49 | 85.28 ± 58.18 | 1.000 |
| Diacylglycerol 16:0,18:0 (ng/mL) | 1440.93 ± 2030.06 | 1368.33 ± 1832.77 | 1551.5 ± 2316.46 | 0.980 |
| Diacylglycerol 18:2,18:2 (a.u.) | 5.89 ± 5.74 | 4.95 ± 4.15 | 7.33 ± 7.36 | 0.570 |
| Diacylglycerol 18:0,18:2 (ng/mL) | 1545.95 ± 1232.4 | 1477.23 ± 1231.95 | 1650.59 ± 1239.87 | 0.740 |
| Diacylglycerol 18:1,18:1 (ng/mL) | 2389.73 ± 1243.67 | 2413.83 ± 1262.15 | 2353.02 ± 1228.54 | 0.830 |
| Diacylglycerol 18:0,18:1 (a.u.) | 3.37 ± 3.91 | 3.07 ± 3.43 | 3.83 ± 4.56 | 0.590 |
| Diacylglycerol 18:0,18:0 (ng/mL) | 28.34 ± 42.26 | 28.35 ± 44.61 | 28.33 ± 38.9 | 0.810 |
| Diacylglycerol 18:0,20:4 (ng/mL) | 334.76 ± 319.91 | 275.42 ± 244.44 | 425.11 ± 395.29 | 0.350 |
| Monoacylglycerol 14:0 (a.u.) | 18.17 ± 20.15 | 18.53 ± 19.39 | 17.63 ± 21.49 | 0.760 |
| Monoacylglycerol 16:0 (ng/mL) | 20862.27 ± 32038.92 | 19715.56 ± 30232.23 | 22608.38 ± 34898.09 | 0.800 |
| Monoacylglycerol 18:2 (ng/mL) | 2472.95 ± 1997.54 | 2415.75 ± 2147.31 | 2560.05 ± 1765.59 | 0.740 |
| Monoacylglycerol 18:1 (ng/mL) | 3424.99 ± 2469.71 | 3461.76 ± 2601.54 | 3368.98 ± 2282.52 | 1.000 |
| Monoacylglycerol 20:4 (ng/mL) | 221.91 ± 185.93 | 206.02 ± 151.63 | 246.1 ± 228.38 | 0.980 |
| Ceramide 14:0 (a.u.) | 7.27 ± 7.66 | 6.43 ± 5.76 | 8.56 ± 9.82 | 0.740 |
| Ceramide 16:0 (ng/mL) | 1094.19 ± 645.27 | 972.04 ± 437.93 | 1280.19 ± 843.87 | 0.320 |
| Ceramide 18:0 (ng/mL) | 558.68 ± 352.16 | 536.28 ± 372.19 | 592.8 ± 320.42 | 0.550 |
| Ceramide 20:0 (ng/mL) | 946.69 ± 1013.38 | 946.51 ± 1151.21 | 946.96 ± 769.99 | 0.490 |
| Ceramide 22:0 (ng/mL) | 582.82 ± 493.1 | 532.47 ± 495.98 | 659.48 ± 484.2 | 0.390 |
| Ceramide 24:0 (ng/mL) | 1210.74 ± 650.74 | 1218.77 ± 666.5 | 1198.51 ± 633.39 | 1.000 |
| Ceramide 24:1 (ng/mL) | 2439.03 ± 1139.01 | 2302.81 ± 952.2 | 2646.45 ± 1361.68 | 0.660 |
| Hexosylceramide 14:0 (a.u.) | 0.81 ± 0.89 | 0.77 ± 0.99 | 0.87 ± 0.71 | 0.360 |
| Hexosylceramide 16:0 (ng/mL) | 20649.71 ± 15643.63 | 19573.01 ± 16452.66 | 22289.22 ± 14352.75 | 0.490 |
| Hexosylceramide 18:0 (ng/mL) | 167.27 ± 88.45 | 163.02 ± 79.75 | 173.75 ± 100.9 | 1.000 |
| Hexosylceramide 20:0 (a.u.) | 0.22 ± 0.14 | 0.22 ± 0.13 | 0.23 ± 0.15 | 1.000 |
| Hexosylceramide 22:0 (a.u.) | 7.46 ± 5.13 | 7.41 ± 5.38 | 7.54 ± 4.78 | 0.980 |
| Hexosylceramide 24:0 (a.u.) | 17.05 ± 12.48 | 16.44 ± 11.15 | 17.98 ± 14.35 | 1.000 |
| Hexosylceramide 16:1 (a.u.) | 3.71 ± 3.3 | 3.75 ± 3.71 | 3.65 ± 2.57 | 0.740 |
| Hexosylceramide 18:1 (ng/mL) | 304.66 ± 258.19 | 291.84 ± 284.44 | 324.18 ± 213.79 | 0.490 |
| Hexosylceramide 24:1 (ng/mL) | 4946.83 ± 2995.55 | 4682.47 ± 2725.82 | 5349.36 ± 3357.98 | 0.740 |
| Lysophosphatidylcholine 14:0 (ng/mL) | 1146.25 ± 888.43 | 1094.82 ± 789.67 | 1224.57 ± 1025.5 | 0.980 |
| Lysophosphatidylcholine 15:0 (ng/mL) | 1418.32 ± 1172.35 | 1322.75 ± 860.44 | 1563.83 ± 1530.91 | 1.000 |
| Lysophosphatidylcholine 16:0 (ng/mL) | 126584.08 ± 42350.09 | 124436.18 ± 33666.56 | 129854.75 ± 53200.29 | 1.000 |
| Lysophosphatidylcholine 18:0 (ng/mL) | 68960.38 ± 30396.37 | 64951.9 ± 21981.01 | 75064.19 ± 39481.7 | 0.740 |
| Lysophosphatidylcholine 20:0 (a.u.) | 0.68 ± 0.77 | 0.56 ± 0.48 | 0.86 ± 1.06 | 0.350 |
| Lysophosphatidylcholine 22:0 (ng/mL) | 97.92 ± 136.05 | 82.55 ± 72.57 | 121.31 ± 195.81 | 0.620 |
| Lysophosphatidylcholine 22:6 (a.u.) | 5.01 ± 4.67 | 4.58 ± 3.03 | 5.65 ± 6.4 | 1.000 |
| Lysophosphatidylcholine 16:1 (a.u.) | 1.31 ± 0.75 | 1.3 ± 0.75 | 1.31 ± 0.77 | 1.000 |
| Lysophosphatidylcholine 18:1 (ng/mL) | 78278.79 ± 29940.57 | 75279.88 ± 26928.7 | 82845.3 ± 33835.4 | 0.740 |
| Lysophosphatidylcholine 18:3 (a.u.) | 0.23 ± 0.21 | 0.22 ± 0.17 | 0.26 ± 0.25 | 0.990 |
| Lysophosphatidylcholine 18:2 (a.u.) | 22.26 ± 8.36 | 22.43 ± 8.19 | 22 ± 8.69 | 0.810 |
| Lysophosphatidylcholine 20:1 (a.u.) | 0.28 ± 0.2 | 0.24 ± 0.12 | 0.33 ± 0.27 | 0.340 |
| Lysophosphatidylcholine 20:2 (a.u.) | 0.39 ± 0.24 | 0.35 ± 0.18 | 0.45 ± 0.3 | 0.480 |
| Lysophosphatidylcholine 20:3 (a.u.) | 1.95 ± 1.18 | 1.93 ± 1.1 | 1.99 ± 1.31 | 1.000 |
| Lysophosphatidylcholine 20:4 (a.u.) | 6.41 ± 3.07 | 6.27 ± 2.79 | 6.64 ± 3.49 | 0.980 |
| Lysophosphatidylcholine 20:5 (a.u.) | 0.47 ± 0.62 | 0.37 ± 0.25 | 0.61 ± 0.92 | 0.660 |
| Lysophosphatidylcholine 22:4 (a.u.) | 0.6 ± 0.54 | 0.59 ± 0.48 | 0.6 ± 0.63 | 0.980 |
| Lysophosphatidylcholine 22:5 (a.u.) | 1.04 ± 1.39 | 0.93 ± 0.69 | 1.21 ± 2.04 | 1.000 |
| Lactic acid (ng/mL) | 127293.63 ± 70223.36 | 126803.9 ± 72250.04 | 128039.34 ± 67837.03 | 1.000 |
| Pyruvic acid (ng/mL) | 18186.96 ± 8580.17 | 18100.06 ± 9149.16 | 18319.28 ± 7734.21 | 0.980 |
| Citric acid (ng/mL) | 2109.25 ± 902.81 | 1996.89 ± 680.81 | 2280.33 ± 1150.67 | 0.660 |
| α-Ketoglutaric acid (ng/mL) | 682.17 ± 347.78 | 695.47 ± 382.83 | 661.93 ± 289.43 | 1.000 |
| Succinic acid (ng/mL) | 165.11 ± 76.75 | 159.94 ± 67.39 | 172.98 ± 89.4 | 0.820 |
| Malic acid (ng/mL) | 420.48 ± 286.65 | 383.82 ± 186.21 | 476.31 ± 389.48 | 0.670 |
| α-Ketobutyric acid (ng/mL) | 610112.88 ± 436997.03 | 581930.06 ± 412109.77 | 653027.61 ± 474046.9 | 0.800 |
| α-Hydroxybutyric acid (ng/mL) | 35201820 ± 16615856.4 | 34194002.4 ± 16031145.7 | 36736451.2 ± 17544638 | 0.740 |
| β-Hydroxybutyric acid (ng/mL) | 14048267 ± 18450914.2 | 10777273.1 ± 10557029.5 | 19029098.7 ± 25646880.4 | 0.540 |
| α-Hydroxyglutaric acid (a.u.) | 0.03 ± 0.02 | 0.02 ± 0.02 | 0.04 ± 0.03 | 0.120 |
| Hippuric acid (ng/mL) | 36.66 ± 87.78 | 21.25 ± 40.94 | 60.12 ± 127.3 | 0.350 |
| α-Ketoisocaproic acid (a.u.) | 1.85 ± 1.18 | 1.8 ± 1.06 | 1.93 ± 1.34 | 1.000 |
| β-Hydroxy β-methylbutyric acid (a.u.) | 0.09 ± 0.07 | 0.08 ± 0.05 | 0.1 ± 0.08 | 0.490 |
| Propionic acid (a.u.) | 4.52 ± 4.33 | 4.42 ± 2.99 | 4.68 ± 5.84 | 0.630 |
| Butyric acid (a.u.) | 0.35 ± 0.18 | 0.35 ± 0.17 | 0.35 ± 0.2 | 1.000 |
| Valeric acid (a.u.) | 0.37 ± 0.52 | 0.37 ± 0.39 | 0.38 ± 0.67 | 0.400 |
| Isovaleric acid (a.u.) | 0.84 ± 1.15 | 0.91 ± 1.19 | 0.73 ± 1.09 | 0.320 |
| Octanoic acid (ng/mL) | 516943.85 ± 1323373.35 | 203928.62 ± 103461.12 | 993580.66 ± 2019542.88 | 0.350 |
| Decanoic acid (ng/mL) | 1444205.72 ± 3634559.48 | 645865.69 ± 558057.15 | 2659859.85 ± 5550659.2 | 0.320 |
| Dodecanoic acid (ng/mL) | 4562445.14 ± 4094657.4 | 4159121.5 ± 4035331.26 | 5176597.04 ± 4154157.09 | 0.490 |
| Indole-3-acetic acid (a.u.) | 33370.82 ± 38036.48 | 30894.47 ± 35237.32 | 37141.61 ± 42084.78 | 0.740 |
| Indole-3-lactic acid (a.u.) | 6786.22 ± 6567.99 | 5448.76 ± 3511.6 | 8822.81 ± 9186.71 | 0.180 |
| Malonyl semialdehyde (ng/mL) | 39.34 ± 59.91 | 42.79 ± 70.44 | 34.09 ± 38.97 | 0.740 |
| Gluconic acid (ng/mL) | 7595.55 ± 36982.96 | 2180.09 ± 3411.31 | 15841.82 ± 58014.9 | 0.170 |
| Cortisol (ng/mL) | 9.34 ± 10.54 | 8.4 ± 9.93 | 10.84 ± 11.4 | 0.390 |
| 20α-Dihydrocortisol (ng/mL) | 1.13 ± 2.64 | 0.83 ± 1.4 | 1.62 ± 3.85 | 0.430 |
| 20β-Dihydrocortisol (ng/mL) | 1.31 ± 2.59 | 0.95 ± 1.63 | 1.88 ± 3.59 | 0.330 |
| 5α-Tetrahydrocortisol (ng/mL) | 3.64 ± 9.4 | 2.51 ± 5.88 | 5.43 ± 13.1 | 0.550 |
| 5β-Tetrahydrocortisol (ng/mL) | 1.06 ± 2.72 | 0.75 ± 1.85 | 1.55 ± 3.68 | 0.320 |
| 6β-Hydroxycortisol (ng/mL) | 0.22 ± 0.5 | 0.17 ± 0.4 | 0.3 ± 0.63 | 0.740 |
| Cortisone (ng/mL) | 1.15 ± 1.41 | 1.08 ± 1.41 | 1.27 ± 1.43 | 0.740 |
| 20α-Dihydrocortisone (ng/mL) | 0.24 ± 0.32 | 0.22 ± 0.33 | 0.27 ± 0.29 | 0.620 |
| 20β-Dihydrocortisone (ng/mL) | 0.11 ± 0.21 | 0.1 ± 0.23 | 0.14 ± 0.17 | 0.440 |
| Corticosterone (ng/mL) | 0.16 ± 0.37 | 0.14 ± 0.38 | 0.18 ± 0.34 | 0.410 |
| 11-dehydrocorticosterone (ng/mL) | 0.02 ± 0.03 | 0.01 ± 0.03 | 0.02 ± 0.03 | 0.620 |
| Testosterone (ng/mL) | 0.2 ± 0.17 | 0.2 ± 0.17 | 0.19 ± 0.17 | 1.000 |
| Androstenedione (ng/mL) | 0.08 ± 0.16 | 0.06 ± 0.05 | 0.12 ± 0.25 | 0.570 |
| Progesterone (ng/mL) | 0.03 ± 0.08 | 0.03 ± 0.1 | 0.03 ± 0.03 | 0.390 |
| Kynurenine/Tryptophan | 0.16 ± 0.18 | 0.13 ± 0.17 | 0.2 ± 0.19 | 0.045 |
| 5-hydroxyindoleacetic acid/Tryptophan | 0.01 ± 0.02 | 0.0037 ± 0.017 | 0.01 ± 0.02 | 0.170 |
| 3-hydroxykynurenine/Kynurenine | 0.03 ± 0.04 | 0.02 ± 0.04 | 0.03 ± 0.05 | 0.550 |
| Kynurenic acid/Kynurenine | 0.08 ± 0.08 | 0.07 ± 0.06 | 0.1 ± 0.11 | 0.870 |
| Σ Branched-chain amino acids/Σ Aromatic amin | 2.42 ± 0.55 | 2.43 ± 0.54 | 2.41 ± 0.56 | 1.000 |
| Creatine/Creatinine | 1.8 ± 1.33 | 1.9 ± 1.46 | 1.64 ± 1.11 | 0.870 |
| Glutamine/Glutamic acid | 8.25 ± 5.66 | 9.07 ± 6.5 | 7.01 ± 3.83 | 0.350 |
| Trimethylamine N-oxide/Choline | 0.0043 ± 0.019 | 0.0026 ± 0.0051 | 0.0069 ± 0.03 | 0.810 |
| Hexosylceramide 16:0/Ceramide 16:0 | 21.33 ± 16.45 | 21.98 ± 18.34 | 20.33 ± 13.2 | 1.000 |
| Hexosylceramide 18:0/Ceramide 18:0 | 0.38 ± 0.22 | 0.4 ± 0.25 | 0.34 ± 0.17 | 0.740 |
| Hexosylceramide 20:0/Ceramide 20:0 | 0.00038 ± 0.00031 | 0.00041 ± 0.00034 | 0.00033 ± 0.00026 | 0.660 |
| Hexosylceramide 22:0/Ceramide 22:0 | 0.02 ± 0.02 | 0.02 ± 0.02 | 0.02 ± 0.02 | 0.430 |
| Hexosylceramide 24:0/Ceramide 24:0 | 0.02 ± 0.01 | 0.01 ± 0.01 | 0.02 ± 0.01 | 0.840 |
| Hexosylceramide 24:1/Ceramide 24:1 | 2.14 ± 1.1 | 2.13 ± 1.14 | 2.15 ± 1.04 | 0.990 |
| Lactic acid/Pyruvic acid | 7.71 ± 4.05 | 7.85 ± 4.07 | 7.5 ± 4.04 | 0.740 |
| α-Ketoglutaric acid/Citric acid | 0.35 ± 0.16 | 0.37 ± 0.18 | 0.31 ± 0.12 | 0.660 |
| Succinic acid/α-Ketoglutaric acid | 0.29 ± 0.17 | 0.28 ± 0.17 | 0.3 ± 0.17 | 0.790 |
| Malic acid/Succinic acid | 2.64 ± 1.1 | 2.54 ± 0.94 | 2.8 ± 1.31 | 0.790 |
| Citric acid/Malic acid | 5.74 ± 2.07 | 5.74 ± 2 | 5.74 ± 2.19 | 1.000 |
| Citric acid/Pyruvic acid | 0.15 ± 0.12 | 0.15 ± 0.12 | 0.15 ± 0.12 | 0.770 |
| Citric acid/Lactic acid | 0.02 ± 0.01 | 0.02 ± 0.01 | 0.02 ± 0.01 | 0.740 |
| Glutamic acid/α-Ketoglutaric acid | 4.27 ± 4.14 | 4.03 ± 3.88 | 4.63 ± 4.53 | 0.740 |
| α-Ketoisocaproic acid/Leucine | 7.5e-05 ± 4.4e-05 | 7.3e-05 ± 3.8e-05 | "8e-05 ± 5.3e-05" | 1.000 |
| Cortisol/Cortisone | 9.37 ± 7.1 | 8.37 ± 4.69 | 10.96 ± 9.65 | 0.740 |
| 20α-Dihydrocortisol/Cortisol | 0.09 ± 0.08 | 0.09 ± 0.07 | 0.11 ± 0.1 | 0.740 |
| 20β-Dihydrocortisol/Cortisol | 0.11 ± 0.11 | 0.11 ± 0.12 | 0.13 ± 0.09 | 0.410 |
| 20α-Dihydrocortisone/Cortisone | 0.21 ± 0.12 | 0.19 ± 0.07 | 0.23 ± 0.16 | 0.950 |
| 20β-Dihydrocortisone/Cortisone | 0.09 ± 0.07 | 0.08 ± 0.06 | 0.1 ± 0.08 | 0.390 |
| *Values reflect p-values adjusted using the Benjamini & Hochberg method. | | | | |

**Table E8:** Summary AUCROC statistics for the Optimal, Clinical and ROX predictive models with a random 70% training – 30% test data partition.

a) Training set

| **Model** | **Min** | **Q1** | **Median** | **Mean** | **Q3** | **Max** |
| --- | --- | --- | --- | --- | --- | --- |
| **Optimal:**  **6 variables** | 0.74 | 0.81 | 0.83 | 0.83 | 0.85 | 0.90 |
| **Clinical :**  **3 variables** | 0.66 | 0.73 | 0.76 | 0.76 | 0.79 | 0.90 |
| **ROX** | 0.46 | 0.55 | 0.58 | 0.59 | 0.63 | 0.73 |

b) Test set

| **Model** | **Min** | **Q1** | **Median** | **Mean** | **Q3** | **Max** |
| --- | --- | --- | --- | --- | --- | --- |
| **Optimal:**  **6 variables** | 0.66 | 0.80 | 0.87 | 0.85 | 0.90 | 0.99 |
| **Clinical :**  **3 variables** | 0.58 | 0.72 | 0.76 | 0.77 | 0.82 | 0.91 |
| **ROX** | 0.31 | 0.55 | 0.63 | 0.62 | 0.68 | 0.84 |

**Figures**

**Figure E1.** Temporal distribution of included patients.

Distribution of patients with COVID-19-related AHRF from March 2020 to October 2021.
Light blue: Training cohort; dark blue: Validation cohort.

**Figure E2.** Frequency of variable combinations in six-variable models.

Histogram showing the frequency of variable combinations across 100 six-variable models.
More than 40% of models included the same combination: SpO₂/FiO₂ ratio, anthranilic acid, octanoic acid, 20β-dihydrocortisone, DAG(16:1), and DAG(18:0/20:4).

**Figure E3.** Distribution of selected variables.

|  |  |
| --- | --- |
|  |  |
|   Boxplots of selected variables at baseline (T0) in the Training cohort. Significance levels: *p < 0.05, **p < 0.01, ***p < 0.001, ****p < 0.0001 (Mann–Whitney U test).  Figure E4. Frequency of individual variable selection. |  |

Histogram showing the frequency of each variable across six-variable models.
SpO₂/FiO₂ ratio, anthranilic acid, and octanoic acid were the most frequently selected variables.

**Figure E5.** Permutation test (Best Prediction model).

Histogram of AUC values obtained from 1,000 permutations.
Red line: 95th percentile of null distribution; green line: observed AUC.

**Figure E6.** Permutation test (Clinical Practice model).

Histogram of AUC values from permutation testing.
Red line: 95th percentile; green line: observed AUC.

**Figure E7.** Age distribution before and after weighting.

Right panel: original age distribution; left panel: after overlap weighting.

**Figure E8.** Calibration plots.

|  |  |
| --- | --- |

Calibration curves for the Best Prediction model (right) and Clinical Practice model (left).
Error bars represent 68% binomial confidence intervals.
